# Spatial–temporal regulation of fatty alcohol biosynthesis in yeast

**DOI:** 10.1186/s13068-022-02242-7

**Published:** 2022-12-16

**Authors:** Ning Gao, Jiaoqi Gao, Wei Yu, Sijia Kong, Yongjin J. Zhou

**Affiliations:** 1 Division of Biotechnology, Dalian Institute of Chemical Physics, Chinese Academy of Sciences, Dalian, 116023 China; 2 CAS Key Laboratory of Separation Science for Analytical Chemistry, Dalian Institute of Chemical Physics, Chinese Academy of Sciences, Dalian, 116023 China; 3 Dalian Key Laboratory of Energy Biotechnology, Dalian Institute of Chemical Physics, Chinese Academy of Sciences, Dalian, 116023 China; 4 University of Chinese Academy of Sciences, Beijing, 100049 China

**Keywords:** Spatial–temporal regulation, Fatty alcohol, Metabolic engineering, Yeast biotechnology

## Abstract

**Background:**

Construction of efficient microbial cell factories is one of the core steps for establishing green bio-manufacturing processes. However, the complex metabolic regulation makes it challenging in driving the metabolic flux toward the product biosynthesis. Dynamically coupling the biosynthetic pathways with the cellular metabolism at spatial–temporal manner should be helpful for improving the production with alleviating the cellular stresses.

**Results:**

In this study, we observed the mismatch between fatty alcohol biosynthesis and cellular metabolism, which compromised the fatty alcohol production in *Saccharomyces cerevisiae*. To enhance the fatty alcohol production, we spatial-temporally regulated fatty alcohol biosynthetic pathway by peroxisomal compartmentalization (spatial) and dynamic regulation of gene expression (temporal). In particular, fatty acid/acyl-CoA responsive promoters were identified by comparative transcriptional analysis, which helped to dynamically regulate the expression of acyl-CoA reductase gene *MaFAR1* and improved fatty alcohol biosynthesis by 1.62-fold. Furthermore, enhancing the peroxisomal supply of acyl-CoA and NADPH further improved fatty alcohol production to 282 mg/L, 2.52 times higher than the starting strain.

**Conclusions:**

This spatial–temporal regulation strategy partially coordinated fatty alcohol biosynthesis with cellular metabolism including peroxisome biogenesis and precursor supply, which should be applied for production of other products in microbes.

**Supplementary Information:**

The online version contains supplementary material available at 10.1186/s13068-022-02242-7.

## Introduction

Construction of cell factories is considered as a feasible approach for sustainable supply of a variety of products of interests [1]. However, the complex and tight metabolic regulation makes it challenging in driving the metabolic flux toward product biosynthesis [2]. In particular, eukaryotic cells have compartmentalized metabolism in different sub-organelles, which brings some barriers for substrate channeling in supplying precursors and cofactors [3]. Furthermore, the cellular metabolism is dynamically controlled in response to cell state and extracellular signal [4]. Thus, coupling the spatial–temporal metabolism with the biosynthesis pathways should be helpful for improving the chemical production.

As an example, we here tried to couple the spatial–temporal fatty acid metabolism of *Saccharomyces cerevisiae* for producing fatty alcohol, an important oleo-chemical that can be used in detergents and personal care products with a global market of 6.8 billion USD [5]. Fatty alcohol can be bio-synthesized from free fatty acids (FFA) or activated fatty acids (fatty acyl-CoA and -ACP) through various reductase [6]. In *S. cerevisiae*, fatty acids are mainly de novo synthesized by cytosolic type I fatty acid synthase (FAS) as fatty acyl-CoAs, which can be transformed to functional or storage lipids. Under carbon-rich conditions or cell growth phase, fatty acid biosynthesis is very active and results in relatively high fatty acyl-CoA formation in cytosol. In the absence of the sufficient carbon sources, the storage lipids, such as triacylglycerols, will be broken down for energy supply through β-oxidation in peroxisomes [7]. This spatial–temporal regulation of fatty acid metabolism has been largely ignored for production of fatty acid derived chemicals, though pathway optimization and enhancing the supply of precursors and cofactors have been extensively explored [8, 9].

**Fig. 1:**
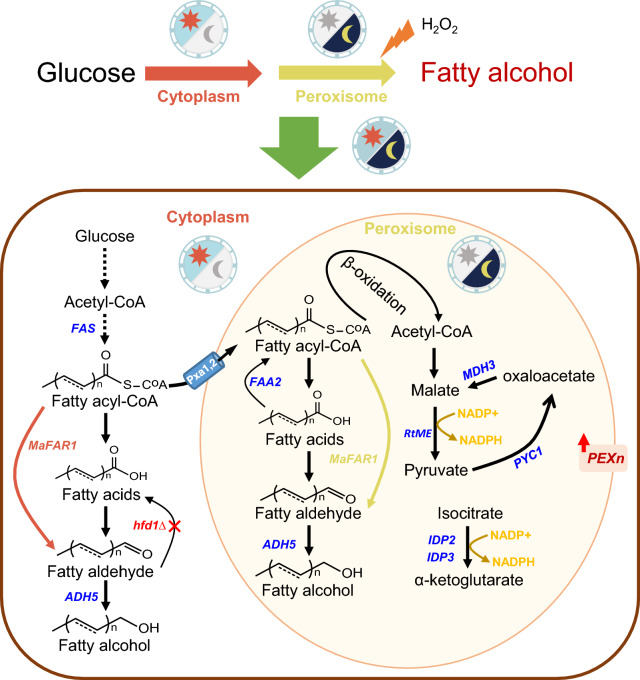
Engineering *S. cerevisiae* for enhanced fatty alcohol production via “spatial–temporal” regulation. In the early stage of fermentation (“the sun”), fatty acid biosynthesis was active for cell growth and a cytoplasmic fatty acyl-CoA reductase from *Marinobacter sp. ES-1* (*MaFAR1*) was expressed under the control of strong constitutive promoters (orange arrow), coupled with a disrupted hexadecenal dehydrogenase (*hfd1*Δ). With the consumption of glucose (“the moon”), fatty acid/acyl-CoA was accumulated, which activated peroxisomal *MaFAR1* by fatty acid/acyl-CoA responsive promoters (yellow arrow). The acyl-CoA transporter *PXA1*/*PXA2* and a peroxisomal copy of acyl-CoA synthase *FAA2* were overexpressed to increase peroxisomal acyl-CoA supply. The peroxisomal malate shuttling pathway (malic enzyme *RtME*, pyruvate carboxylase *PYC1* and malate dehydrogenase *MDH3*), and isocitrate dehydrogenase (*IDP2* and *IDP3*) were also targeted to peroxisomes to enhance the supply of peroxisomal NADPH. Peroxins (*PEX7* and *PEX28*) were overexpressed and H_2_O_2_ was added in culture to promote peroxisome biogenesis. Overall, the coordination of fatty alcohol biosynthesis, precursor and cofactor supply, and peroxisome biogenesis was implemented for spatial–temporal regulation of fatty alcohol biosynthesis

We here designed a spatial–temporal regulation strategy to couple fatty alcohol biosynthesis with the fatty acid metabolism through pathway compartmentalization and dynamic regulation by screening fatty acid/acyl-CoA responsive promoters (Fig. 1). A coordination of cytoplasmic and peroxisomal biosynthetic pathways was proved to be beneficial for improving fatty alcohol production by 2.52-fold (Additional file 1: Figure S1), which could be applied for production of other target products.

## Results

### Coordination of fatty alcohol biosynthesis

**Fig. 2:**
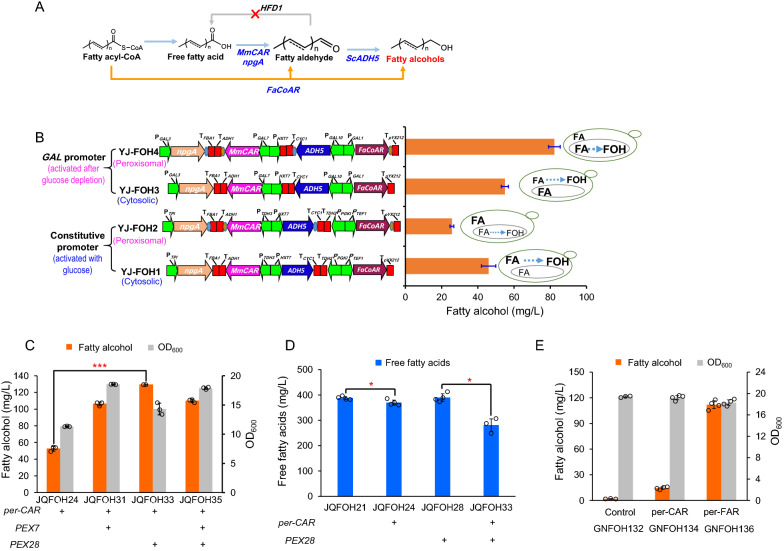
Coordinated fatty alcohol biosynthesis by optimizing a fatty acyl-CoA derived pathway. **A** Scheme of fatty alcohol biosynthetic pathways derived from fatty acids (*CAR* pathway, blue arrow) and acyl-CoA (*FaCoAR* or *FAR* pathway, orange arrow). **B** Partial coordination of fatty alcohol biosynthesis with peroxisome biogenesis and precursor supply promoted fatty alcohol production. Fatty alcohol titer of peroxisomal fatty alcohol biosynthetic pathway driven by constitutive promoters (P_*TDH3*_, P_*TEF1*_) was relatively low. Consistently, using *GA*L promoters of galactose catabolism (with deletion of *GAL80*) to drive the peroxisomal biosynthetic pathway significantly improved fatty alcohol production by 3.25 folds. FA, fatty acids; FOH, fatty alcohols. **C** Overexpression of peroxins *PEX7* and *PEX28* promoted fatty alcohol production of peroxisomal *CAR* pathway (*per-CAR*). **D** Residual free fatty acids of fatty alcohol producing strains with *per-CAR* pathway. **E** Comparison of fatty alcohol titer between *FAR* pathway and *CAR* pathway in peroxisomes. GNFOH132, the strain GN15 with an empty expression vector ; per-FAR, fatty alcohol production via peroxisomal *FAR* pathway. per-CAR, fatty alcohol production via peroxisomal *CAR* pathway. Data are presented as mean ± SD of three or four biologically independent samples with displayed data points. Red asterisks indicate statistical significance as determined using paired *t* test (*P < 0.05; *** < 0.001)

We previously constructed a chimeric fatty alcohol pathway by expressing a carboxylic acid reductase gene *MmCAR* and a fatty acyl-CoA reductase gene *FaCoAR*, which used both FFAs and fatty acyl-CoA as substrates [10]. We targeted this chimeric pathway to cytosol and peroxisomes, respectively (Fig. 2A). We observed that the peroxisomal pathway had much lower fatty alcohol production compared with the cytosolic pathway when using constitutive promoters for gene expression (Fig. 2B). It has been reported that constitutive promoters (P_*TEF1*_ for expressing *FaCoAR* and P_*TDH3*_ for expressing *MmCAR*) reached the highest strength in log-phase, and are relatively weak in stationary phase using glucose as the carbon source [11], while the number of peroxisomes shows the opposite pattern during cell growth [12]. This mismatch between fatty alcohol biosynthesis and peroxisome biogenesis might compromise fatty alcohol production. Consistently, using *GAL* promoters of galactose catabolism (with deletion of *GAL80*) to drive the peroxisomal biosynthetic pathway (strain YJ-FOH4) significantly improved fatty alcohol production by 3.25-fold (82.3 mg/L), which was also much higher than that of cytosolic pathway (strain YJ-FOH3). P_*GAL*_ promoters were shown to be activated at glucose exhaustion (Additional file 1: Figure S2), and their expression was supposed to be partially coordinated with peroxisome biogenesis [13]. This result suggested that spatial-temporally balancing the metabolic pathways was beneficial for improving the product biosynthesis.

The enhanced expression levels of peroxin genes *PEX7* and *PEX28* have been reported to increase the transport of peroxisomal matrix proteins [14] and peroxisome numbers [15] in *S. cerevisiae*, respectively, which was supposed to enhance fatty alcohol production in peroxisomes. Indeed, we found that overexpressing *PEX7* or *PEX28* promoted fatty alcohol production, and *PEX28* overexpression resulted in a 2.6-fold higher fatty alcohol titer (Fig. 2C). However, a large proportion of residual fatty acids was observed in fatty alcohol producing strains, and only up to 28% of fatty acids were utilized for fatty alcohol production (Fig. 2D), which might be attributed to the secreted fatty acids barely being re-utilized [16]. We thus tried to optimize and balance the fatty acyl-CoA derived pathway, which could avoid the accumulation of FFAs. Gene evaluation showed that *MaFAR1* from *Marinobacter* sp. ES-1 (Accession No. WP_022988858.1) achieved the highest fatty alcohol titer (Additional file 1: Figure S3), which was 7.7-fold higher than that from *CAR* pathway (Fig. 2E).

### Screening fatty acid/acyl-CoA responsive promoters

**Fig. 3:**
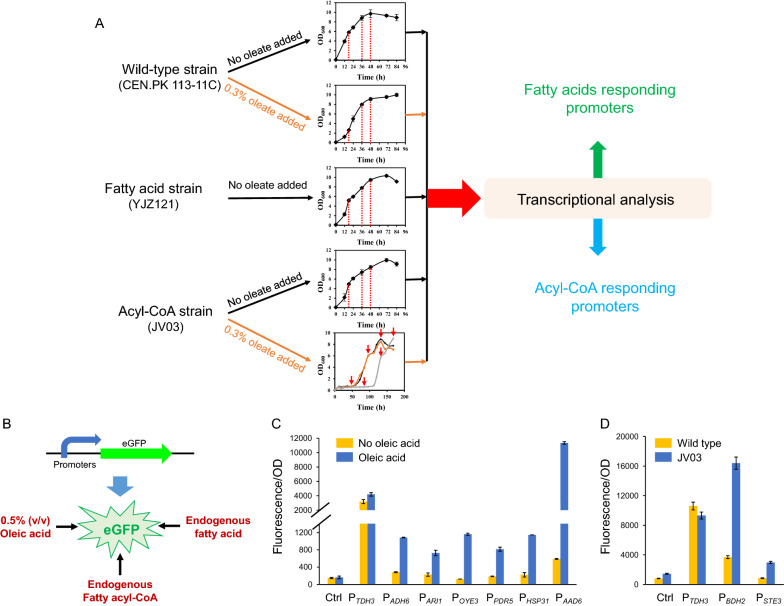
Screening of fatty acid/acyl-CoA responsive promoters. **A** Comparative transcriptional analysis to characterize endogenous promoters that respond to fatty acid/acyl-CoA. Wild-type strain (CEN.PK 113-11C) was cultivated in minimal medium without (black arrow), or with 0.3% (*v/v*) oleic acid (orange arrow), and samples were taken at 18 h, 36 h, and 48 h, respectively. FFA producing strain (YJZ121, black arrow) was cultivated in minimal medium, and samples were taken at 18 h, 36 h, and 48 h, respectively. Acyl-CoA producing strain (JV03) was cultivated in minimal medium without (black arrow), or with 0.3% (*v/v*) oleic acid (orange arrow). For strain JV03 without oleic acid, samples were taken at 18 h, 36 h, and 48 h, respectively. For strain JV03 with oleic acid, owing to oleic acid addition severely hindering cell growth, samples were taken at their own growth phase. **B** Candidate promoters were evaluated by fluorescence intensity of *eGFP* using exogenous FFA (0.5% oleic acid), endogenous FFA and acyl-CoA. **C** Promoters responsive to exogenous FFA. *eGFP* under the control of FFA responsive promoters was expressed in wild-type strain and cultivated in minimal media with or without 0.5% (*v/v*) oleic acid. **D** Promoters responsive to endogenous acyl-CoA. *eGFP* under the control of acyl-CoA responsive promoters was expressed in wild-type and acyl-CoA producing strain JV03, respectively. Data are presented as mean ± SD of three biological replicates

Screening endogenous fatty acid/acyl-CoA responsive promoters might be helpful for further improving fatty alcohol production by precisely balancing cellular fatty acid/acyl-CoA levels. We conducted comparative transcriptional analysis of wild-type, fatty acid producing strain YJZ121, and strain JV03 that was engineered for acyl-CoA accumulation [17] (Fig. 3A). The potential fatty acid/acyl-CoA responsive promoters were identified based on differentially expressed genes (DEGs) according to the following rules: RPKM (Reads Per Kilobase Million) value > 1500, fold change > 5, common genes among different conditions and sampling points, and non-metabolic genes. Under this criteria, 20 candidate promoters were supposed to be fatty acids (Additional file 1: Table S1) and/or fatty acyl-CoA responsive (Additional file 1: Table S2).

These promoters were further characterized using an *eGFP* in wild-type, strain YJZ121, and strain JV03 (Fig. 3B). Six promoters (P_*ADH6*_, P_*ARI1*_, P_*OYE3*_, P_*RDR5*_, P_*HSP31*_, and P_*AAD6*_) showed strong responsive activities to exogenous fatty acids (Fig. 3C), though most promoters demonstrated weak activities in wild-type cultivated in media with 0.5% (v/v) oleic acid (Additional file 1: Figure S4A). In particular, the activity of P_*AAD6*_ (promoter of a putative aryl-alcohol dehydrogenase) was 2.7-fold higher than that of the common strong constitutive promoter P_*eTDH3*_ in medium with 0.5% (v/v) oleic acid. Furthermore, promoters P_*ADH6*_ and P_*AAD6*_ were significantly induced by endogenous FFAs (wild-type strain CEN.PK 113-11C vs FFA overproducing strain YJZ121, Additional file 1: Figure S5) with considerable strength.

Two acyl-CoA responsive promoters, P_*BDH2*_ (promoter of a putative 3-hydroxybutyrate dehydrogenase 2) and P_*STE3*_ (promoter of receptor for a factor pheromone), were also characterized in both wild-type and JV03 (Fig. 3D and Additional file 1: Figure S4B). Promoter P_*BDH2*_ possessed relatively high activity in JV03, especially at the later stage of fermentation. Promoter P_*STE3*_ possessed a favorable response to acyl-CoA, in spite of a relatively low activity, which might be also useful for dynamically regulating fatty alcohol biosynthesis.

### Enhancing fatty alcohol production by dynamically regulating MaFAR1 expression

**Fig. 4:**
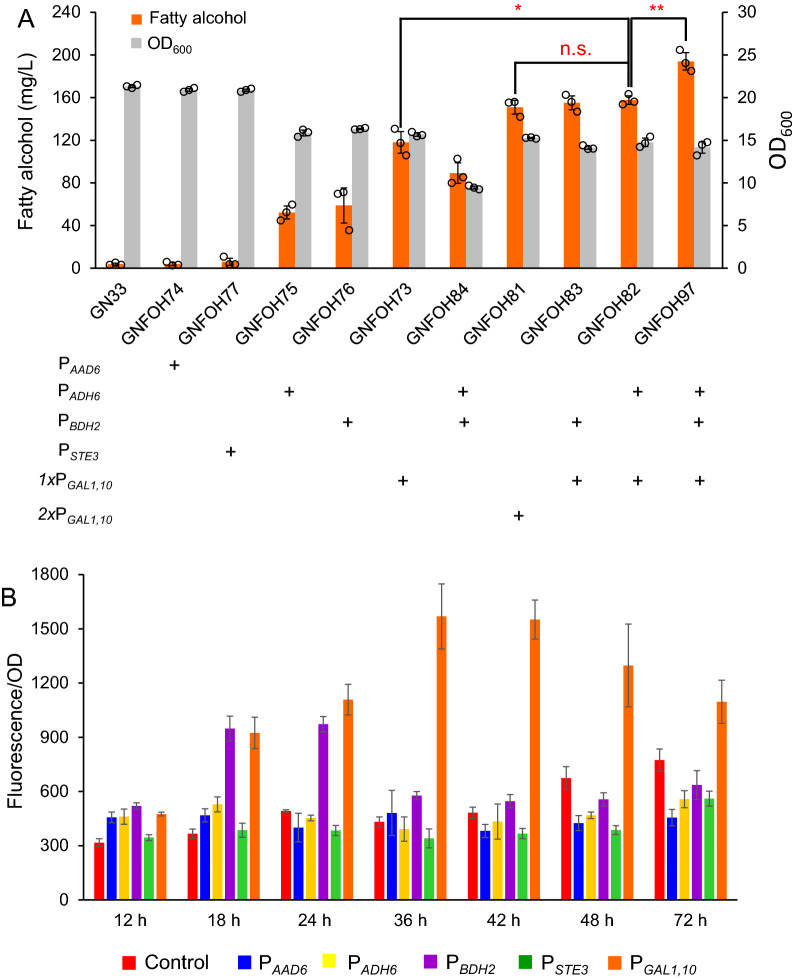
Dynamical regulation of fatty alcohol biosynthesis under control of fatty acid/acyl-CoA responsive promoters. **A** Fatty alcohol titer of strains with FAR pathway controlled by fatty acid/acyl-CoA responsive promoters and P_*GAL1,10*_. **B** Characterization of fatty acid/acyl-CoA responsive promoters by *eGFP* fluorescence intensity. *eGFP* controlled by various promoters, including P_*AAD6*_, P_*ADH6*_, P_*BDH2*_, P_*STE3*_, and P_*GAL1, 10*_, were integrated in control strain GN33, generating strains GN33-AAD6, GN33-ADH6, GN33-BDH2, GN33-STE3, and GN33-GAL_*1,10*_, respectively. Engineered strains were cultivated in minimal medium with 20 g/L glucose, and *eGFP* fluorescence was measured with different time to evaluate their specific responses. Data are presented as mean ± SD of three biologically independent yeast clones with displayed data points. Red asterisks indicate statistical significance as determined using paired *t* test (n.s. *p* > 0.05; *P < 0.05; ** < 0.01)

We then tried to enhance fatty alcohol production by dynamically regulating *MaFAR1* expression with the identified fatty acid/acyl-CoA responsive promoters. To avoid unstable expression of biosynthetic pathways on plasmids [18], the fatty alcohol biosynthetic genes were integrated into genome. To enable sufficient supply of precursor fatty acyl-CoA and cofactor NADPH, we constructed the fatty alcohol biosynthetic pathway in the FFA overproduction strain Y&Z036 [19] by a metabolic transforming strategy via knocking out gene *tesA* and restoring *FAA1*/*4* genes [20]. Furthermore, the peroxisome biogenesis was enhanced by over-expressing *PEX28,* which resulted in the chassis strain GN33 (Additional file 1: Figure S1). Expressing *MaFAR1* with P_*ADH6*_ and P_*BDH2*_ had higher fatty alcohol production than that of P_*AAD6*_, and P_*STE3*_, which, however, was only 50% of that with P_*GAL1,10*_. As expected, fatty alcohol titers were positively correlated with the promoter strengths (Fig. 4).

We further combined P_*ADH6*_ or P_*BDH2*_ with P_*GAL1,10*_ for expressing *MaFAR1*, which significantly improved fatty alcohol production compared with one-copy of P_*GAL1, 10*_ and also slightly higher than that with two copies of P_*GAL1,10*_ (Fig. 4A). These results suggested that fatty acid/acyl-CoA responsive promoters helped to synergistically promote fatty alcohol biosynthesis, especially the strength of P_*GAL1,10*_ was relatively low at the early stage of the cultivation, while P_*BDH2*_ was relatively higher (Fig. 4B). The strength compensation might be helpful for *MaFAR1* expression. Finally, expressing three copies of *MaFAR1* under the control of P_*ADH6*_, P_*BDH2*_ and P_*GAL1, 10*_ had the highest fatty alcohol production of 194 mg/L in strain GNFOH97 (Fig. 4A).

### Peroxisomal engineering for enhancing the supply of acyl-CoA and NADPH

**Fig. 5:**
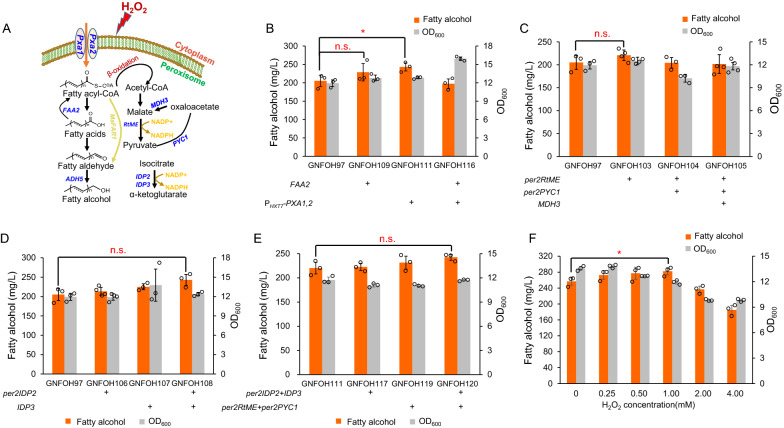
Enhanced supply of peroxisomal acyl-CoA and NADPH promoted fatty alcohol production. **A** Scheme of metabolic modifications to enhance the supply of peroxisomal acyl-CoA and NADPH. Acyl-CoA transporter *PXA1*/*PXA2* and a peroxisomal copy of acyl-CoA synthase *FAA2* were overexpressed to increase peroxisomal acyl-CoA supply. The malate shuttling pathway, including malic enzyme (*RtME*), pyruvate carboxylase (*PYC1*) and malate dehydrogenase (*MDH3*), and isocitrate dehydrogenase, *IDP2* and *IDP3*, were also targeted to peroxisomes to enhance peroxisomal NADPH supply. **B** Effect of overexpressed *PXA1*/*PXA2* and *FAA2* on cell growth and fatty alcohol titer. **C** Effect of introducing the peroxisomal malate shuttling pathway on cell growth and fatty alcohol titer. **D** Effect of overexpressed peroxisomal *IDP2* and *IDP3* on cell growth and fatty alcohol titer. **E** Combination of enhanced supply of peroxisomal acyl-CoA and NADPH promoted fatty alcohol production. **F** Effect of H_2_O_2_ with different concentrations on cell growth and fatty alcohol titer. The “per” represents gene adding the peroxisome targeting signal at the C-terminal. Data are presented as mean ± SD of three or four biologically independent samples with displayed data points. Red asterisks indicate statistical significance as determined using paired *t* test (n.s. *p* > 0.05; *P < 0.05)

In spite of the sufficient supply of cytosolic acyl-CoA and NADPH [21], the background strain was further engineered to achieve high-level production of fatty alcohols by enhancing the supply of peroxisomal acyl-CoA and NADPH. Firstly, the acyl-CoA transporter *PXA1*/*PXA2* and a peroxisomal copy of acyl-CoA synthase *FAA2* were overexpressed (Fig. 5A), which improved fatty alcohol titer by 12% and 19%, respectively (Fig. 5B). The combined overexpression of *PXA1*/*PXA2* and *FAA2* had a decreased fatty alcohol titer (Fig. 5B), which might be due to the disturbance of metabolic balance.

In addition, a peroxisomal malate shuttling pathway was constructed, including the essential genes of malic enzyme (*RtME*) [10], pyruvate carboxylase (*PYC1*) and malate dehydrogenase (*MDH3*) (Fig. 5A). Surprisingly, only the overexpression of *RtME* slightly increased fatty alcohol titer, and a combined overexpression of *PYC1* and *MDH3* had a negative effect (Fig. 5C). In particular, overexpression of *PYC1* strongly hindered cell growth and introduction of *MDH3* partially relieved the growth repression (comparing strains GNFOH105 with GNFOH104), but did not improve fatty alcohol production. Considering the specific fatty alcohol titer of strain GNFOH104 was higher than that of GNFOH103 and GNFOH105, a combined expression of *RtME* and *PYC1* was adopted to maintain the shuttling pathway.

It has been shown that overexpressing NADP^+^-dependent isocitrate dehydrogenase (*IDH*) was helpful for improving the production of FFAs [19]. We thus targeted the cytosolic NADP^+^-dependent Idp2 to peroxisome by adding the peroxisomal targeting signal per2 [22] and also overexpressed the peroxisomal isoform Idp3 (Fig. 5A). Overexpressing the encoding gene *perIDP2* or *IDP3* slightly improved fatty alcohol production and the combined overexpression improved fatty alcohol titer by 18.5% compared with the control strain GNFOH97 (Fig. 5D). Implementing these engineering strategies in strain GNFOH111 further improved fatty alcohol production (Fig. 5E). Interestingly, the introduction of malate shuttling pathway, coupled with the overexpressed isocitrate dehydrogenase, successfully improved fatty alcohol biosynthesis, which suggested that sufficient acyl-CoA supply required extra NADPH to drive a functional malate shuttling pathway. Combining all engineering strategies enabled fatty alcohol production of 242 mg/L, a 10% improvement compared with the control strain GNFOH111 (Fig. 5E).

Peroxisomes are inferred to be induced by ROS triggered chemicals like H_2_O_2_ [23], which might be helpful for enhancing fatty alcohol production in peroxisomes. Indeed, supplementing low concentrations of H_2_O_2_ (< 1 mM) in cell culture, slightly increased fatty alcohol titers with no obviously negative effects on cell growth of strain GNFOH120 (Fig. 5F) and the highest 282 mg/L fatty alcohol was obtained with 1 mM H_2_O_2_. However, higher concentrations of H_2_O_2_ decreased both biomass and fatty alcohols, which could be due to cell damage caused by H_2_O_2_. Therefore, H_2_O_2_ addition could be used as a feasible strategy to boost the efficiency of peroxisomal biosynthetic pathways.

We further expressed a copy of cytosolic *MaFAR1* with constitutive promoters P_*TEF1*_ and P_*TDH3*_ for converting the cytosolic fatty acid/acyl-CoA at the early stage, which slightly improved fatty alcohol production by 6.6% (Additional file 1: Figure S6A). This result suggested the acyl-CoA was mainly used for cell components such as phospholipids at the early stage and there was excessive acyl-CoA for fatty alcohol biosynthesis at the late fermentation stage. In addition, this spatial–temporal regulation via fatty acid/acyl-CoA responsive promoters and cellular compartmentalization did not significantly influence the composition of fatty alcohol (Additional file 1: Figure S6B), which resulted a partially coordinated of fatty alcohol biosynthesis and cellular metabolism in yeast.

## Discussion

Construction of heterologous artificial biosynthetic pathways always brings perturbation to the cellular metabolism, which, in turn, compromises the biosynthetic efficiency of target products. The cellular metabolism is spatial-temporally regulated to respond to extracellular signals or cell function, and construction of stable biosynthetic pathways might result in un-coordinated metabolic network as well as insufficient supply of precursors and cofactors. We here showed that spatial–temporal regulation of the fatty alcohol biosynthetic pathway significantly improved the production of fatty alcohols in model eukaryotic yeast *S. cerevisiae*.

It is well-recognized that the cellular metabolism is temporally regulated to respond to the nutrient availability, cell cycle and else [24]. In the exponential growth phase, the metabolic flux is mainly redirected for the synthesis of cellular components, and thus there is limited resources for biosynthesis of product of interest. Dynamically engineering biosynthetic pathways to separate from cell growth was helpful for overproduction [25–28]. We here also showed that dynamically regulating pathways with *GAL* promoters that are activated at glucose depletion, improved fatty alcohol production compared with that of constitutive promoters. In eukaryotes, cellular metabolism is distributed in sub-organelles for suitable physicochemical environments and condensed precursors/enzymes to avoid unnecessary interference. Metabolic compartmentalization has been extensively applied for improving the production of a variety of chemicals by targeting the biosynthetic pathways or specific enzymes into sub-organelles such as mitochondria [29, 30] and peroxisome [22, 31–33]. However, the temporal biogenesis of sub-organelle was largely ignored, and the mismatched biosynthetic pathways with sub-organelle biogenesis should largely compromise the engineering endeavor. For example, the peroxisomes mainly proliferate under the nutritional shortage or oxidative stress and are greatly repressed under the early growth stage with high-level of glucose [34, 35]. We actually observed that peroxisomal targeting the fatty alcohol biosynthesis pathway that was driven by the glucose activating promoters resulted low fatty alcohol production, while the partial coordination of peroxisome biogenesis using *GAL* promoters resulted much higher fatty alcohol production in peroxisomes. Further dynamically enhancing the supply of peroxisomal cofactors and precursor acyl-CoA significantly improved fatty alcohol production. These results clearly showed that spatial-temporally regulating the biosynthetic pathways was beneficial for production by smartly coping the complex cellular metabolism.

Dynamic regulation of metabolic pathways can be realized for genetically encoded control systems, among which the promoter-based transcriptional regulation is a convenient approach for construction of dynamic pathways. Several artificial promoters have been constructed to sense specific metabolites and improve the biosynthetic efficiency in bacteria [36, 37]. However, the complex structure of eukaryotic promoters makes it challenging to construct sensitive promoters for dynamic regulation. We here showed that transcriptomic analysis identified several promoters that respond to fatty acids/acyl-CoA, which helped to drive fatty alcohol production. Furthermore, these promoters significantly drove the production of 3-hydroxypropionic acid (3-HP), an important platform chemical (Additional file 1: Figure S7).

## Conclusions

This study proposed the spatial–temporal regulation of biosynthetic pathways to significantly improve the production of fatty alcohols and other acetyl-CoA derived chemicals, such as 3-HP. A coordination of cytoplasmic and peroxisomal biosynthetic pathways via pathway compartmentalization and dynamic regulation by screening fatty acid/acyl-CoA responsive promoters increased the product titer by up to 2.52-fold. We can image this strategy should be helpful for further improving fatty alcohol production in previously described cell factories such as oleaginous yeasts [9, 38] and *S. cerevisia*e [8], and may be used as an universal strategy for construction of efficient microbial cell factories.

## Materials and methods

### Strains and plasmids

Strains used in this study were listed in Additional file 1: Table S3 and Figure S1. Plasmids and primers were listed in Additional file 1: Table S4 and Table S5. Guide RNAs (gRNAs) sequences were listed in Additional file 1: Table S4. Fatty alcohol biosynthetic pathways were assembled on yeast chromosome, or plasmids pESC or pYX212 [39]. All *S. cerevisiae* strains used in this study were derived from CEN.PK113-11C (*MATa*; *SUC2*; *MAL2-8c*; *his3Δ1*; *ura3-52*;).

### Media and cultivation conditions

*S. cerevisiae* was generally cultivated at 30 °C in YPD medium consisting of 20 g/L glucose, 20 g/L peptone and 10 g/L yeast extract. Strains containing URA3/HIS3-based plasmids or gene expression cassettes were selected on synthetic dextrose media without uracil (SD-URA) or histidine (SD-HIS), which consisted of 20 g/L glucose and 6.7 g/L yeast nitrogen base (YNB, without amino acids). The SD + URA + HIS + 5-FOA plates consisting of 20 g/L glucose, 6.7 g/L yeast nitrogen base (YNB, without amino acids), 20 mg/L uracil, 20 mg/L histidine and 2 g/L 5-fluoroorotic acid were used to remove *URA3* maker. Fatty alcohol production was conducted in Delft minimal medium consisting of 14.4 g/L KH_2_PO_4_, 2.5 g/L (NH_4_)_2_SO_4_, 0.5 g/L MgSO_4_•7H_2_O, trace metal and vitamin solutions according to previous report [40], and 20 g/L glucose was added as carbon source, 60 mg/L uracil and 40 mg/L histidine were added if needed. *Escherichia coli* was cultivated at 37 °C in LB media (5 g/L yeast extract, 10 g/L tryptone and 10 g/L NaCl).

Commonly used reagents and primers were purchased from Sangon Biotech, Shanghai, China, unless otherwise specified. All codon optimized genes based on *S. cerevisiae* codon preference were synthesized by Exsyn-bio Technology Co., Ltd (Shanghai, China) and GENEWIZ (Suzhou, China).

### Genetic manipulation

The genetic manipulation was performed using CRISPR/Cas9 system as described previously [41]. *CAS9* gene was integrated into the genomic XI-5 site [42]. Seamless gene deletion was performed using corresponding guide RNA (gRNA) expressing plasmids and donor DNA. The donor DNA was constructed by overlap extension PCR (OE-PCR), of which homologous arms (HA, 200–500 bp) were amplified from the upstream and downstream of the target site. For gene integration, expression cassettes were constructed by OE-PCR to fuse the upstream HA, promoter, open reading frame (ORF), terminator and downstream HA [39], as shown in Figure S8. The integration modular was integrated at different neutral sites [43]. The proteins were targeted to peroxisome by adding the peroxisomal targeting signal (per1 and per2) at the C-terminal of the specific genes [22]. The gene expression cassettes or plasmids were transformed to *S. cerevisiae* according to previous report [44]. The mutants were verified by colony PCR. Transformants with successful integration were plated on SD + URA + HIS + 5-FOA plates to remove gRNA expressing plasmids that contain *URA3* maker. All codon-optimized genes were listed in Additional file 1: Table S6.

### Batch fermentation and metabolites quantification

Batch fermentations in shake flask were carried out as follows: three to four biological replicates were each inoculated into 2 mL YPD medium and incubated for about 24 h. Cell cultures were then inoculated again into 2 mL Delft minimal medium and cultivated for 24 h. The 24 h cultures were inoculate into 15 mL minimal medium to an optical density (OD_600_) of 0.1 in 100 mL-baffled flask, and cultivated at 30 °C, 220 rpm, for 72 h or 96 h. The OD_600_ of the culture was measured to evaluate cell growth.

For fatty alcohol, after 96 h cultivation, 1 mL cell culture was used to extract fatty alcohols as previously described [45]. Samples were analyzed by GC (Thermo fisher) as previously described [46]. Free fatty acids were extracted from 72 h cultivations and quantified as described in our previous study [10]. For quantification of 3-hydroxypropionic acid (3-HP), the samples were analyzed using high-performance liquid chromatography (Shimadzu LC-2030, Japan) as previously described [18].

### Screening of fatty acids/acyl-CoA-responsive promoters

To identify endogenous promoters that respond to fatty acids/acyl-CoA, transcriptional analysis was conducted in wild-type strain CEN.PK-113C, free fatty acid (FFA) producing strain YJZ121, and acyl-CoA accumulating strain JV03 [17]. Strains were pre-cultivated in minimal medium with 20 g/L glucose for 24 h, and then batch fermentation was carried out in 1.0 L bioreactors (Eppendorf) containing 0.3 L minimal medium with 20 g/L glucose. Wild-type strain was cultured in minimal media without, or with 0.3% (v/v) of oleic acid. Fatty acid producing strain was cultivated in minimal media without oleic acid. Acyl-CoA strain (JV03) was cultivated in minimal media without, or with 0.3% (v/v) of oleic acid. 0.1% (v/v) Tween80 was supplemented to dissolve oleic acid. Samples were taken at the early log phase, late log phase, and stationary phase. Cells were collected and washed twice with sterile ddH_2_O before extracting total RNA using RNeasy^®^ Mini Kit (Qiagen, Hilden, Germany) according to the manufacturer’s instructions. Three biologically independent replicates were applied to minimize errors.

Qualified total RNA was clustered by TruSeq PE Cluster Kit v3-cBot-HS (Illumia), and sequenced on Illumina Novaseq platform (Novogene Bioinformatics Technology Co. Ltd). Software Hisat2, FeatureCounts v1.5.0-p3, and DESeq2 R package (1.20.0) were used for mapping, reads counting, and differential expression analysis, respectively. To identify fatty acid responsive promoters, two group comparisons, CEN.PK 113-11C vs CEN.PK 113-11C in oleate, and CEN.PK 113-11C vs YJZ121, were adopted, among which top 50 DEGs in all three sampling points were included, and the final 10 candidate promoters were selected from the intersection of these two groups (Additional file 1: Table S1). Similarly, 10 candidate promoters that responded fatty acyl-CoA were derived from two group comparisons, CEN.PK 113-11C vs JV03, and JV03 vs JV03 in oleate (Additional file 1: Table S2).

### Characterization of fatty acid/acyl-CoA responsive promoters

20 candidate promoters in Table S1 and Table S2 were characterized on episomal plasmid using *eGFP* as a reporter. The plasmids were constructed as illustrated in Figure S9. Briefly, the *eGFP* gene was first inserted into an empty plasmid pYX312 by enzymatic digestion with *Hin*d III and *Sal* I and ligation and the strong constitutive promoter P_*UAS-TDH3*_ [47] was adopted as positive control, generating pSc01. Subsequently, pSc01 was digested by *Kpn* I and *Hin*d III, and the promoter fragments were inserted to obtain a series of plasmids pSc02 ~ pSc21.

To characterize fatty acid responsive promoters, plasmids pSc01 ~ pSc21 were transformed into both wild-type strain (CEN.PK-113C) and FFA producing strain (YJZ121). Wild-type derived strains were cultivated in minimal media with or without 0.5% (v/v) oleic acid to evaluate the responses to exogenous fatty acids. Samples were taken to measure OD_600_ and fluorescence intensity, and the promoter activities were represented by the fluorescence intensity per OD_600_. To evaluate the responses to endogenous fatty acids, fluorescence intensity per OD_600_ of each promoter were characterized in wild-type and YJZ121 background strains that cultivated in minimal medium. In particular, to test the effects of supplemented carbon sources on the promoter (P_*UAS-TDH3*_, P_*ADH6*_, P_*OYE3*_, P_*PDR5*_, P_*HSP31*_, P_*AAD6*_) responses to endogenous fatty acids, 20 g/L glucose, or galactose was supplemented to the medium at 16 h. Strains were cultured for 64 h, and samples were taken to measure the fluorescence intensity per OD_600_. Similarly, plasmids pSc01 ~ pSc21 were transformed into acyl-CoA producing strain (JV03) for characterizing acyl-CoA responsive promoters in minimal media using wild-type derived strains as the control.

### Statistics analysis

Statistics analysis is performed in Office Excel Software using two-tailed *t* test method of variance ANOVA hypothesis. Significant differences are marked as n.s. *p* > 0.05, *p < 0.05, **p < 0.01, and ***p < 0.001. All data are presented as mean ± s.e.m. The number of biologically independent samples for each panel is typically three unless otherwise stated in the figure legends.

## Supplementary Information

**Additional file 1: Figure S1**. Flowchart of yeast strain construction for fatty alcohol production. *S. cerevisiae* strain was constructed for fatty alcohol production with either episomal synthetic pathway (plasmids), or integrated synthetic pathway (genome). **Figure S2**. Changes in fluorescence intensity with time of GFP under control of promoter P_*GAL1,10*_**.** **Figure S3**. Fatty alcohol production by fatty acyl-CoA reductase (*FAR*) from different species. **Figure S4**. Promoter characterization that responds to exogenous fatty acid and endogenous acyl-CoA based on transcriptional data. **Figure S5**. Promoter characterization that responds to endogenous fatty acid based on transcriptional data. **Figure S6**. Coordinated cytosolic and peroxisomal biosynthetic pathways promoted fatty alcohol production. **Figure S7**. Fatty acid/acyl-CoA responsive promoters for enhanced production of 3-hydroxypropionic acid (3-HP). **Figure S8**. Expression cassettes for genetic manipulation by CRISPR/Cas9 in this study. **Figure S9**. Sketch map of plasmid construction for screening fatty acid/acyl-CoA responsive promoter. **Table S1**. Promoter candidates that respond to fatty acid. **Table S2**. Promoter candidates that respond to fatty acyl-CoA. **Table S3**. Strains used in this study. **Table S4**. Plasmids used in this study. **Table S5**. Primers used in this study. **Table S6**. Codon optimized genes used in this study.

## Data Availability

All data supporting the findings of this study are included in the paper and its supplementary information.

## Abbreviations

CAR: Carboxylic acid reductase
FAR: Fatty acyl-CoA reductase
FFA: Free fatty acid
DEG: Differentially expressed gene
RPKM: Reads per kilobase million
ROS: Reactive oxygen species
3-HP: 3-Hydroxypropionic acid
