# Supplementary material for: Spatial–temporal regulation of fatty alcohol biosynthesis in yeast
Source: Biotechnol Biofuels Bioprod. 2022 Dec 16;15:141. doi: 10.1186/s13068-022-02242-7 (PMC9758912; doi:10.1186/s13068-022-02242-7)
Supplement: Supplementary file 1 — Additional file 1: Figure S1. Flowchart of yeast strain construction for fatty alcohol production. S. cerevisiae strain was constructed for fatty alcohol production with either episomal synthetic pathway (plasmids), or integrated synthetic pathway (genome). Figure S2. Changes in fluorescence intensity with time of GFP under control of promoter PGAL1,10. Figure S3. Fatty alcohol production by fatty acyl-CoA reductase (FAR) from different species. Figure S4. Promoter characterization that responds to exogenous fatty acid and endogenous acyl-CoA based on transcriptional data. Figure S5. Promoter characterization that responds to endogenous fatty acid based on transcriptional data. Figure S6. Coordinated cytosolic and peroxisomal biosynthetic pathways promoted fatty alcohol production. Figure S7. Fatty acid/acyl-CoA responsive promoters for enhanced production of 3-hydroxypropionic acid (3-HP). Figure S8. Expression cassettes for genetic manipulation by CRISPR/Cas9 in this study. Figure S9. Sketch map of plasmid construction for screening fatty acid/acyl-CoA responsive promoter. Table S1. Promoter candidates that respond to fatty acid. Table S2. Promoter candidates that respond to fatty acyl-CoA. Table S3. Strains used in this study. Table S4. Plasmids used in this study. Table S5. Primers used in this study. Table S6. Codon optimized genes used in this study. [file 13068_2022_2242_MOESM1_ESM.pdf]

Additional information

Spatial-temporal regulation of fatty alcohol biosynthesis in yeast

Ning Gao[1,2,3#], Jiaoqi Gao[1,2,3#], Wei Yu[1,4], Sijia Kong[1,4], Yongjin J. Zhou[1,2,3*]

[1]Division of Biotechnology, Dalian Institute of Chemical Physics, Chinese Academy of Sciences, Dalian 116023, China

[2]CAS Key Laboratory of Separation Science for Analytical Chemistry, Dalian Institute of Chemical Physics, Chinese Academy of Sciences, Dalian 116023, China

[3]Dalian Key Laboratory of Energy Biotechnology, Dalian Institute of Chemical Physics, Chinese Academy of Sciences, Dalian 116023, China

[4]University of Chinese Academy of Sciences, Beijing 100049, China

[#]These authors contributed equally.

*Correspondence to: Yongjin J. Zhou. E-mail: zhouyongjin@dicp.ac.cn

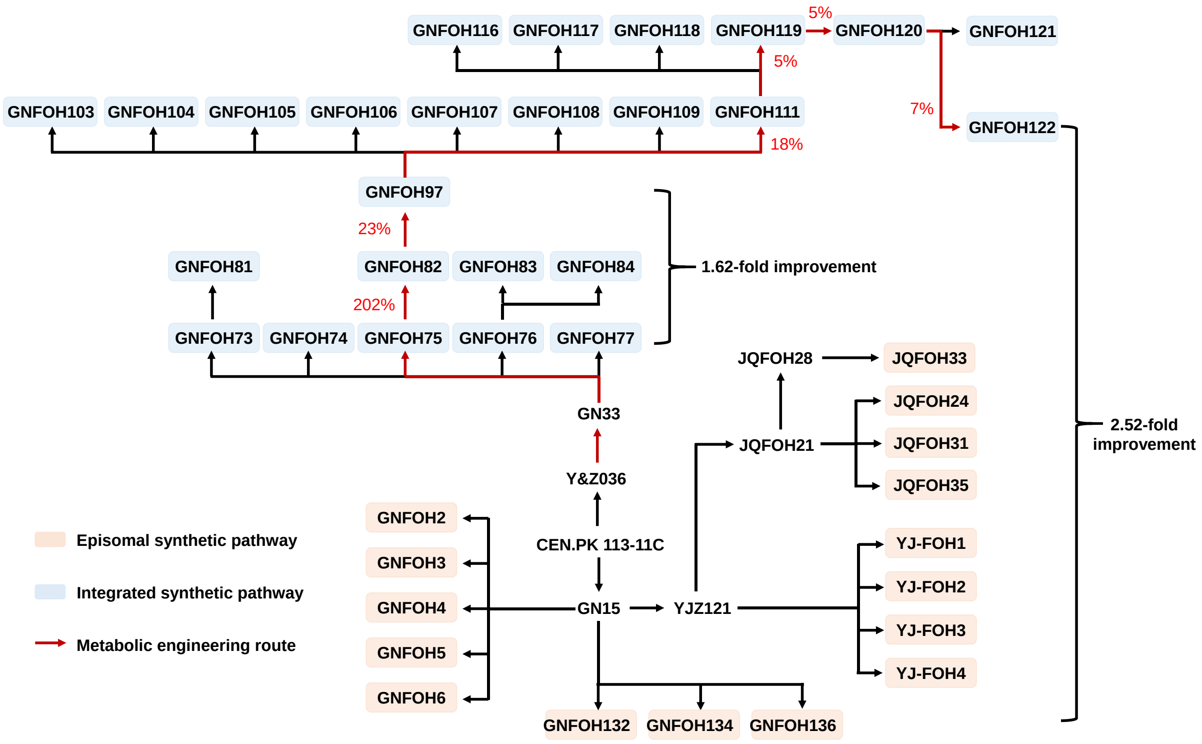

Additional file Figure 1 Flowchart of yeast strain construction for fatty alcohol production. *S. cerevisiae* strain was constructed for fatty alcohol production with either episomal synthetic pathway (plasmids), or integrated synthetic pathway (genome). Numbers in red indicated titer improvement from each metabolic engineering modification. In particular, the fatty acid/acyl-CoA responsive promoters improved fatty alcohol biosynthesis by 1.62-fold (strain GNFFOH73 vs GNFOH97), and strain GNFOH120 with enhanced peroxisomal supply of acyl-CoA and NADPH (+1 mM H_2O_2) had a 2.52-fold higher titer than the starting strain GNFOH136.

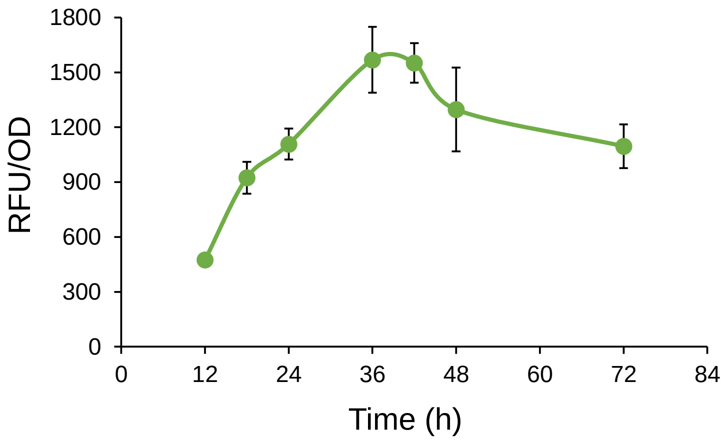

Additional file Figure S2 Changes in fluorescence intensity with time of GFP under control of promoter P*GAL1,10*. Strains were cultivated in minimal medium with 20 g/L glucose at 30°C, 220 rpm for 72 h, and samples were taken to measure the fluorescence intensity. Data are presented as mean ± SD of three biologically independent samples.

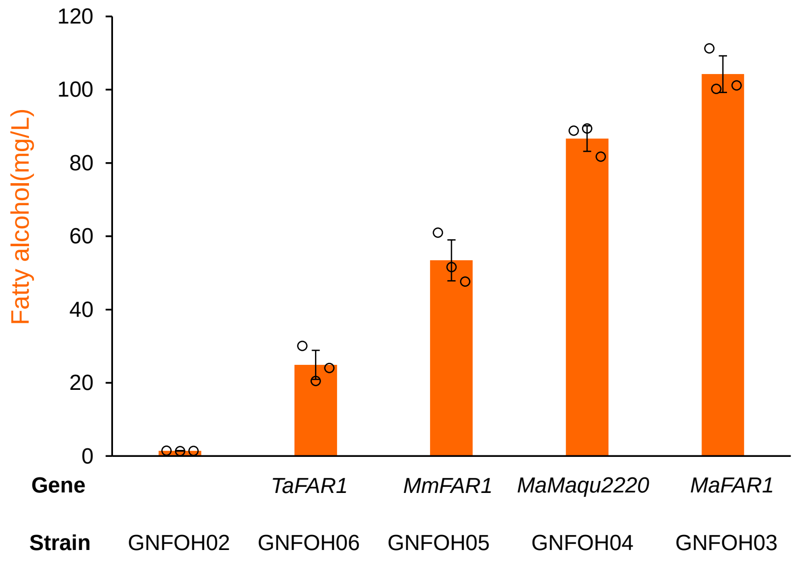

Additional file Figure S3 Fatty alcohol production by fatty acyl-CoA reductase (*FAR*) from different species. *FAR* genes from *Tyto alba (Barn owl)* (*TaFAR1*), *Mus musculus* (*MmFAR1*), *Marinobacter aquaeolei VT8* (*MaMaqu2220*), and *Marinobacter sp. ES-1* (*MaFAR1*) controlled by promoter $P_{GAL1,10}$ were introduced into strain GN15. Engineered strains were cultivated in minimal medium with 20 g/L glucose at 30°C, 220 rpm for 96 h, and fatty alcohol was quantified to evaluate these *FAR* genes. *FAR* genes assembled on plasmid backbone pESC, and Control contained an empty plasmid. Data are presented as mean ± SD of three biologically independent samples with displayed data-points.

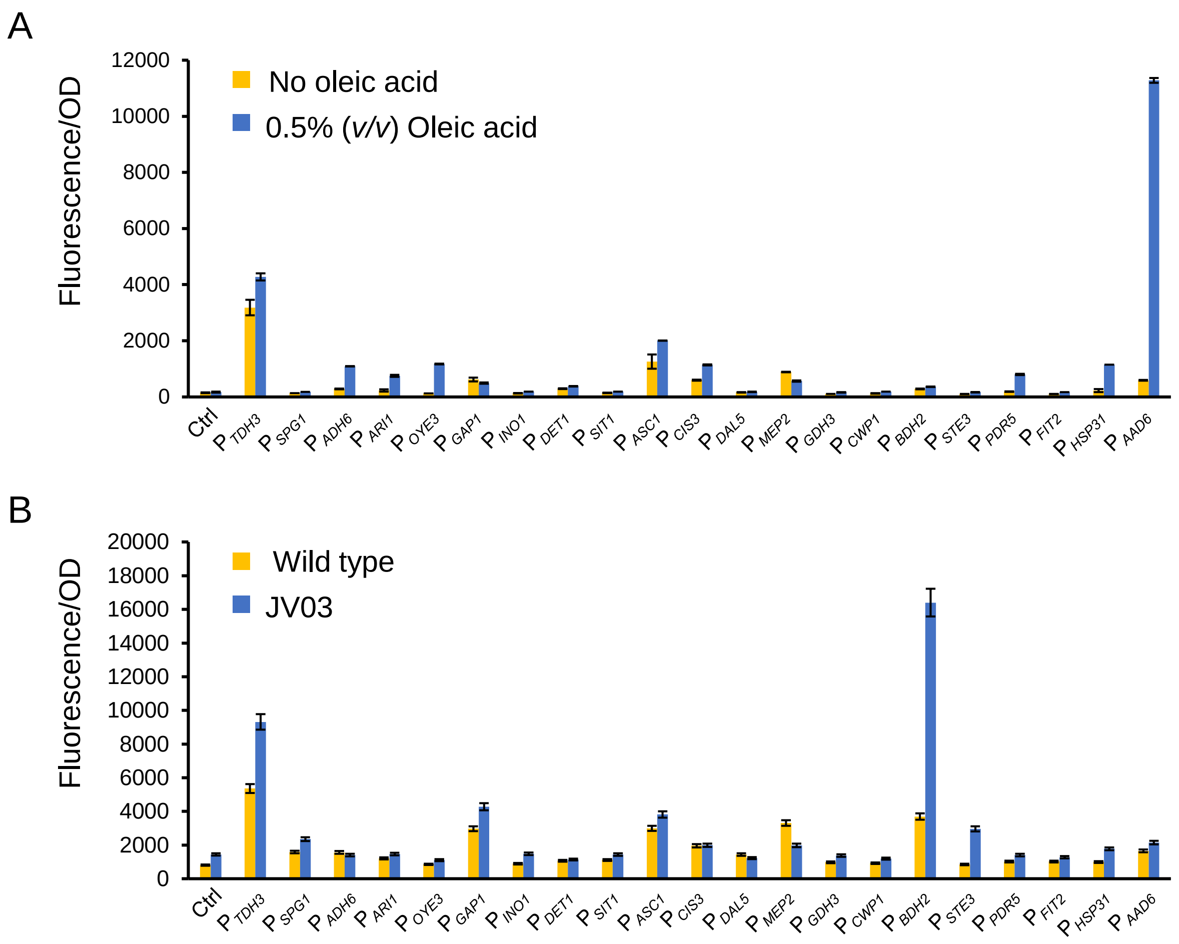

Additional file Figure S4 Promoter characterization that responds to exogenous fatty acid and endogenous acyl-CoA based on transcriptional data. A Promoters that responded to exogenous FFA. GFP under control of diverse promoters was expressed in wild-type, and cultivated in minimal media with, or without 0.5% (*v/v*) oleic acid. **B** Promoters that responded to endogenous acyl-CoA. GFP under control of diverse promoters was expressed in wild-type, and acyl-CoA producing strain JV03 [1], respectively. Data are presented as mean ± SD of three biologically independent samples.

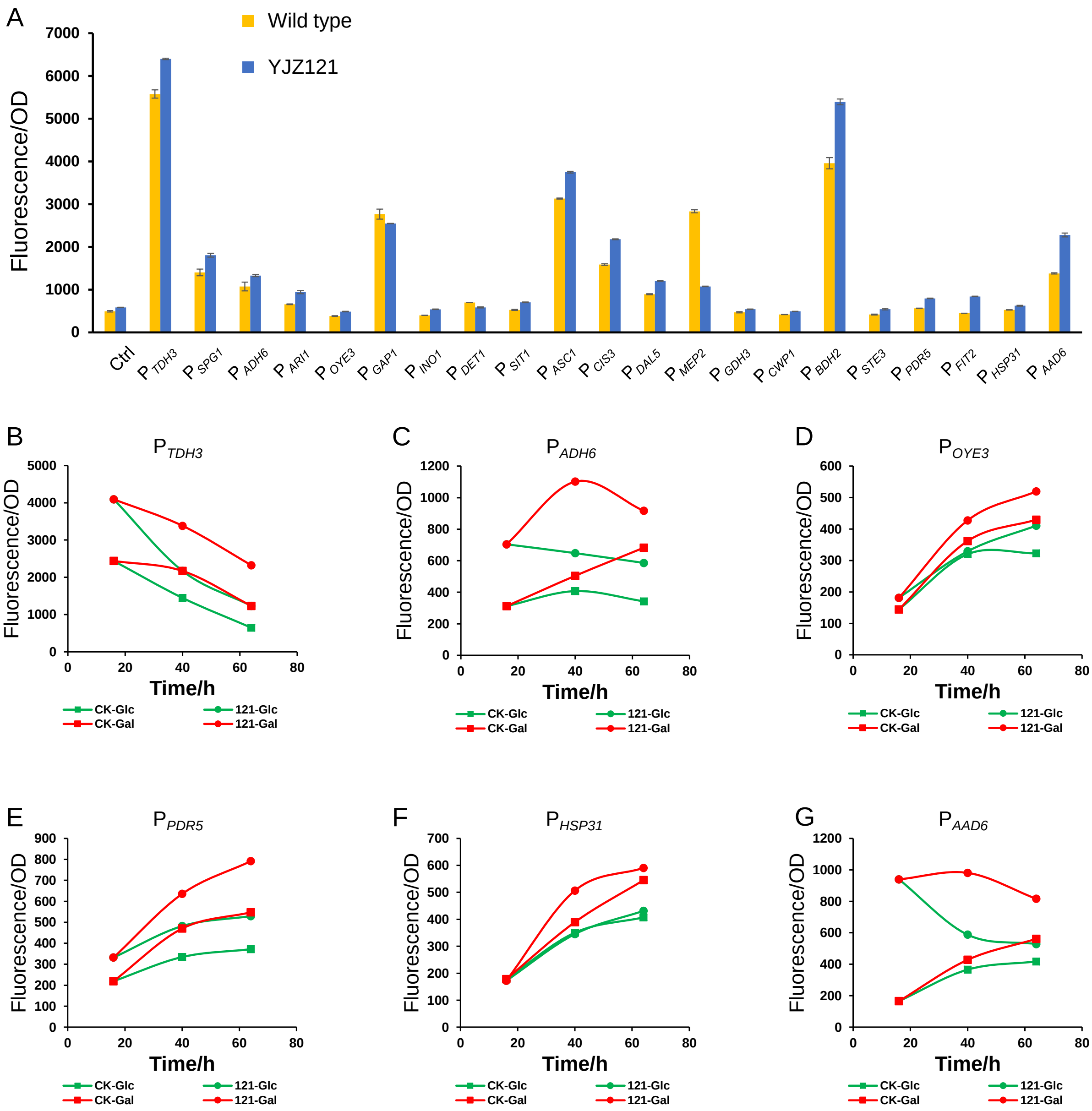

Additional file Figure S5 Promoter characterization that responds to endogenous fatty acid based on transcriptional data. A Promoters that responded to endogenous FFA. GFP under control of diverse promoters was expressed in FFA producing strain. In particular, six promoters, including P_{TDH3} (**B**), P_{ADH6} (**C**), P_{OYE3} (**D**) P_{PDR5} (**E**), P_{HSP31} (**F**), and P_{AAD6} (**G**), was characterized in supplemented carbon sources. Engineered strains were initially cultivated in minimal medium containing 20 g/L, and 20 g/L glucose, or galactose, were supplemented at 16 h to measure the cell growth and fluorescence intensity. Data are presented as mean ± SD of three biologically independent samples.

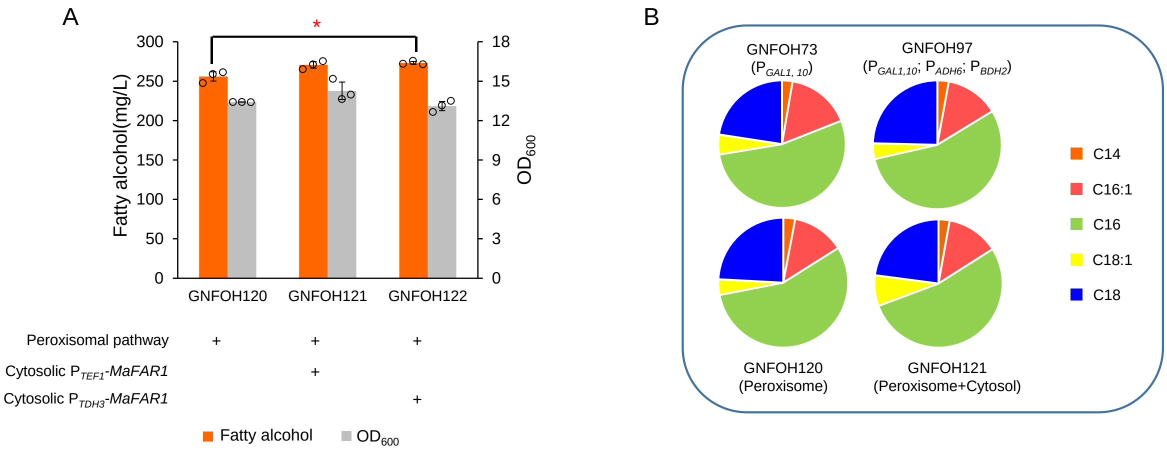

Additional file Figure S6 Coordinated cytosolic and peroxisomal biosynthetic pathways promoted fatty alcohol production. The cytosolic *MaFAR1* was expressed under control of strong constitutive promoters P_{TEF1} and P_{TDH3}, respectively, in strain GNFOH120 with complete peroxisomal biosynthetic pathway of fatty alcohol. **B** The spatio-temporal control strategy via fatty acid/acyl-CoA responsive promoters and cellular compartmentalization did not influence the composition of fatty alcohol, which, however, further utilized the residual cytosolic fatty acid/acyl-CoA in the initial stage of fermentation to slightly achieve higher titer. Data are presented as mean ± SD of three biologically independent samples with displayed data-points. Red asterisks indicate statistical significance as determined using paired t-test (*$P < 0.05$).

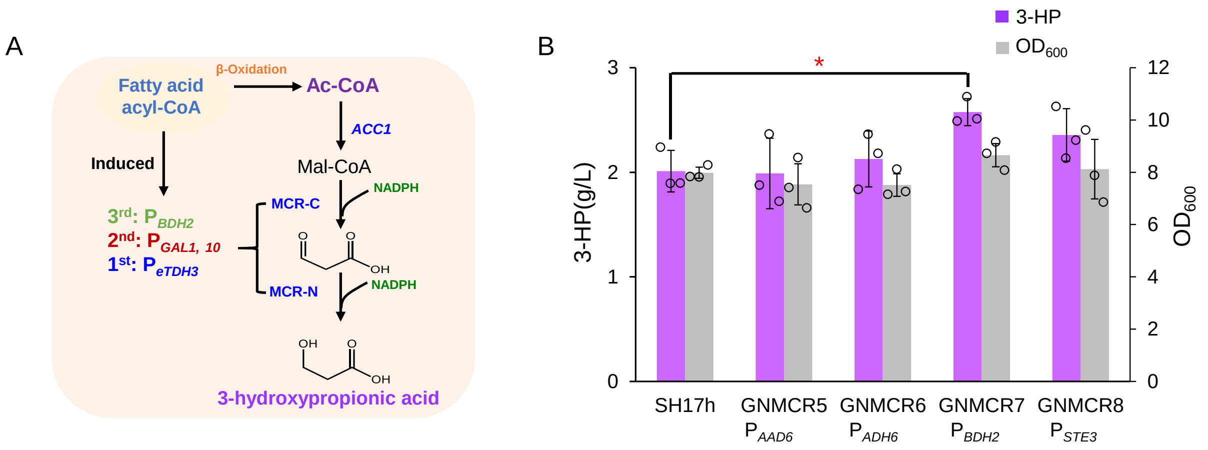

Additional file Figure S7 Fatty acid/acyl-CoA responsive promoters for enhanced 3-hydroxypropionic acid (3-HP) production. A Sketch map of biosynthetic pathway of 3-HP. Biosynthesis of 3-HP and fatty alcohol shares the same precursor acetyl-CoA, which is converted to 3-HP by acetyl-CoA carboxylase (*ACC1*) and malonyl-CoA reductase (*MCR*). Accumulation of fatty acids and acyl-CoA activates both β-oxidation to generate acetyl-CoA, and fatty acid/acyl-CoA responsive promoters to express *MCR*. **B** 3-HP biosynthesis controlled by fatty acid/acyl-CoA responsive promoters. Based on the starting 3-HP producing strain (SH17h) with two copies of *MCR* under the control of P_{TDH3} and $P_{GAL1,\ 10}$, respectively, a third copy of *MCR* controlled by fatty acid/acyl-CoA responsive promoters was introduced. SH17h with high-level production of 3-HP as starting strain, GNMCR5, GNMCR6, GNMCR7, GNMCR8 possessed a third copy of *MCR* with P_{AAD6}, P_{ADH6}, P_{BDH2} and P_{STE3}, respectively. Data are presented as mean ± SD of three biologically independent samples with displayed data-points. Red asterisks indicate statistical significance as determined using paired t-test (*$P < 0.05$).

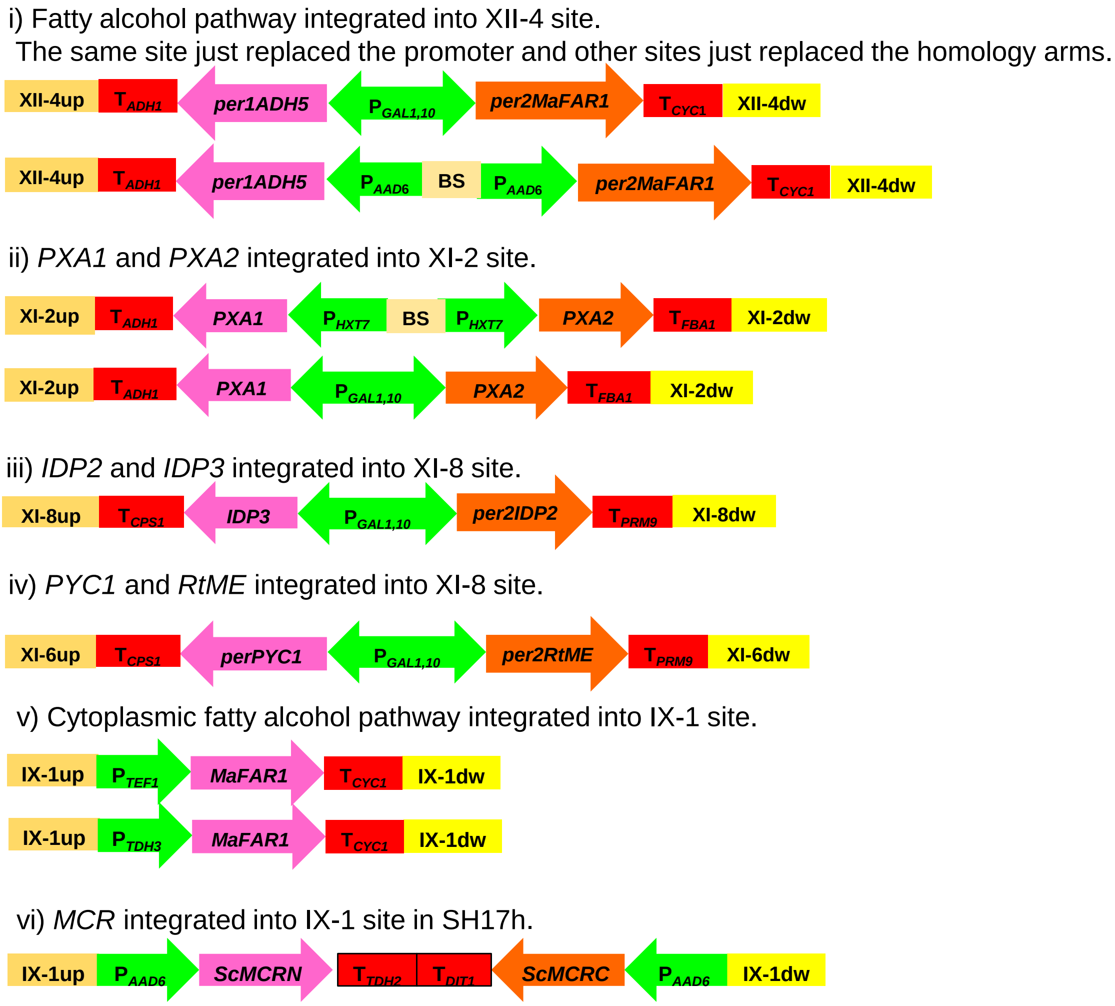

Additional file Figure S8 Expression cassettes for genetic manipulation by CRISPR/Cas9 in this study. Expression cassettes were constructed by OE-PCR to fuse the upstream HA, promoter sequence, open reading frame (ORF), terminator and downstream HA, which was subsequently integrated at different neutral sites. In particular, to avoid the failure in OE-PCR due to the same fragment, a blank sequence (BS) was added. The genes targeted to peroxisome by adding the peroxisomal targeting signal (per1 and per2) [2].

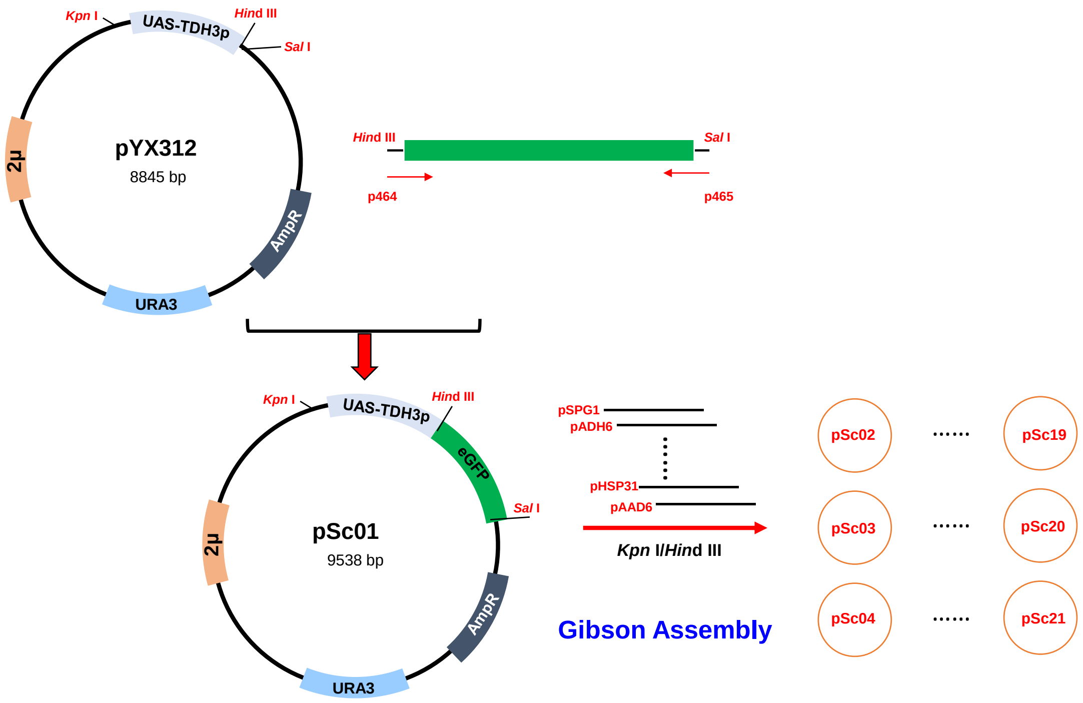

Additional file Figure S9 Sketch map of plasmid construction for screening fatty acid/acyl-CoA responsive promoter. Briefly, the fragment eGFP was first inserted into an empty plasmid pYX312 by enzymatic digestion with *Hin*d III and *Sal* I and ligation, among which strong constitutive promoter $P_{UAS\text{-}TDH3}$ was adopted as positive control, generating pSc01. Subsequently, pSc01 was digested by *Kpn* I and *Hin*d III, and the promoter fragments were inserted to obtain a series of plasmids, pSc02~pSc21.

Table S1 Promoter candidates that respond to fatty acid

Gene	Protein	Maximum RPKM	Fold change
SPG1	Protein required for high temper2ature survival during stationary phase	7500	10
ADH6	NADP-dependent alcohol dehydrogenase	20000	40
ARI1	Carbonyl reductase	1300	32.5
OYE3	NADPH dehydrogenase	20000	35
GAP1	Amino acid per2mease	22000	30
INO1	Inositol-3-phosphate synthase	19000	15
DET1	Acid phosphatase	3300	5.3
SIT1	Siderophore transporter	4800	17
ASC1	Guanine nucleotide-binding protein subunit beta	10000	40
CIS3	Mannose-containing glycoprote	6500	30

Note: promoters were screened from CEN.PK 113-11C vs CEN.PK 113-11C in oleate, and CEN.PK 113-11C vs YJZ121 in Figure 3A

Table S2 Promoter candidates that respond to fatty acyl-CoA

Gene	Protein	Maximum RPKM	Fold change
DAL5	Allantoate per2mease	10260	16
MEP2	Ammonium per2mease	32193	12.5
GDH3	NADP(+)-dependent glutamate dehydrogenase	14000	13
CWP1	Cell wall mannoprotein	6246	6.5
BDH2	Medium-chain alcohol dehydrogenase	19574	16
STE3	Receptor for a factor pheromone	1840	33
PDR5	ATP-binding cassette multidrug transporter	27453	11
FIT2	Mannoprotein	5570	24
HSP31	Glutathione-independent methylglyoxalase	10277	28
AAD6	Aryl-alcohol dehydrogenase	10000	35

Note: promoters were screened from CEN.PK 113-11C vs JV03, and JV03 vs JV03 in oleate in Figure 3A.

Table S3 Strains used in this study

Strains	Genotype	Resource
DH5α	*F-, φ80d/lacZΔM15, Δ(lacZYA-argF)U169, deoR, recA1, endA1, hsdR17(rk-, mk+), phoA, supE44, λ-, thi-1, gyrA96, relA1*	Takara
CEN.PK 113-11C	wild-type, *MATa*; *ura3-52*; *his3Δ1*	Kötter, University of Frankfurt, Germany
GN15	*MATa; MAL2-8c; SUC2; his3Δ1; ura3-52; X1-5::Cas9; gal80Δ; hfd1Δ*	This study
GNFOH2	*MATa; MAL2-8c; SUC2; his3Δ1; ura3-52; X1-5::Cas9; gal80Δ; hfd1Δ;* pESC	This study
GNFOH3	*MATa; MAL2-8c; SUC2; his3Δ1; ura3-52; X1-5::Cas9; gal80Δ; hfd1Δ;* pMaFAR1	This study
GNFOH4	*MATa; MAL2-8c; SUC2; his3Δ1; ura3-52; X1-5::Cas9; gal80Δ; hfd1Δ;* pMaMaqu2220	This study
GNFOH5	*MATa; MAL2-8c; SUC2; his3Δ1; ura3-52; X1-5::Cas9; gal80Δ; hfd1Δ;* pMmFAR1	This study
GNFOH6	*MATa; MAL2-8c; SUC2; his3Δ1; ura3-52; X1-5::Cas9; gal80Δ; hfd1Δ;* pTaFAR1	This study
YJZ121	*MATa; ura3-52; his3Δ1 hfd1Δ; faa1Δ; faa4Δ gal80Δ; XI-5:: (*P$_{TEF1}$-*Cas9*-T$_{CYC1}$*)*	This study
YJ-FOH1	*MATa; ura3-52; his3Δ1 hfd1Δ; faa1Δ; faa4Δ gal80Δ; XI-5::* (P$_{TEF1}$-*Cas9*-T$_{CYC1}$); pYX212-*(*P$_{TPI}$-*npgA*-T$_{FBA1}$*)+(*P$_{TDH3}$-*MmCAR*-T$_{ADH1}$*)+(*P$_{tHXT7}$-*ADH5*-T$_{CYC1}$*)+(* P$_{TEF1}$-*FacoAR*-T$_{pYX212}$*)*	This study
YJ-FOH2	*MATa; ura3-52; his3Δ1 hfd1Δ; faa1Δ; faa4Δ gal80Δ; XI-5::* (P$_{TEF1}$-*Cas9*-T$_{CYC1}$); pYX212-(P$_{TPI}$-*npgAper1*-T$_{FBA1}$)+(P$_{TDH3}$-*MmCARper2*-T$_{ADH1}$)+(P$_{tHXT7}$-*ADH5per1*-T$_{CYC1}$)+(P$_{TEF1}$-*FacoARper2*-T$_{pYX212}$)	This study
YJ-FOH3	*MATa; ura3-52; his3Δ1 hfd1Δ; faa1Δ; faa4Δ gal80Δ; XI-5::* (P$_{TEF1}$-*Cas9*-T$_{CYC1}$); pYX212-(P$_{GAL3}$-*npgA*-T$_{FBA1}$)+(P$_{GAL7}$-*MmCAR*-T$_{ADH1}$)+(P$_{GAL10}$-*ADH5*-T$_{CYC1}$)+(P$_{GAL1}$-*FacoAR*-T$_{pYX212}$)	This study
YJ-FOH4	*MATa; ura3-52; his3Δ1 hfd1Δ; faa1Δ; faa4Δ gal80Δ; XI-5::* (P$_{TEF1}$-*Cas9*-T$_{CYC1}$); pYX212-(P$_{GAL3}$-*npgAper1*-T$_{FBA1}$)+(P$_{GAL7}$-*MmCARper2*-T$_{ADH1}$)+(P$_{GAL10}$-*ADH5per1*-T$_{CYC1}$)+(P$_{GAL1}$-*FacoARper2*-T$_{pYX212}$)	This study
JQFOH21	*MATa; ura3-52; his3Δ1 hfd1Δ; faa1Δ; faa4Δ gal80Δ; XI-5::* (P$_{TEF1}$-*Cas9*-T$_{CYC1}$*); ura3Δ:: (*P$_{HXT6}$-*FATP1*-T$_{ADH1}$*)=YJZ121 ura3Δ:: (*P$_{HXT6}$-*FATP1*-T$_{ADH1}$*)*	This study
JQFOH24	*MATa; ura3-52; his3Δ1 hfd1Δ; faa1Δ; faa4Δ gal80Δ; XI-5::* (P$_{TEF1}$-*Cas9*-T$_{CYC1}$*); ura3Δ:: (*P$_{HXT6}$-*FATP1*-T$_{ADH1}$*);* pAOH22= JQFOH21+ pAOH22	This study
JQFOH28	*MATa; ura3-52; his3Δ1 hfd1Δ; faa1Δ; faa4Δ gal80Δ; XI-5::* (P$_{TEF1}$-*Cas9*-T$_{CYC1}$*); ura3Δ:: (*P$_{HXT6}$-*FATP1*-T$_{ADH1}$*); his3Δ1::HIS3-(*P$_{ADH2}$-*PEX28*-T$_{HIS3}$*)=* JQFOH21; *his3Δ1::HIS3+(*P$_{ADH2}$-*PEX28*-T$_{HIS3}$*)*	This study
JQFOH31	*MATa; ura3-52; his3Δ1 hfd1Δ; faa1Δ; faa4Δ gal80Δ; XI-5::* (P$_{TEF1}$-*Cas9*-T$_{CYC1}$*); ura3Δ:: (*P$_{HXT6}$-*FATP1*-T$_{ADH1}$*); his3Δ1::HIS3-(*P$_{ADH2}$-*PEX7*-T$_{PRM9}$*);* pAOH22= JQFOH21; *his3Δ1::HIS3+(*P$_{ADH2}$-*PEX7*-T$_{PRM9}$*);* pAOH22	This study

JQFOH33	*MATa; ura3-52; his3Δ1 hfd1Δ; faa1Δ; faa4Δ gal80Δ; XI-5:: (*P_{TEF1}*-Cas9-*T_{CYC1}*); ura3Δ:: (*P_{HXT6}*-FATP1-*T_{ADH1}*); his3Δ1::HIS3+(*P_{ADH2}*-PEX28-*T_{HIS3}*);* pAOH22= JQFOH28+pAOH22	This study
JQFOH35	*MATa; ura3-52; his3Δ1 hfd1Δ; faa1Δ; faa4Δ gal80Δ; XI-5:: (*P_{TEF1}*-Cas9-*T_{CYC1}*); ura3Δ:: (*P_{HXT6}*-FATP1-*T_{ADH1}*); his3Δ1::HIS3-(*P_{ADH2}*-PEX7-*T_{PRM9}*)-(*P_{ADH2}*-PEX28-*T_{HIS3}*);* pAOH22= JQFOH24*; his3Δ1::HIS3+(*P_{ADH2}*-PEX7-*T_{PRM9}*)+(*P_{ADH2}*-PEX28-*T_{HIS3}*)*	This study
JV03	*MATa; MAL2-8c; SUC2; ura3-52; HIS3; are1Δ; dga1Δ; are2Δ; lro1Δ; pox1Δ*	Valle-Rodríguez et al., 2014[1]
Y&Z036	*MATa; MAL2-8c; SUC2; hfd1Δ; pox1Δ; faa1Δ; faa4Δ; gal80Δ; gal1Δ; gal7Δ; his3Δ::HIS3+(*P_{TPI}*-MmACL-*T_{FBA1}*)+(*P_{TDH3}*-RtME-*T_{CYC1}*)+(*P_{tHXT7}*-'MDH3-*T_{TDH2}*)+(*P_{PGK1}*-CTP1-*T_{ADH1}*)+(*P_{TEF1}*-'tesA-*T_{HIS3}*); ura3Δ::(*P_{TPI1}*-RtFAS1-*T_{FBA1}*)+ (*P_{TEF1}*-RtFAS2-*T_{CYC1}*)+amdSym; X1-5::*P_{TEF1}*-Cas9-*T_{CYC1}*; acc1::*P_{TEF1}*-ACC1; pyc1::*P_{TEF1}*-PYC1; XI-4:: (*P_{TPI1}*-MPC1)+(*P_{PGK1}*-MPC3-*T_{DIT1}*); X-2::(*P_{GAL1}*-AnACLa-*T_{CYC1}*)+(*P_{GAL10}*-AnACLb-*T_{ADH1}*); gal10Δ::(*P_{TPI1}*-RtCIT1-*T_{FBA1}*)+(*P_{TDH3}*-IDP2-*T_{CYC1}*)+(*P_{TEF1}*-YHM2-*T_{GAL1}*); pgi1Δ::(*P_{COX9}*-PGI1)+(*P_{TDH3}*-GND1-*T_{CYC1}*)+(*P_{tHXT7}*-TKL1-*T_{TDH2}*)+(*P_{PGK1}*-TAL1-*T_{ADH1}*)+(*P_{TEF1}*-ZWF1); idh2Δ::* P_{GSY1}*-IDH2*	Yu et al., 2018[3]
SCX02	*MATa; MAL2-8c; SUC2; hfd1Δ; pox1Δ; faa1Δ; faa4Δ; gal80Δ; gal1Δ; gal7Δ; his3Δ::+(*P_{TPI}*-MmACL-*T_{FBA1}*)+(*P_{TDH3}*-RtME-*T_{CYC1}*)+(*P_{tHXT7}*-'MDH3-*T_{TDH2}*)+(*P_{PGK1}*-CTP1-*T_{ADH1}*); ura3Δ::(*P_{TPI1}*-RtFAS1-*T_{FBA1}*)+ (*P_{TEF1}*-RtFAS2-*T_{CYC1}*)+amdSym; X1-5::*P_{TEF1}*-Cas9-*T_{CYC1}*; acc1::*P_{TEF1}*-ACC1; pyc1::*P_{TEF1}*-PYC1; XI-4:: (*P_{TPI1}*-MPC1)+(*P_{PGK1}*-MPC3-*T_{DIT1}*); X-2::(*P_{GAL1}*-AnACLa-*T_{CYC1}*)+(*P_{GAL10}*-AnACLb-*T_{ADH1}*); gal10Δ::(*P_{TPI1}*-RtCIT1-*T_{FBA1}*)+(*P_{TDH3}*-IDP2-*T_{CYC1}*)+(*P_{TEF1}*-YHM2-*T_{GAL1}*); pgi1Δ::(*P_{COX9}*-PGI1)+(*P_{TDH3}*-GND1-*T_{CYC1}*)+(*P_{tHXT7}*-TKL1-*T_{TDH2}*)+(*P_{PGK1}*-TAL1-*T_{ADH1}*)+(*P_{TEF1}*-ZWF1); idh2Δ::* P_{GSY1}*-IDH2*=Y&Z036+ *(his3Δ; 'tesAΔ)*	Cao et al., 2023[4]
PC04	*MATa; MAL2-8c; SUC2; hfd1Δ; pox1Δ; gal80Δ; gal1Δ; gal7Δ; his3Δ::+(*P_{TPI}*-MmACL-*T_{FBA1}*)+(*P_{TDH3}*-RtME-*T_{CYC1}*)+(*P_{tHXT7}*-'MDH3-*T_{TDH2}*)+(*P_{PGK1}*-CTP1-*T_{ADH1}*); ura3Δ::(*P_{TPI1}*-RtFAS1-*T_{FBA1}*)+ (*P_{TEF1}*-RtFAS2-*T_{CYC1}*)+amdSym; X1-5::*P_{TEF1}*-Cas9-*T_{CYC1}*; acc1::*P_{TEF1}*-ACC1; pyc1::*P_{TEF1}*-PYC1; XI-4:: (*P_{TPI1}*-MPC1)+(*P_{PGK1}*-MPC3-*T_{DIT1}*); X-2::(*P_{GAL1}*-AnACLa-*T_{CYC1}*)+(*P_{GAL10}*-AnACLb-*T_{ADH1}*); gal10Δ::(*P_{TPI1}*-RtCIT1-*T_{FBA1}*)+(*P_{TDH3}*-IDP2-*T_{CYC1}*)+(*P_{TEF1}*-YHM2-*T_{GAL1}*); pgi1Δ::(*P_{COX9}*-PGI1)+(*P_{TDH3}*-GND1-*T_{CYC1}*)+(*P_{tHXT7}*-TKL1-*T_{TDH2}*)+(*P_{PGK1}*-TAL1-*T_{ADH1}*)+(*P_{TEF1}*-ZWF1); idh2Δ::* P_{GSY1}*-IDH2*= SCX02+ *faa1Δ::FAA1; faa4Δ::FAA4;*	This study
PC06	*MATa; MAL2-8c; SUC2; hfd1Δ; gal80Δ; gal1Δ; gal7Δ; his3Δ:: (*P_{TPI}*-MmACL-*T_{FBA1}*)+(*P_{TDH3}*-RtME-*T_{CYC1}*)+(*P_{tHXT7}*-'MDH3-*T_{TDH2}*)+(*P_{PGK1}*-CTP1-*T_{ADH1}*); ura3Δ::(*P_{TPI1}*-RtFAS1-*T_{FBA1}*)+ (*P_{TEF1}*-RtFAS2-*T_{CYC1}*)+amdSym; X1-5::*P_{TEF1}*-Cas9-*T_{CYC1}*; acc1::*P_{TEF1}*-ACC1; pyc1::*P_{TEF1}*-PYC1; XI-4:: (*P_{TPI1}*-MPC1)+(*P_{PGK1}*-MPC3-*T_{DIT1}*); X-2::(*P_{GAL1}*-AnACLa-*T_{CYC1}*)+(*P_{GAL10}*-AnACLb-*T_{ADH1}*); gal10Δ::(*P_{TPI1}*-RtCIT1-*T_{FBA1}*)+(*P_{TDH3}*-IDP2-*T_{CYC1}*)+(*P_{TEF1}*-YHM2-*	This study

	T$_{GAL1}$); pgi1Δ::(P$_{COX9}$-PGI1)+(P$_{TDH3}$-GND1-T$_{CYC1}$)+(P$_{tHXT7}$-TKL1-T$_{TDH2}$)+(P$_{PGK1}$-TAL1-T$_{ADH1}$)+(P$_{TEF1}$-ZWF1); idh2Δ:: P$_{GSY1}$-IDH2 =PC04+ pox1Δ::POX1;	
GN33	MATa; MAL2-8c; SUC2; hfd1Δ; gal80Δ; gal1Δ; gal7Δ; his3Δ::(HIS3-T$_{ENO2}$)+(P$_{ADH2}$-PEX28-T$_{HIS3}$); ura3Δ::(P$_{TPI1}$-RtFAS1-T$_{FBA1}$)+ (P$_{TEF1}$-RtFAS2-T$_{CYC1}$)+amdSym; X1-5::P$_{TEF1}$-Cas9-T$_{CYC1}$; acc1::P$_{TEF1}$-ACC1; pyc1::P$_{TEF1}$-PYC1; XI-4:: (P$_{TPI1}$-MPC1)+(P$_{PGK1}$-MPC3-T$_{DIT1}$); X-2::(P$_{GAL1}$-AnACLa-T$_{CYC1}$)+(P$_{GAL10}$-AnACLb-T$_{ADH1}$); gal10Δ::(P$_{TPI1}$-RtCIT1-T$_{FBA1}$)+(P$_{TDH3}$-IDP2-T$_{CYC1}$)+(P$_{TEF1}$-YHM2-T$_{GAL1}$); pgi1Δ::(P$_{COX9}$-PGI1)+(P$_{TDH3}$-GND1-T$_{CYC1}$)+(P$_{tHXT7}$-TKL1-T$_{TDH2}$)+(P$_{PGK1}$-TAL1-T$_{ADH1}$)+(P$_{TEF1}$-ZWF1); idh2Δ:: P$_{GSY1}$-IDH2	This study
GN33-AAD6	MATa; MAL2-8c; SUC2; hfd1Δ; gal80Δ; gal1Δ; gal7Δ; his3Δ::(HIS3-T$_{ENO2}$)+(P$_{ADH2}$-PEX28-T$_{HIS3}$); ura3Δ::(P$_{TPI1}$-RtFAS1-T$_{FBA1}$)+ (P$_{TEF1}$-RtFAS2-T$_{CYC1}$)+amdSym; X1-5::P$_{TEF1}$-Cas9-T$_{CYC1}$; acc1::P$_{TEF1}$-ACC1; pyc1::P$_{TEF1}$-PYC1; XI-4:: (P$_{TPI1}$-MPC1)+(P$_{PGK1}$-MPC3-T$_{DIT1}$); X-2::(P$_{GAL1}$-AnACLa-T$_{CYC1}$)+(P$_{GAL10}$-AnACLb-T$_{ADH1}$); gal10Δ::(P$_{TPI1}$-RtCIT1-T$_{FBA1}$)+(P$_{TDH3}$-IDP2-T$_{CYC1}$)+(P$_{TEF1}$-YHM2-T$_{GAL1}$); pgi1Δ::(P$_{COX9}$-PGI1)+(P$_{TDH3}$-GND1-T$_{CYC1}$)+(P$_{tHXT7}$-TKL1-T$_{TDH2}$)+(P$_{PGK1}$-TAL1-T$_{ADH1}$)+(P$_{TEF1}$-ZWF1); idh2Δ:: P$_{GSY1}$-IDH2; VII-2:: P$_{AAD6}$-eGFP-T$_{ADH1}$= GN33+VII-2::P$_{AAD6}$-eGFP-T$_{ADH1}$	This study
GN33-ADH6	MATa; MAL2-8c; SUC2; hfd1Δ; gal80Δ; gal1Δ; gal7Δ; his3Δ::(HIS3-T$_{ENO2}$)+(P$_{ADH2}$-PEX28-T$_{HIS3}$); ura3Δ::(P$_{TPI1}$-RtFAS1-T$_{FBA1}$)+ (P$_{TEF1}$-RtFAS2-T$_{CYC1}$)+amdSym; X1-5::P$_{TEF1}$-Cas9-T$_{CYC1}$; acc1::P$_{TEF1}$-ACC1; pyc1::P$_{TEF1}$-PYC1; XI-4:: (P$_{TPI1}$-MPC1)+(P$_{PGK1}$-MPC3-T$_{DIT1}$); X-2::(P$_{GAL1}$-AnACLa-T$_{CYC1}$)+(P$_{GAL10}$-AnACLb-T$_{ADH1}$); gal10Δ::(P$_{TPI1}$-RtCIT1-T$_{FBA1}$)+(P$_{TDH3}$-IDP2-T$_{CYC1}$)+(P$_{TEF1}$-YHM2-T$_{GAL1}$); pgi1Δ::(P$_{COX9}$-PGI1)+(P$_{TDH3}$-GND1-T$_{CYC1}$)+(P$_{tHXT7}$-TKL1-T$_{TDH2}$)+(P$_{PGK1}$-TAL1-T$_{ADH1}$)+(P$_{TEF1}$-ZWF1); idh2Δ:: P$_{GSY1}$-IDH2; VII-2:: P$_{ADH6}$-eGFP-T$_{ADH1}$= GN33+VII-2::P$_{ADH6}$-eGFP-T$_{ADH1}$	This study
GN33-BDH2	MATa; MAL2-8c; SUC2; hfd1Δ; gal80Δ; gal1Δ; gal7Δ; his3Δ::(HIS3-T$_{ENO2}$)+(P$_{ADH2}$-PEX28-T$_{HIS3}$); ura3Δ::(P$_{TPI1}$-RtFAS1-T$_{FBA1}$)+ (P$_{TEF1}$-RtFAS2-T$_{CYC1}$)+amdSym; X1-5::P$_{TEF1}$-Cas9-T$_{CYC1}$; acc1::P$_{TEF1}$-ACC1; pyc1::P$_{TEF1}$-PYC1; XI-4:: (P$_{TPI1}$-MPC1)+(P$_{PGK1}$-MPC3-T$_{DIT1}$); X-2::(P$_{GAL1}$-AnACLa-T$_{CYC1}$)+(P$_{GAL10}$-AnACLb-T$_{ADH1}$); gal10Δ::(P$_{TPI1}$-RtCIT1-T$_{FBA1}$)+(P$_{TDH3}$-IDP2-T$_{CYC1}$)+(P$_{TEF1}$-YHM2-T$_{GAL1}$); pgi1Δ::(P$_{COX9}$-PGI1)+(P$_{TDH3}$-GND1-T$_{CYC1}$)+(P$_{tHXT7}$-TKL1-T$_{TDH2}$)+(P$_{PGK1}$-TAL1-T$_{ADH1}$)+(P$_{TEF1}$-ZWF1); idh2Δ:: P$_{GSY1}$-IDH2; VII-2:: P$_{BDH2}$-eGFP-T$_{ADH1}$= GN33+VII-2::P$_{BDH2}$-eGFP-T$_{ADH1}$	This study
GN33-STE3	MATa; MAL2-8c; SUC2; hfd1Δ; gal80Δ; gal1Δ; gal7Δ; his3Δ::(HIS3-T$_{ENO2}$)+(P$_{ADH2}$-PEX28-T$_{HIS3}$); ura3Δ::(P$_{TPI1}$-RtFAS1-T$_{FBA1}$)+ (P$_{TEF1}$-RtFAS2-T$_{CYC1}$)+amdSym; X1-5::P$_{TEF1}$-Cas9-T$_{CYC1}$; acc1::P$_{TEF1}$-ACC1; pyc1::P$_{TEF1}$-PYC1; XI-4:: (P$_{TPI1}$-MPC1)+(P$_{PGK1}$-MPC3-T$_{DIT1}$); X-2::(P$_{GAL1}$-AnACLa-T$_{CYC1}$)+(P$_{GAL10}$-AnACLb-T$_{ADH1}$); gal10Δ::(P$_{TPI1}$-RtCIT1-T$_{FBA1}$)+(P$_{TDH3}$-IDP2-T$_{CYC1}$)+(P$_{TEF1}$-YHM2-T$_{GAL1}$); pgi1Δ::(P$_{COX9}$-PGI1)+(P$_{TDH3}$-GND1-T$_{CYC1}$)+(P$_{tHXT7}$-TKL1-T$_{TDH2}$)+(P$_{PGK1}$-TAL1-T$_{ADH1}$)+(P$_{TEF1}$-ZWF1); idh2Δ:: P$_{GSY1}$-IDH2; VII-2:: P$_{STE3}$-eGFP-T$_{ADH1}$= GN33+VII-2::P$_{STE3}$-eGFP-T$_{ADH1}$	This study
GN33-	MATa; MAL2-8c; SUC2; hfd1Δ; gal80Δ; gal1Δ; gal7Δ; his3Δ::(HIS3-	This study

GAL1,10	T$_{ENO2}$)+(P$_{ADH2}$-PEX28-T$_{HIS3}$); ura3Δ::(P$_{TPI1}$-RtFAS1-T$_{FBA1}$)+ (P$_{TEF1}$-RtFAS2-T$_{CYC1}$)+amdSym; X1-5::P$_{TEF1}$-Cas9-T$_{CYC1}$; acc1::P$_{TEF1}$-ACC1; pyc1::P$_{TEF1}$-PYC1; XI-4:: (P$_{TPI1}$-MPC1)+(P$_{PGK1}$-MPC3-T$_{DIT1}$); X-2::(P$_{GAL1}$-AnACLa-T$_{CYC1}$)+(P$_{GAL10}$-AnACLb-T$_{ADH1}$); gal10Δ::(P$_{TPI1}$-RtCIT1-T$_{FBA1}$)+(P$_{TDH3}$-IDP2-T$_{CYC1}$)+(P$_{TEF1}$-YHM2-T$_{GAL1}$); pgi1Δ::(P$_{COX9}$-PGI1)+(P$_{TDH3}$-GND1-T$_{CYC1}$)+(P$_{tHXT7}$-TKL1-T$_{TDH2}$)+(P$_{PGK1}$-TAL1-T$_{ADH1}$)+(P$_{TEF1}$-ZWF1); idh2Δ:: P$_{GSY1}$-IDH2; VII-2:: P$_{GAL1,10}$-eGFP-T$_{ADH1}$= GN33+VII-2::P$_{GAL1,10}$-eGFP-T$_{ADH1}$	
GNFOH73	MATa; MAL2-8c; SUC2; hfd1Δ; gal80Δ; gal1Δ; gal7Δ; his3Δ::(HIS3-T$_{ENO2}$)+(P$_{ADH2}$-PEX28-T$_{HIS3}$); ura3Δ::(P$_{TPI1}$-RtFAS1-T$_{FBA1}$)+ (P$_{TEF1}$-RtFAS2-T$_{CYC1}$)+amdSym; X1-5::P$_{TEF1}$-Cas9-T$_{CYC1}$; acc1::P$_{TEF1}$-ACC1; pyc1::P$_{TEF1}$-PYC1; XI-4:: (P$_{TPI1}$-MPC1)+(P$_{PGK1}$-MPC3-T$_{DIT1}$); X-2::(P$_{GAL1}$-AnACLa-T$_{CYC1}$)+(P$_{GAL10}$-AnACLb-T$_{ADH1}$); gal10Δ::(P$_{TPI1}$-RtCIT1-T$_{FBA1}$)+(P$_{TDH3}$-IDP2-T$_{CYC1}$)+(P$_{TEF1}$-YHM2-T$_{GAL1}$); pgi1Δ::(P$_{COX9}$-PGI1)+(P$_{TDH3}$-GND1-T$_{CYC1}$)+(P$_{tHXT7}$-TKL1-T$_{TDH2}$)+(P$_{PGK1}$-TAL1-T$_{ADH1}$)+(P$_{TEF1}$-ZWF1); idh2Δ:: P$_{GSY1}$-IDH2;XII-4::(T$_{ADH1}$-per1ADH5-P$_{GAL1,10}$-per2MaFAR1-T$_{CYC1}$)=GN33+XII-4::(T$_{ADH1}$-per1ADH5-P$_{GAL1,10}$-per2MaFAR1-T$_{CYC1}$)	This study
GNFOH74	MATa; MAL2-8c; SUC2; hfd1Δ; gal80Δ; gal1Δ; gal7Δ; his3Δ::(HIS3-T$_{ENO2}$)+(P$_{ADH2}$-PEX28-T$_{HIS3}$); ura3Δ::(P$_{TPI1}$-RtFAS1-T$_{FBA1}$)+ (P$_{TEF1}$-RtFAS2-T$_{CYC1}$)+amdSym; X1-5::P$_{TEF1}$-Cas9-T$_{CYC1}$; acc1::P$_{TEF1}$-ACC1; pyc1::P$_{TEF1}$-PYC1; XI-4:: (P$_{TPI1}$-MPC1)+(P$_{PGK1}$-MPC3-T$_{DIT1}$); X-2::(P$_{GAL1}$-AnACLa-T$_{CYC1}$)+(P$_{GAL10}$-AnACLb-T$_{ADH1}$); gal10Δ::(P$_{TPI1}$-RtCIT1-T$_{FBA1}$)+(P$_{TDH3}$-IDP2-T$_{CYC1}$)+(P$_{TEF1}$-YHM2-T$_{GAL1}$); pgi1Δ::(P$_{COX9}$-PGI1)+(P$_{TDH3}$-GND1-T$_{CYC1}$)+(P$_{tHXT7}$-TKL1-T$_{TDH2}$)+(P$_{PGK1}$-TAL1-T$_{ADH1}$)+(P$_{TEF1}$-ZWF1); idh2Δ:: P$_{GSY1}$-IDH2;XII-4:: (P$_{AAD6}$-per1ADH5-T$_{ADH1}$)+ (P$_{AAD6}$-per2MaFAR1-T$_{CYC1}$)=GN33+XII-4::(P$_{AAD6}$-per1ADH5-T$_{ADH1}$)+ (P$_{AAD6}$-per2MaFAR1-T$_{CYC1}$)	This study
GNFOH75	MATa; MAL2-8c; SUC2; hfd1Δ; gal80Δ; gal1Δ; gal7Δ; his3Δ::(HIS3-T$_{ENO2}$)+(P$_{ADH2}$-PEX28-T$_{HIS3}$); ura3Δ::(P$_{TPI1}$-RtFAS1-T$_{FBA1}$)+ (P$_{TEF1}$-RtFAS2-T$_{CYC1}$)+amdSym; X1-5::P$_{TEF1}$-Cas9-T$_{CYC1}$; acc1::P$_{TEF1}$-ACC1; pyc1::P$_{TEF1}$-PYC1; XI-4:: (P$_{TPI1}$-MPC1)+(P$_{PGK1}$-MPC3-T$_{DIT1}$); X-2::(P$_{GAL1}$-AnACLa-T$_{CYC1}$)+(P$_{GAL10}$-AnACLb-T$_{ADH1}$); gal10Δ::(P$_{TPI1}$-RtCIT1-T$_{FBA1}$)+(P$_{TDH3}$-IDP2-T$_{CYC1}$)+(P$_{TEF1}$-YHM2-T$_{GAL1}$); pgi1Δ::(P$_{COX9}$-PGI1)+(P$_{TDH3}$-GND1-T$_{CYC1}$)+(P$_{tHXT7}$-TKL1-T$_{TDH2}$)+(P$_{PGK1}$-TAL1-T$_{ADH1}$)+(P$_{TEF1}$-ZWF1); idh2Δ:: P$_{GSY1}$-IDH2;XII-4:: (P$_{ADH6}$-per1ADH5-T$_{ADH1}$)+ (P$_{ADH6}$-per2MaFAR1-T$_{CYC1}$)=GN33+XII-4::(P$_{ADH6}$-per1ADH5-T$_{ADH1}$)+ (P$_{ADH6}$-per2MaFAR1-T$_{CYC1}$)	This study
GNFOH76	MATa; MAL2-8c; SUC2; hfd1Δ; gal80Δ; gal1Δ; gal7Δ; his3Δ::(HIS3-T$_{ENO2}$)+(P$_{ADH2}$-PEX28-T$_{HIS3}$); ura3Δ::(P$_{TPI1}$-RtFAS1-T$_{FBA1}$)+ (P$_{TEF1}$-RtFAS2-T$_{CYC1}$)+amdSym; X1-5::P$_{TEF1}$-Cas9-T$_{CYC1}$; acc1::P$_{TEF1}$-ACC1; pyc1::P$_{TEF1}$-PYC1; XI-4:: (P$_{TPI1}$-MPC1)+(P$_{PGK1}$-MPC3-T$_{DIT1}$); X-2::(P$_{GAL1}$-AnACLa-T$_{CYC1}$)+(P$_{GAL10}$-AnACLb-T$_{ADH1}$); gal10Δ::(P$_{TPI1}$-RtCIT1-T$_{FBA1}$)+(P$_{TDH3}$-IDP2-T$_{CYC1}$)+(P$_{TEF1}$-YHM2-T$_{GAL1}$); pgi1Δ::(P$_{COX9}$-PGI1)+(P$_{TDH3}$-GND1-T$_{CYC1}$)+(P$_{tHXT7}$-TKL1-T$_{TDH2}$)+(P$_{PGK1}$-TAL1-T$_{ADH1}$)+(P$_{TEF1}$-ZWF1); idh2Δ:: P$_{GSY1}$-IDH2;XII-4:: (P$_{BDH2}$-per1ADH5-T$_{ADH1}$)+ (P$_{BDH2}$-per2MaFAR1-T$_{CYC1}$)=GN33+XII-4::(P$_{BDH2}$-per1ADH5-T$_{ADH1}$)+ (P$_{BDH2}$-per2MaFAR1-T$_{CYC1}$)	This study
GNFOH77	MATa; MAL2-8c; SUC2; hfd1Δ; gal80Δ; gal1Δ; gal7Δ; his3Δ::(HIS3-T$_{ENO2}$)+(P$_{ADH2}$-PEX28-T$_{HIS3}$); ura3Δ::(P$_{TPI1}$-RtFAS1-T$_{FBA1}$)+ (P$_{TEF1}$-RtFAS2-T$_{CYC1}$)+amdSym; X1-5::P$_{TEF1}$-Cas9-T$_{CYC1}$; acc1::P$_{TEF1}$-ACC1; pyc1::P$_{TEF1}$-PYC1; XI-4:: (P$_{TPI1}$-MPC1)+(P$_{PGK1}$-MPC3-T$_{DIT1}$); X-2::(P$_{GAL1}$-AnACLa-T$_{CYC1}$)+(P$_{GAL10}$-AnACLb-T$_{ADH1}$); gal10Δ::(P$_{TPI1}$-RtCIT1-T$_{FBA1}$)+(P$_{TDH3}$-IDP2-T$_{CYC1}$)+(P$_{TEF1}$-YHM2-T$_{GAL1}$); pgi1Δ::(P$_{COX9}$-PGI1)+(P$_{TDH3}$-GND1-T$_{CYC1}$)+(P$_{tHXT7}$-TKL1-T$_{TDH2}$)+(P$_{PGK1}$-TAL1-T$_{ADH1}$)+(P$_{TEF1}$-ZWF1); idh2Δ:: P$_{GSY1}$-IDH2;XII-4:: (P$_{STE3}$-per1ADH5-T$_{ADH1}$)+ (P$_{STE3}$-per2MaFAR1-T$_{CYC1}$)= GN33+XII-4::(P$_{STE3}$-per1ADH5-T$_{ADH1}$)+ (P$_{STE3}$-per2MaFAR1-T$_{CYC1}$)	This study

GNFOH81	MATa; MAL2-8c; SUC2; hfd1Δ; gal80Δ; gal1Δ; gal7Δ; his3Δ::(HIS3-T_{ENO2})+(P_{ADH2}-PEX28-T_{HIS3}); ura3Δ::(P_{TPI1}-RtFAS1-T_{FBA1})+ (P_{TEF1}-RtFAS2-T_{CYC1})+amdSym; X1-5::P_{TEF1}-Cas9-T_{CYC1}; acc1::P_{TEF1}-ACC1; pyc1::P_{TEF1}-PYC1; XI-4:: (P_{TPI1}-MPC1)+(P_{PGK1}-MPC3-T_{DIT1}); X-2::(P_{GAL1}-AnACLa-T_{CYC1})+(P_{GAL10}-AnACLb-T_{ADH1}); gal10Δ::(P_{TPI1}-RtCIT1-T_{FBA1})+(P_{TDH3}-IDP2-T_{CYC1})+(P_{TEF1}-YHM2-T_{GAL1}); pgi1Δ::(P_{COX9}-PGI1)+(P_{TDH3}-GND1-T_{CYC1})+(P_{tHXT7}-TKL1-T_{TDH2})+(P_{PGK1}-TAL1-T_{ADH1})+(P_{TEF1}-ZWF1); idh2Δ:: P_{GSY1}-IDH2;XII-4::(T_{ADH1}-per1ADH5-$P_{GAL1,10}$-per2MaFAR1-T_{CYC1});VII-2:: (T_{ADH1}-per1ADH5-$P_{GAL1,10}$-per2MaFAR1-T_{CYC1})=GNFOH73+ VII-2:: (T_{ADH1}-per1ADH5-$P_{GAL1,10}$-per2MaFAR1-T_{CYC1})	This study
GNFOH82	MATa; MAL2-8c; SUC2; hfd1Δ; gal80Δ; gal1Δ; gal7Δ; his3Δ::(HIS3-T_{ENO2})+(P_{ADH2}-PEX28-T_{HIS3}); ura3Δ::(P_{TPI1}-RtFAS1-T_{FBA1})+ (P_{TEF1}-RtFAS2-T_{CYC1})+amdSym; X1-5::P_{TEF1}-Cas9-T_{CYC1}; acc1::P_{TEF1}-ACC1; pyc1::P_{TEF1}-PYC1; XI-4:: (P_{TPI1}-MPC1)+(P_{PGK1}-MPC3-T_{DIT1}); X-2::(P_{GAL1}-AnACLa-T_{CYC1})+(P_{GAL10}-AnACLb-T_{ADH1}); gal10Δ::(P_{TPI1}-RtCIT1-T_{FBA1})+(P_{TDH3}-IDP2-T_{CYC1})+(P_{TEF1}-YHM2-T_{GAL1}); pgi1Δ::(P_{COX9}-PGI1)+(P_{TDH3}-GND1-T_{CYC1})+(P_{tHXT7}-TKL1-T_{TDH2})+(P_{PGK1}-TAL1-T_{ADH1})+(P_{TEF1}-ZWF1); idh2Δ:: P_{GSY1}-IDH2;XII-4:: (P_{ADH6}-per1ADH5-T_{ADH1})+(P_{ADH6}-per2MaFAR1-T_{CYC1}); VII-2:: (T_{ADH1}-per1ADH5-$P_{GAL1,10}$-per2MaFAR1-T_{CYC1}) =GNFOH75+ VII-2:: (T_{ADH1}-per1ADH5-$P_{GAL1,10}$-per2MaFAR1-T_{CYC1})	This study
GNFOH83	MATa; MAL2-8c; SUC2; hfd1Δ; gal80Δ; gal1Δ; gal7Δ; his3Δ::(HIS3-T_{ENO2})+(P_{ADH2}-PEX28-T_{HIS3}); ura3Δ::(P_{TPI1}-RtFAS1-T_{FBA1})+ (P_{TEF1}-RtFAS2-T_{CYC1})+amdSym; X1-5::P_{TEF1}-Cas9-T_{CYC1}; acc1::P_{TEF1}-ACC1; pyc1::P_{TEF1}-PYC1; XI-4:: (P_{TPI1}-MPC1)+(P_{PGK1}-MPC3-T_{DIT1}); X-2::(P_{GAL1}-AnACLa-T_{CYC1})+(P_{GAL10}-AnACLb-T_{ADH1}); gal10Δ::(P_{TPI1}-RtCIT1-T_{FBA1})+(P_{TDH3}-IDP2-T_{CYC1})+(P_{TEF1}-YHM2-T_{GAL1}); pgi1Δ::(P_{COX9}-PGI1)+(P_{TDH3}-GND1-T_{CYC1})+(P_{tHXT7}-TKL1-T_{TDH2})+(P_{PGK1}-TAL1-T_{ADH1})+(P_{TEF1}-ZWF1); idh2Δ:: P_{GSY1}-IDH2;XII-4:: (P_{BDH2}-per1ADH5-T_{ADH1})+ (P_{BDH2}-per2MaFAR1-T_{CYC1}); VII-2:: (T_{ADH1}-per1ADH5-$P_{GAL1,10}$-per2MaFAR1-T_{CYC1}) =GNFOH76+ VII-2:: (T_{ADH1}-per1ADH5-$P_{GAL1,10}$-per2MaFAR1-T_{CYC1})	This study
GNFOH84	MATa; MAL2-8c; SUC2; hfd1Δ; gal80Δ; gal1Δ; gal7Δ; his3Δ::(HIS3-T_{ENO2})+(P_{ADH2}-PEX28-T_{HIS3}); ura3Δ::(P_{TPI1}-RtFAS1-T_{FBA1})+ (P_{TEF1}-RtFAS2-T_{CYC1})+amdSym; X1-5::P_{TEF1}-Cas9-T_{CYC1}; acc1::P_{TEF1}-ACC1; pyc1::P_{TEF1}-PYC1; XI-4:: (P_{TPI1}-MPC1)+(P_{PGK1}-MPC3-T_{DIT1}); X-2::(P_{GAL1}-AnACLa-T_{CYC1})+(P_{GAL10}-AnACLb-T_{ADH1}); gal10Δ::(P_{TPI1}-RtCIT1-T_{FBA1})+(P_{TDH3}-IDP2-T_{CYC1})+(P_{TEF1}-YHM2-T_{GAL1}); pgi1Δ::(P_{COX9}-PGI1)+(P_{TDH3}-GND1-T_{CYC1})+(P_{tHXT7}-TKL1-T_{TDH2})+(P_{PGK1}-TAL1-T_{ADH1})+(P_{TEF1}-ZWF1); idh2Δ::P_{GSY1}-IDH2;XII-4:: (P_{BDH2}-per1ADH5-T_{ADH1})+(P_{BDH2}-per2MaFAR1-T_{CYC1}); VII-2::(P_{ADH6}-per1ADH5-T_{ADH1})+ (P_{ADH6}-per2MaFAR1-T_{CYC1})= GNFOH76+ VII-2::(P_{ADH6}-per1ADH5-T_{ADH1})+ (P_{ADH6}-per2MaFAR1-T_{CYC1})	This study
GNFOH97	MATa; MAL2-8c; SUC2; hfd1Δ; gal80Δ; gal1Δ; gal7Δ; his3Δ::(HIS3-T_{ENO2})+(P_{ADH2}-PEX28-T_{HIS3}); ura3Δ::(P_{TPI1}-RtFAS1-T_{FBA1})+ (P_{TEF1}-RtFAS2-T_{CYC1})+amdSym; X1-5::P_{TEF1}-Cas9-T_{CYC1}; acc1::P_{TEF1}-ACC1; pyc1::P_{TEF1}-PYC1; XI-4:: (P_{TPI1}-MPC1)+(P_{PGK1}-MPC3-T_{DIT1}); X-2::(P_{GAL1}-AnACLa-T_{CYC1})+(P_{GAL10}-AnACLb-T_{ADH1}); gal10Δ::(P_{TPI1}-RtCIT1-T_{FBA1})+(P_{TDH3}-IDP2-T_{CYC1})+(P_{TEF1}-YHM2-T_{GAL1}); pgi1Δ::(P_{COX9}-PGI1)+(P_{TDH3}-GND1-T_{CYC1})+(P_{tHXT7}-TKL1-T_{TDH2})+(P_{PGK1}-TAL1-T_{ADH1})+(P_{TEF1}-ZWF1); idh2Δ::P_{GSY1}-IDH2;XII-4:: (P_{ADH6}-per1ADH5-T_{ADH1})+(P_{ADH6}-per2MaFAR1-	This study

	T$_{CYC1}$); VII-2:: (T$_{ADH1}$-per1ADH5-P$_{GAL1,10}$-per2MaFAR1-T$_{CYC1}$);VIII-2::(P$_{BDH2}$-per1ADH5-T$_{ADH1}$)+ (P$_{BDH2}$-per2MaFAR1-T$_{CYC1}$)= GNFOH82+ VIII-2::(P$_{BDH2}$-per1ADH5-T$_{ADH1}$)+ (P$_{BDH2}$-per2MaFAR1-T$_{CYC1}$)	
GNFOH103	MATa; MAL2-8c; SUC2; hfd1Δ; gal80Δ; gal1Δ; gal7Δ; his3Δ::(HIS3-T$_{ENO2}$)+(P$_{ADH2}$-PEX28-T$_{HIS3}$); ura3Δ::(P$_{TPI1}$-RtFAS1-T$_{FBA1}$)+ (P$_{TEF1}$-RtFAS2-T$_{CYC1}$)+amdSym; X1-5::P$_{TEF1}$-Cas9-T$_{CYC1}$; acc1::P$_{TEF1}$-ACC1; pyc1::P$_{TEF1}$-PYC1; XI-4:: (P$_{TPI1}$-MPC1)+(P$_{PGK1}$-MPC3-T$_{DIT1}$); X-2::(P$_{GAL1}$-AnACLa-T$_{CYC1}$)+(P$_{GAL10}$-AnACLb-T$_{ADH1}$); gal10Δ::(P$_{TPI1}$-RtCIT1-T$_{FBA1}$)+(P$_{TDH3}$-IDP2-T$_{CYC1}$)+(P$_{TEF1}$-YHM2-T$_{GAL1}$); pgi1Δ::(P$_{COX9}$-PGI1)+(P$_{TDH3}$-GND1-T$_{CYC1}$)+(P$_{tHXT7}$-TKL1-T$_{TDH2}$)+(P$_{PGK1}$-TAL1-T$_{ADH1}$)+(P$_{TEF1}$-ZWF1); idh2Δ::P$_{GSY1}$-IDH2;XII-4:: (P$_{ADH6}$-per1ADH5-T$_{ADH1}$)+(P$_{ADH6}$-per2MaFAR1-T$_{CYC1}$); VII-2:: (T$_{ADH1}$-per1ADH5-P$_{GAL1,10}$-per2MaFAR1-T$_{CYC1}$);VIII-2::(P$_{BDH2}$-per1ADH5-T$_{ADH1}$)+ (P$_{BDH2}$-per2MaFAR1-T$_{CYC1}$);XI-6::(P$_{GAL1,10}$-per2RtME-T$_{PRM9}$)= GNFOH97+XI-6::(P$_{GAL1,10}$-per2RtME-T$_{PRM9}$)	This study
GNFOH104	MATa; MAL2-8c; SUC2; hfd1Δ; gal80Δ; gal1Δ; gal7Δ; his3Δ::(HIS3-T$_{ENO2}$)+(P$_{ADH2}$-PEX28-T$_{HIS3}$); ura3Δ::(P$_{TPI1}$-RtFAS1-T$_{FBA1}$)+ (P$_{TEF1}$-RtFAS2-T$_{CYC1}$)+amdSym; X1-5::P$_{TEF1}$-Cas9-T$_{CYC1}$; acc1::P$_{TEF1}$-ACC1; pyc1::P$_{TEF1}$-PYC1; XI-4:: (P$_{TPI1}$-MPC1)+(P$_{PGK1}$-MPC3-T$_{DIT1}$); X-2::(P$_{GAL1}$-AnACLa-T$_{CYC1}$)+(P$_{GAL10}$-AnACLb-T$_{ADH1}$); gal10Δ::(P$_{TPI1}$-RtCIT1-T$_{FBA1}$)+(P$_{TDH3}$-IDP2-T$_{CYC1}$)+(P$_{TEF1}$-YHM2-T$_{GAL1}$); pgi1Δ::(P$_{COX9}$-PGI1)+(P$_{TDH3}$-GND1-T$_{CYC1}$)+(P$_{tHXT7}$-TKL1-T$_{TDH2}$)+(P$_{PGK1}$-TAL1-T$_{ADH1}$)+(P$_{TEF1}$-ZWF1); idh2Δ:: P$_{GSY1}$-IDH2;XII-4:: (P$_{ADH6}$-per1ADH5-T$_{ADH1}$)+(P$_{ADH6}$-per2MaFAR1-T$_{CYC1}$); VII-2:: (T$_{ADH1}$-per1ADH5-P$_{GAL1,10}$-per2MaFAR1-T$_{CYC1}$);VIII-2::(P$_{BDH2}$-per1ADH5-T$_{ADH1}$)+(P$_{BDH2}$-per2MaFAR1-T$_{CYC1}$);XI-6::(T$_{CPS1}$-per2PYC1-P$_{GAL1,10}$-per2RtME-T$_{PRM9}$)= GNFOH97+XI-6::(T$_{CPS1}$-per2PYC1-P$_{GAL1,10}$-per2RtME-T$_{PRM9}$)	This study
GNFOH105	MATa; MAL2-8c; SUC2; hfd1Δ; gal80Δ; gal1Δ; gal7Δ; his3Δ::(HIS3-T$_{ENO2}$)+(P$_{ADH2}$-PEX28-T$_{HIS3}$); ura3Δ::(P$_{TPI1}$-RtFAS1-T$_{FBA1}$)+ (P$_{TEF1}$-RtFAS2-T$_{CYC1}$)+amdSym; X1-5::P$_{TEF1}$-Cas9-T$_{CYC1}$; acc1::P$_{TEF1}$-ACC1; pyc1::P$_{TEF1}$-PYC1; XI-4:: (P$_{TPI1}$-MPC1)+(P$_{PGK1}$-MPC3-T$_{DIT1}$); X-2::(P$_{GAL1}$-AnACLa-T$_{CYC1}$)+(P$_{GAL10}$-AnACLb-T$_{ADH1}$); gal10Δ::(P$_{TPI1}$-RtCIT1-T$_{FBA1}$)+(P$_{TDH3}$-IDP2-T$_{CYC1}$)+(P$_{TEF1}$-YHM2-T$_{GAL1}$); pgi1Δ::(P$_{COX9}$-PGI1)+(P$_{TDH3}$-GND1-T$_{CYC1}$)+(P$_{tHXT7}$-TKL1-T$_{TDH2}$)+(P$_{PGK1}$-TAL1-T$_{ADH1}$)+(P$_{TEF1}$-ZWF1); idh2Δ:: P$_{GSY1}$-IDH2;XII-4:: (P$_{ADH6}$-per1ADH5-T$_{ADH1}$)+(P$_{ADH6}$-per2MaFAR1-T$_{CYC1}$); VII-2:: (T$_{ADH1}$-per1ADH5-P$_{GAL1,10}$-per2MaFAR1-T$_{CYC1}$);VIII-2::(P$_{BDH2}$-per1ADH5-T$_{ADH1}$)+(P$_{BDH2}$-per2MaFAR1-T$_{CYC1}$);XI-6::(P$_{GAL7}$-MDH3-T$_{ENO2}$)+(T$_{CPS1}$-PYC1-P$_{GAL1,10}$-RtME-T$_{PRM9}$)=GNFOH97+XI-6::(P$_{GAL7}$-MDH3-T$_{ENO2}$)+ (T$_{CPS1}$-per2PYC1-P$_{GAL1,10}$-per2RtME-T$_{PRM9}$)	This study
GNFOH106	MATa; MAL2-8c; SUC2; hfd1Δ; gal80Δ; gal1Δ; gal7Δ; his3Δ::(HIS3-T$_{ENO2}$)+(P$_{ADH2}$-PEX28-T$_{HIS3}$); ura3Δ::(P$_{TPI1}$-RtFAS1-T$_{FBA1}$)+ (P$_{TEF1}$-RtFAS2-T$_{CYC1}$)+amdSym; X1-5::P$_{TEF1}$-Cas9-T$_{CYC1}$; acc1::P$_{TEF1}$-ACC1; pyc1::P$_{TEF1}$-PYC1; XI-4:: (P$_{TPI1}$-MPC1)+(P$_{PGK1}$-MPC3-T$_{DIT1}$); X-2::(P$_{GAL1}$-AnACLa-T$_{CYC1}$)+(P$_{GAL10}$-AnACLb-T$_{ADH1}$); gal10Δ::(P$_{TPI1}$-RtCIT1-T$_{FBA1}$)+(P$_{TDH3}$-IDP2-	This study

	T$_{CYC1}$)+(P$_{TEF1}$-YHM2-T$_{GAL1}$); pgi1Δ::(P$_{COX9}$-PGI1)+(P$_{TDH3}$-GND1-T$_{CYC1}$)+(P$_{tHXT7}$-TKL1-T$_{TDH2}$)+(P$_{PGK1}$-TAL1-T$_{ADH1}$)+(P$_{TEF1}$-ZWF1); idh2Δ:: P$_{GSY1}$-IDH2;XII-4:: (P$_{ADH6}$-per1ADH5-T$_{ADH1}$)+(P$_{ADH6}$-per2MaFAR1-T$_{CYC1}$); VII-2:: (T$_{ADH1}$-per1ADH5-P$_{GAL1,10}$-per2MaFAR1-T$_{CYC1}$);VIII-2::(P$_{BDH2}$-per1ADH5-T$_{ADH1}$)+ (P$_{BDH2}$-per2MaFAR1-T$_{CYC1}$);XI-8:(P$_{GAL1,10}$-per2IDP2-T$_{PRM9}$)=GNFOH97+XI-8:(P$_{GAL1,10}$-per2IDP2-T$_{PRM9}$)	
GNFOH107	MATa; MAL2-8c; SUC2; hfd1Δ; gal80Δ; gal1Δ; gal7Δ; his3Δ::(HIS3-T$_{ENO2}$)+(P$_{ADH2}$-PEX28-T$_{HIS3}$); ura3Δ::(P$_{TPI1}$-RtFAS1-T$_{FBA1}$)+ (P$_{TEF1}$-RtFAS2-T$_{CYC1}$)+amdSym; X1-5::P$_{TEF1}$-Cas9-T$_{CYC1}$; acc1::P$_{TEF1}$-ACC1; pyc1::P$_{TEF1}$-PYC1; XI-4:: (P$_{TPI1}$-MPC1)+(P$_{PGK1}$-MPC3-T$_{DIT1}$); X-2::(P$_{GAL1}$-AnACLa-T$_{CYC1}$)+(P$_{GAL10}$-AnACLb-T$_{ADH1}$); gal10Δ::(P$_{TPI1}$-RtCIT1-T$_{FBA1}$)+(P$_{TDH3}$-IDP2-T$_{CYC1}$)+(P$_{TEF1}$-YHM2-T$_{GAL1}$); pgi1Δ::(P$_{COX9}$-PGI1)+(P$_{TDH3}$-GND1-T$_{CYC1}$)+(P$_{tHXT7}$-TKL1-T$_{TDH2}$)+(P$_{PGK1}$-TAL1-T$_{ADH1}$)+(P$_{TEF1}$-ZWF1); idh2Δ:: P$_{GSY1}$-IDH2;XII-4:: (P$_{ADH6}$-per1ADH5-T$_{ADH1}$)+(P$_{ADH6}$-per2MaFAR1-T$_{CYC1}$); VII-2:: (T$_{ADH1}$-per1ADH5-P$_{GAL1,10}$-per2MaFAR1-T$_{CYC1}$); VIII-2::(P$_{BDH2}$-per1ADH5-T$_{ADH1}$)+ (P$_{BDH2}$-per2MaFAR1-T$_{CYC1}$);XI-8::(P$_{GAL1,10}$-IDP3-T$_{CPS1}$)=GNFOH97+XI-8::(P$_{GAL1,10}$-IDP3-T$_{CPS1}$)	This study
GNFOH108	MATa; MAL2-8c; SUC2; hfd1Δ; gal80Δ; gal1Δ; gal7Δ; his3Δ::(HIS3-T$_{ENO2}$)+(P$_{ADH2}$-PEX28-T$_{HIS3}$); ura3Δ::(P$_{TPI1}$-RtFAS1-T$_{FBA1}$)+ (P$_{TEF1}$-RtFAS2-T$_{CYC1}$)+amdSym; X1-5::P$_{TEF1}$-Cas9-T$_{CYC1}$; acc1::P$_{TEF1}$-ACC1; pyc1::P$_{TEF1}$-PYC1; XI-4::(P$_{TPI1}$-MPC1)+(P$_{PGK1}$-MPC3-T$_{DIT1}$); X-2::(P$_{GAL1}$-AnACLa-T$_{CYC1}$)+(P$_{GAL10}$-AnACLb-T$_{ADH1}$); gal10Δ::(P$_{TPI1}$-RtCIT1-T$_{FBA1}$)+(P$_{TDH3}$-IDP2-T$_{CYC1}$)+(P$_{TEF1}$-YHM2-T$_{GAL1}$); pgi1Δ::(P$_{COX9}$-PGI1)+(P$_{TDH3}$-GND1-T$_{CYC1}$)+(P$_{tHXT7}$-TKL1-T$_{TDH2}$)+(P$_{PGK1}$-TAL1-T$_{ADH1}$)+(P$_{TEF1}$-ZWF1); idh2Δ:: P$_{GSY1}$-IDH2;XII-4:: (P$_{ADH6}$-per1ADH5-T$_{ADH1}$)+(P$_{ADH6}$-per2MaFAR1-T$_{CYC1}$); VII-2:: (T$_{ADH1}$-per1ADH5-P$_{GAL1,10}$-per2MaFAR1-T$_{CYC1}$); VIII-2::(P$_{BDH2}$-per1ADH5-T$_{ADH1}$)+ (P$_{BDH2}$-per2MaFAR1-T$_{CYC1}$);XI-8::(T$_{CPS1}$-IDP3-P$_{GAL1,10}$-per2IDP2-T$_{PRM9}$)=GNFOH97+XI-8::(T$_{CPS1}$-IDP3-P$_{GAL1,10}$-per2IDP2-T$_{PRM9}$)	This study
GNFOH109	MATa; MAL2-8c; SUC2; hfd1Δ; gal80Δ; gal1Δ; gal7Δ; his3Δ::(HIS3-T$_{ENO2}$)+(P$_{ADH2}$-PEX28-T$_{HIS3}$); ura3Δ::(P$_{TPI1}$-RtFAS1-T$_{FBA1}$)+ (P$_{TEF1}$-RtFAS2-T$_{CYC1}$)+amdSym; X1-5::P$_{TEF1}$-Cas9-T$_{CYC1}$; acc1::P$_{TEF1}$-ACC1; pyc1::P$_{TEF1}$-PYC1; XI-4::(P$_{TPI1}$-MPC1)+(P$_{PGK1}$-MPC3-T$_{DIT1}$); X-2::(P$_{GAL1}$-AnACLa-T$_{CYC1}$)+(P$_{GAL10}$-AnACLb-T$_{ADH1}$); gal10Δ::(P$_{TPI1}$-RtCIT1-T$_{FBA1}$)+(P$_{TDH3}$-IDP2-T$_{CYC1}$)+(P$_{TEF1}$-YHM2-T$_{GAL1}$); pgi1Δ::(P$_{COX9}$-PGI1)+(P$_{TDH3}$-GND1-T$_{CYC1}$)+(P$_{tHXT7}$-TKL1-T$_{TDH2}$)+(P$_{PGK1}$-TAL1-T$_{ADH1}$)+(P$_{TEF1}$-ZWF1); idh2Δ:: P$_{GSY1}$-IDH2;XII-4:: (P$_{ADH6}$-per1ADH5-T$_{ADH1}$)+ (P$_{ADH6}$-per2MaFAR1-T$_{CYC1}$); VII-2:: (T$_{ADH1}$-per1ADH5-P$_{GAL1,10}$-per2MaFAR1-T$_{CYC1}$);VIII-2::(P$_{BDH2}$-per1ADH5-T$_{ADH1}$)+(P$_{BDH2}$-per2MaFAR1-T$_{CYC1}$); XI-3::(P$_{GAL2-}$FAA2-T$_{ADH1}$)=GNFOH97+XI-3::(P$_{GAL2-}$FAA2-T$_{ADH1}$)	This study
GNFOH111	MATa; MAL2-8c; SUC2; hfd1Δ; gal80Δ; gal1Δ; gal7Δ; his3Δ::(HIS3-T$_{ENO2}$)+(P$_{ADH2}$-PEX28-T$_{HIS3}$); ura3Δ::(P$_{TPI1}$-RtFAS1-T$_{FBA1}$)+ (P$_{TEF1}$-RtFAS2-T$_{CYC1}$)+amdSym; X1-5::P$_{TEF1}$-Cas9-T$_{CYC1}$; acc1::P$_{TEF1}$-ACC1; pyc1::P$_{TEF1}$-	This study

	PYC1; XI-4::(P$_{TPI1}$-MPC1)+(P$_{PGK1}$-MPC3-T$_{DIT1}$); X-2::(P$_{GAL1}$-AnACLa-T$_{CYC1}$)+(P$_{GAL10}$-AnACLb-T$_{ADH1}$); gal10Δ::(P$_{TPI1}$-RtCIT1-T$_{FBA1}$)+(P$_{TDH3}$-IDP2-T$_{CYC1}$)+(P$_{TEF1}$-YHM2-T$_{GAL1}$); pgi1Δ::(P$_{COX9}$-PGI1)+(P$_{TDH3}$-GND1-T$_{CYC1}$)+(P$_{tHXT7}$-TKL1-T$_{TDH2}$)+(P$_{PGK1}$-TAL1-T$_{ADH1}$)+(P$_{TEF1}$-ZWF1); idh2Δ::P$_{GSY1}$-IDH2;XII-4:: (P$_{ADH6}$-per1ADH5-T$_{ADH1}$)+(P$_{ADH6}$-per2MaFAR1-T$_{CYC1}$); VII-2:: (T$_{ADH1}$-per1ADH5-P$_{GAL1,10}$-per2MaFAR1-T$_{CYC1}$);VIII-2::(P$_{BDH2}$-per1ADH5-T$_{ADH1}$)+ (P$_{BDH2}$-per2MaFAR1-T$_{CYC1}$); XI-2::(P$_{HXT7}$-PXA1-T$_{ADH1}$)+(P$_{HXT7}$-PXA2-T$_{FBA1}$)=GNFOH97+XI-2::(P$_{HXT7}$-PXA1-T$_{ADH1}$)+ (P$_{HXT7}$-PXA2-T$_{FBA1}$)	
GNFOH116	MATa; MAL2-8c; SUC2; hfd1Δ; gal80Δ; gal1Δ; gal7Δ; his3Δ::(HIS3-T$_{ENO2}$)+(P$_{ADH2}$-PEX28-T$_{HIS3}$); ura3Δ::(P$_{TPI1}$-RtFAS1-T$_{FBA1}$)+ (P$_{TEF1}$-RtFAS2-T$_{CYC1}$)+amdSym; X1-5::P$_{TEF1}$-Cas9-T$_{CYC1}$; acc1::P$_{TEF1}$-ACC1; pyc1::P$_{TEF1}$-PYC1; XI-4::(P$_{TPI1}$-MPC1)+(P$_{PGK1}$-MPC3-T$_{DIT1}$); X-2::(P$_{GAL1}$-AnACLa-T$_{CYC1}$)+(P$_{GAL10}$-AnACLb-T$_{ADH1}$); gal10Δ::(P$_{TPI1}$-RtCIT1-T$_{FBA1}$)+(P$_{TDH3}$-IDP2-T$_{CYC1}$)+(P$_{TEF1}$-YHM2-T$_{GAL1}$); pgi1Δ::(P$_{COX9}$-PGI1)+(P$_{TDH3}$-GND1-T$_{CYC1}$)+(P$_{tHXT7}$-TKL1-T$_{TDH2}$)+(P$_{PGK1}$-TAL1-T$_{ADH1}$)+(P$_{TEF1}$-ZWF1); idh2Δ::P$_{GSY1}$-IDH2;XII-4:: (P$_{ADH6}$-per1ADH5-T$_{ADH1}$)+(P$_{ADH6}$-per2MaFAR1-T$_{CYC1}$); VII-2:: (T$_{ADH1}$-per1ADH5-P$_{GAL1,10}$-per2MaFAR1-T$_{CYC1}$);VIII-2::(P$_{BDH2}$-per1ADH5-T$_{ADH1}$)+ (P$_{BDH2}$-per2MaFAR1-T$_{CYC1}$); XI-2::(P$_{HXT7}$-PXA1-T$_{ADH1}$)+(P$_{HXT7}$-PXA2-T$_{FBA1}$); XI-3::(P$_{GAL2}$-FAA2-T$_{ADH1}$)=GNFOH111+ XI-3::(P$_{GAL2}$-FAA2-T$_{ADH1}$)	This study
GNFOH117	MATa; MAL2-8c; SUC2; hfd1Δ; gal80Δ; gal1Δ; gal7Δ; his3Δ::(HIS3-T$_{ENO2}$)+(P$_{ADH2}$-PEX28-T$_{HIS3}$); ura3Δ::(P$_{TPI1}$-RtFAS1-T$_{FBA1}$)+ (P$_{TEF1}$-RtFAS2-T$_{CYC1}$)+amdSym; X1-5::P$_{TEF1}$-Cas9-T$_{CYC1}$; acc1::P$_{TEF1}$-ACC1; pyc1::P$_{TEF1}$-PYC1; XI-4::(P$_{TPI1}$-MPC1)+(P$_{PGK1}$-MPC3-T$_{DIT1}$); X-2::(P$_{GAL1}$-AnACLa-T$_{CYC1}$)+(P$_{GAL10}$-AnACLb-T$_{ADH1}$); gal10Δ::(P$_{TPI1}$-RtCIT1-T$_{FBA1}$)+(P$_{TDH3}$-IDP2-T$_{CYC1}$)+(P$_{TEF1}$-YHM2-T$_{GAL1}$); pgi1Δ::(P$_{COX9}$-PGI1)+(P$_{TDH3}$-GND1-T$_{CYC1}$)+(P$_{tHXT7}$-TKL1-T$_{TDH2}$)+(P$_{PGK1}$-TAL1-T$_{ADH1}$)+(P$_{TEF1}$-ZWF1); idh2Δ::P$_{GSY1}$-IDH2;XII-4:: (P$_{ADH6}$-per1ADH5-T$_{ADH1}$)+(P$_{ADH6}$-per2MaFAR1-T$_{CYC1}$); VII-2:: (T$_{ADH1}$-per1ADH5-P$_{GAL1,10}$-per2MaFAR1-T$_{CYC1}$);VIII-2::(P$_{BDH2}$-per1ADH5-T$_{ADH1}$)+ (P$_{BDH2}$-per2MaFAR1-T$_{CYC1}$); XI-2::(P$_{HXT7}$-PXA1-T$_{ADH1}$)+(P$_{HXT7}$-PXA2-T$_{FBA1}$); XI-8::(T$_{CPS1}$-IDP3-P$_{GAL1,10}$-per2IDP2-T$_{PRM9}$) =GNFOH111+ XI-8::(T$_{CPS1}$-IDP3-P$_{GAL1,10}$-per2IDP2-T$_{PRM9}$)	This study
GNFOH118	MATa; MAL2-8c; SUC2; hfd1Δ; gal80Δ; gal1Δ; gal7Δ; his3Δ::(HIS3-T$_{ENO2}$)+(P$_{ADH2}$-PEX28-T$_{HIS3}$); ura3Δ::(P$_{TPI1}$-RtFAS1-T$_{FBA1}$)+ (P$_{TEF1}$-RtFAS2-T$_{CYC1}$)+amdSym; X1-5::P$_{TEF1}$-Cas9-T$_{CYC1}$; acc1::P$_{TEF1}$-ACC1; pyc1::P$_{TEF1}$-PYC1; XI-4::(P$_{TPI1}$-MPC1)+(P$_{PGK1}$-MPC3-T$_{DIT1}$); X-2::(P$_{GAL1}$-AnACLa-T$_{CYC1}$)+(P$_{GAL10}$-AnACLb-T$_{ADH1}$); gal10Δ::(P$_{TPI1}$-RtCIT1-T$_{FBA1}$)+(P$_{TDH3}$-IDP2-T$_{CYC1}$)+(P$_{TEF1}$-YHM2-T$_{GAL1}$); pgi1Δ::(P$_{COX9}$-PGI1)+(P$_{TDH3}$-GND1-T$_{CYC1}$)+(P$_{tHXT7}$-TKL1-T$_{TDH2}$)+(P$_{PGK1}$-TAL1-T$_{ADH1}$)+(P$_{TEF1}$-ZWF1); idh2Δ::P$_{GSY1}$-IDH2;XII-4:: (P$_{ADH6}$-per1ADH5-T$_{ADH1}$)+(P$_{ADH6}$-per2MaFAR1-T$_{CYC1}$); VII-2:: (T$_{ADH1}$-per1ADH5-P$_{GAL1,10}$-per2MaFAR1-T$_{CYC1}$);VIII-2::(P$_{BDH2}$-per1ADH5-T$_{ADH1}$)+ (P$_{BDH2}$-per2MaFAR1-T$_{CYC1}$); XI-2::(P$_{HXT7}$-PXA1-	This study

	T$_{ADH1}$)+(P$_{HXT7}$-PXA2-T$_{FBA1}$);XI-6::(P$_{GAL1,10}$-per2RtME-T$_{PRM9}$)= GNFOH111+XI-6::(P$_{GAL1,10}$-per2RtME-T$_{PRM9}$)	
GNFOH119	MATa; MAL2-8c; SUC2; hfd1Δ; gal80Δ; gal1Δ; gal7Δ; his3Δ::(HIS3-T$_{ENO2}$)+(P$_{ADH2}$-PEX28-T$_{HIS3}$); ura3Δ::(P$_{TPI1}$-RtFAS1-T$_{FBA1}$)+ (P$_{TEF1}$-RtFAS2-T$_{CYC1}$)+amdSym; X1-5::P$_{TEF1}$-Cas9-T$_{CYC1}$; acc1::P$_{TEF1}$-ACC1; pyc1::P$_{TEF1}$-PYC1; XI-4::(P$_{TPI1}$-MPC1)+(P$_{PGK1}$-MPC3-T$_{DIT1}$); X-2::(P$_{GAL1}$-AnACLa-T$_{CYC1}$)+(P$_{GAL10}$-AnACLb-T$_{ADH1}$); gal10Δ::(P$_{TPI1}$-RtCIT1-T$_{FBA1}$)+(P$_{TDH3}$-IDP2-T$_{CYC1}$)+(P$_{TEF1}$-YHM2-T$_{GAL1}$); pgi1Δ::(P$_{COX9}$-PGI1)+(P$_{TDH3}$-GND1-T$_{CYC1}$)+(P$_{tHXT7}$-TKL1-T$_{TDH2}$)+(P$_{PGK1}$-TAL1-T$_{ADH1}$)+(P$_{TEF1}$-ZWF1); idh2Δ:: P$_{GSY1}$-IDH2;XII-4:: (P$_{ADH6}$-per1ADH5-T$_{ADH1}$)+(P$_{ADH6}$-per2MaFAR1-T$_{CYC1}$); VII-2:: (T$_{ADH1}$-per1ADH5-P$_{GAL1,10}$-per2MaFAR1-T$_{CYC1}$);VIII-2::(P$_{BDH2}$-per1ADH5-T$_{ADH1}$)+ (P$_{BDH2}$-per2MaFAR1-T$_{CYC1}$); XI-2::(P$_{HXT7}$-PXA1-T$_{ADH1}$)+(P$_{HXT7}$-PXA2-T$_{FBA1}$); XI-6::(T$_{CPS1}$-per2PYC1-P$_{GAL1,10}$-per2RtME-T$_{PRM9}$)=GNFOH111+XI-6::(T$_{CPS1}$-per2PYC1-P$_{GAL1,10}$-per2RtME-T$_{PRM9}$)	This study
GNFOH120	MATa; MAL2-8c; SUC2; hfd1Δ; gal80Δ; gal1Δ; gal7Δ; his3Δ::(HIS3-T$_{ENO2}$)+(P$_{ADH2}$-PEX28-T$_{HIS3}$); ura3Δ::(P$_{TPI1}$-RtFAS1-T$_{FBA1}$)+ (P$_{TEF1}$-RtFAS2-T$_{CYC1}$)+amdSym; X1-5::P$_{TEF1}$-Cas9-T$_{CYC1}$; acc1::P$_{TEF1}$-ACC1; pyc1::P$_{TEF1}$-PYC1; XI-4::(P$_{TPI1}$-MPC1)+(P$_{PGK1}$-MPC3-T$_{DIT1}$); X-2::(P$_{GAL1}$-AnACLa-T$_{CYC1}$)+(P$_{GAL10}$-AnACLb-T$_{ADH1}$); gal10Δ::(P$_{TPI1}$-RtCIT1-T$_{FBA1}$)+(P$_{TDH3}$-IDP2-T$_{CYC1}$)+(P$_{TEF1}$-YHM2-T$_{GAL1}$); pgi1Δ::(P$_{COX9}$-PGI1)+(P$_{TDH3}$-GND1-T$_{CYC1}$)+(P$_{tHXT7}$-TKL1-T$_{TDH2}$)+(P$_{PGK1}$-TAL1-T$_{ADH1}$)+(P$_{TEF1}$-ZWF1); idh2Δ:: P$_{GSY1}$-IDH2;XII-4:: (P$_{ADH6}$-per1ADH5-T$_{ADH1}$)+(P$_{ADH6}$-per2MaFAR1-T$_{CYC1}$); VII-2:: (T$_{ADH1}$-per1ADH5-P$_{GAL1,10}$-per2MaFAR1-T$_{CYC1}$);VIII-2::(P$_{BDH2}$-per1ADH5-T$_{ADH1}$)+ (P$_{BDH2}$-per2MaFAR1-T$_{CYC1}$); XI-2::(P$_{HXT7}$-PXA1-T$_{ADH1}$)+(P$_{HXT7}$-PXA2-T$_{FBA1}$); XI-6::(T$_{CPS1}$-per2PYC1-P$_{GAL1,10}$-per2RtME-T$_{PRM9}$); XI-8::(T$_{CPS1}$-IDP3-P$_{GAL1,10}$-per2IDP2-T$_{PRM9}$)=GNFOH119+ XI-8::(T$_{CPS1}$-IDP3-P$_{GAL1,10}$-per2IDP2-T$_{PRM9}$)	This study
GNFOH121	MATa; MAL2-8c; SUC2; hfd1Δ; gal80Δ; gal1Δ; gal7Δ; his3Δ::(HIS3-T$_{ENO2}$)+(P$_{ADH2}$-PEX28-T$_{HIS3}$); ura3Δ::(P$_{TPI1}$-RtFAS1-T$_{FBA1}$)+ (P$_{TEF1}$-RtFAS2-T$_{CYC1}$)+amdSym; X1-5::P$_{TEF1}$-Cas9-T$_{CYC1}$; acc1::P$_{TEF1}$-ACC1; pyc1::P$_{TEF1}$-PYC1; XI-4::(P$_{TPI1}$-MPC1)+(P$_{PGK1}$-MPC3-T$_{DIT1}$); X-2::(P$_{GAL1}$-AnACLa-T$_{CYC1}$)+(P$_{GAL10}$-AnACLb-T$_{ADH1}$); gal10Δ::(P$_{TPI1}$-RtCIT1-T$_{FBA1}$)+(P$_{TDH3}$-IDP2-T$_{CYC1}$)+(P$_{TEF1}$-YHM2-T$_{GAL1}$); pgi1Δ::(P$_{COX9}$-PGI1)+(P$_{TDH3}$-GND1-T$_{CYC1}$)+(P$_{tHXT7}$-TKL1-T$_{TDH2}$)+(P$_{PGK1}$-TAL1-T$_{ADH1}$)+(P$_{TEF1}$-ZWF1); idh2Δ:: P$_{GSY1}$-IDH2;XII-4:: (P$_{ADH6}$-per1ADH5-T$_{ADH1}$)+(P$_{ADH6}$-per2MaFAR1-T$_{CYC1}$); VII-2:: (T$_{ADH1}$-per1ADH5-P$_{GAL1,10}$-per2MaFAR1-T$_{CYC1}$);VIII-2::(P$_{BDH2}$-per1ADH5-T$_{ADH1}$)+ (P$_{BDH2}$-per2MaFAR1-T$_{CYC1}$); XI-2::(P$_{HXT7}$-PXA1-T$_{ADH1}$)+(P$_{HXT7}$-PXA2-T$_{FBA1}$); XI-6::(T$_{CPS1}$-per2PYC1-P$_{GAL1,10}$-per2RtME-T$_{PRM9}$); XI-8::(T$_{CPS1}$-IDP3-P$_{GAL1,10}$-per2IDP2-T$_{PRM9}$);IX-1::P$_{TEF1}$-MaFAR1-T$_{CYC1}$ =GNFOH120+IX-1::P$_{TEF1}$-MaFAR1-T$_{CYC1}$	This study
GNFOH122	MATa; MAL2-8c; SUC2; hfd1Δ; gal80Δ; gal1Δ; gal7Δ; his3Δ::(HIS3-T$_{ENO2}$)+(P$_{ADH2}$-PEX28-T$_{HIS3}$); ura3Δ::(P$_{TPI1}$-RtFAS1-T$_{FBA1}$)+ (P$_{TEF1}$-RtFAS2-	This study

	T$_{CYC1}$)+amdSym; X1-5::P$_{TEF1}$-Cas9-T$_{CYC1}$; acc1::P$_{TEF1}$-ACC1; pyc1::P$_{TEF1}$-PYC1; XI-4::(P$_{TPI1}$-MPC1)+(P$_{PGK1}$-MPC3-T$_{DIT1}$); X-2::(P$_{GAL1}$-AnACLa-T$_{CYC1}$)+(P$_{GAL10}$-AnACLb-T$_{ADH1}$); gal10Δ::(P$_{TPI1}$-RtCIT1-T$_{FBA1}$)+(P$_{TDH3}$-IDP2-T$_{CYC1}$)+(P$_{TEF1}$-YHM2-T$_{GAL1}$); pgi1Δ::(P$_{COX9}$-PGI1)+(P$_{TDH3}$-GND1-T$_{CYC1}$)+(P$_{tHXT7}$-TKL1-T$_{TDH2}$)+(P$_{PGK1}$-TAL1-T$_{ADH1}$)+(P$_{TEF1}$-ZWF1); idh2Δ:: P$_{GSY1}$-IDH2;XII-4:: (P$_{ADH6}$-per1ADH5-T$_{ADH1}$)+(P$_{ADH6}$-per2MaFAR1-T$_{CYC1}$); VII-2:: (T$_{ADH1}$-per1ADH5-P$_{GAL1,10}$-per2MaFAR1-T$_{CYC1}$);VIII-2::(P$_{BDH2}$-per1ADH5-T$_{ADH1}$)+ (P$_{BDH2}$-per2MaFAR1-T$_{CYC1}$); XI-2::(P$_{HXT7}$-PXA1-T$_{ADH1}$)+(P$_{HXT7}$-PXA2-T$_{FBA1}$); XI-6::(T$_{CPS1}$-per2PYC1-P$_{GAL1,10}$-per2RtME-T$_{PRM9}$); XI-8::(T$_{CPS1}$-IDP3-P$_{GAL1,10}$-per2IDP2-T$_{PRM9}$);IX-1::P$_{TDH3}$-MaFAR1-T$_{CYC1}$ =GNFOH120+IX-1::P$_{TDH3}$-MaFAR1-T$_{CYC1}$	
GNFOH132	MATa; MAL2-8c; SUC2; his3Δ1; ura3-52; X1-5::Cas9; gal80Δ; hfd1Δ;pYX212=GN15+ pYX212	This study
GNFOH134	MATa; MAL2-8c; SUC2; his3Δ1; ura3-52; X1-5::Cas9; gal80Δ; hfd1Δ;pAOH22=GN15+ pAOH22	This study
GNFOH136	MATa; MAL2-8c; SUC2; his3Δ1; ura3-52; X1-5::Cas9; gal80Δ; hfd1Δ;per2-pYX212-MaFAR1=GN15+ per2-pYX212-MaFAR1	This study
SH17h	MATa; MAL2-8c; SUC2; ura3Δ; hfd1Δ; gal80Δ; gal1Δ; gal7Δ; ’tesAΔ; his3Δ::(P$_{TPI}$-MmACL-T$_{FBA1}$)+(P$_{TDH3}$-RtME-T$_{CYC1}$)+(P$_{tHXT7}$-’MDH3-T$_{TDH2}$)+(P$_{PGK1}$-CTP1-T$_{HIS3}$); X1-5::P$_{TEF1}$-Cas9-T$_{CYC1;}$ acc1::P$_{TEF1}$-ACC1; pyc1::P$_{TEF1}$-PYC1; X1-4::(P$_{TPI1}$-MPC1)+(P$_{PGK1}$-MPC3-T$_{DIT1}$); X1-2::(P$_{GAL1}$-AnACLa-T$_{CYC1}$)+(P$_{GAL10}$-AnACLb-T$_{ADH1}$); gal10Δ::(P$_{TPI1}$-RtCIT1-T$_{FBA1}$)+(P$_{TDH3}$-IDP2-T$_{CYC1}$)+(P$_{TEF1}$-YHM2-T$_{GAL1}$); pgi1Δ::(P$_{COX9}$-PGI1)+(P$_{TDH3}$-GND1-T$_{CYC1}$)+(P$_{tHXT7}$-TKL1-T$_{TDH2}$)+(P$_{PGK1}$-TAL1-T$_{ADH1}$)+(P$_{TEF1}$-ZWF1); idh2Δ:: P$_{GSY1}$-IDH2; XI-1::P$_{GAL7}$-MCRN-T$_{DIT1}$; P$_{FAS1}$::P$_{HXT1}$; XII-3::P$_{TDH3}$-MCRN-T$_{FBA1}$+T$_{DIT1}$-MCRC-P$_{TDH3}$; his3::HIS3; XII-5::T$_{FBA1}$-MCRC-P$_{GAL1,10}$-MCRC-T$_{DIT1}$	Yu et al., 2022[5]
GNMCR5	MATa; MAL2-8c; SUC2; ura3Δ; hfd1Δ; gal80Δ; gal1Δ; gal7Δ; ’tesAΔ; his3Δ::(P$_{TPI}$-MmACL-T$_{FBA1}$)+(P$_{TDH3}$-RtME-T$_{CYC1}$)+(P$_{tHXT7}$-’MDH3-T$_{TDH2}$)+(P$_{PGK1}$-CTP1-T$_{HIS3}$); X1-5::P$_{TEF1}$-Cas9-T$_{CYC1;}$ acc1::P$_{TEF1}$-ACC1; pyc1::P$_{TEF1}$-PYC1; X1-4::(P$_{TPI1}$-MPC1)+(P$_{PGK1}$-MPC3-T$_{DIT1}$); X1-2::(P$_{GAL1}$-AnACLa-T$_{CYC1}$)+(P$_{GAL10}$-AnACLb-T$_{ADH1}$); gal10Δ::(P$_{TPI1}$-RtCIT1-T$_{FBA1}$)+(P$_{TDH3}$-IDP2-T$_{CYC1}$)+(P$_{TEF1}$-YHM2-T$_{GAL1}$); pgi1Δ::(P$_{COX9}$-PGI1)+(P$_{TDH3}$-GND1-T$_{CYC1}$)+(P$_{tHXT7}$-TKL1-T$_{TDH2}$)+(P$_{PGK1}$-TAL1-T$_{ADH1}$)+(P$_{TEF1}$-ZWF1); idh2Δ:: P$_{GSY1}$-IDH2; XI-1::P$_{GAL7}$-MCRN-T$_{DIT1}$; P$_{FAS1}$::P$_{HXT1}$; XII-3::P$_{TDH3}$-MCRN-T$_{FBA1}$+T$_{DIT1}$-MCRC-P$_{TDH3}$; his3::HIS3; XII-5::T$_{FBA1}$-MCRC-P$_{GAL1,10}$-MCRC-T$_{DIT1}$;IX-1:: P$_{AAD6}$-MCRN-T$_{TDH2}$+T$_{DIT1}$-	This study

	MCRC-P_{AAD6}= SH17h+ IX-1:: P_{AAD6}-MCRN-T_{TDH2}+T_{DIT1}-MCRC-P_{AAD6}	
GNMCR6	MATa; MAL2-8c; SUC2; ura3Δ; hfd1Δ; gal80Δ; gal1Δ; gal7Δ; 'tesAΔ; his3Δ::(P_{TPI}-MmACL-T_{FBA1})+(P_{TDH3}-RtME-T_{CYC1})+(P_{tHXT7}-'MDH3-T_{TDH2})+(P_{PGK1}-CTP1-T_{HIS3}); X1-5::P_{TEF1}-Cas9-$T_{CYC1;}$ acc1::P_{TEF1}-ACC1; pyc1::P_{TEF1}-PYC1; X1-4::(P_{TPI1}-MPC1)+(P_{PGK1}-MPC3-T_{DIT1}); X1-2::(P_{GAL1}-AnACLa-T_{CYC1})+(P_{GAL10}-AnACLb-$T_{ADH1)}$; gal10Δ::(P_{TPI1}-RtCIT1-T_{FBA1})+(P_{TDH3}-IDP2-T_{CYC1})+(P_{TEF1}-YHM2-T_{GAL1}); pgi1Δ::(P_{COX9}-PGI1)+(P_{TDH3}-GND1-$T_{CYC1)}$+(P_{tHXT7}-TKL1-T_{TDH2})+(P_{PGK1}-TAL1-T_{ADH1})+(P_{TEF1}-ZWF1); idh2Δ:: P_{GSY1}-IDH2; XI-1::P_{GAL7}-MCRN-T_{DIT1}; P_{FAS1}::P_{HXT1}; XII-3::P_{TDH3}-MCRN-T_{FBA1}+T_{DIT1}-MCRC-P_{TDH3}; his3::HIS3; XII-5::T_{FBA1}-MCRC-$P_{GAL1,10}$-MCRC-T_{DIT1}; IX-1:: P_{ADH6}-MCRN-T_{TDH2}+T_{DIT1}-MCRC-P_{ADH6}= SH17h+ IX-1:: P_{ADH6}-MCRN-T_{TDH2}+T_{DIT1}-MCRC-P_{ADH6}	This study
GNMCR7	MATa; MAL2-8c; SUC2; ura3Δ; hfd1Δ; gal80Δ; gal1Δ; gal7Δ; 'tesAΔ; his3Δ::(P_{TPI}-MmACL-T_{FBA1})+(P_{TDH3}-RtME-T_{CYC1})+(P_{tHXT7}-'MDH3-T_{TDH2})+(P_{PGK1}-CTP1-T_{HIS3}); X1-5::P_{TEF1}-Cas9-$T_{CYC1;}$ acc1::P_{TEF1}-ACC1; pyc1::P_{TEF1}-PYC1; X1-4::(P_{TPI1}-MPC1)+(P_{PGK1}-MPC3-T_{DIT1}); X1-2::(P_{GAL1}-AnACLa-T_{CYC1})+(P_{GAL10}-AnACLb-$T_{ADH1)}$; gal10Δ::(P_{TPI1}-RtCIT1-T_{FBA1})+(P_{TDH3}-IDP2-T_{CYC1})+(P_{TEF1}-YHM2-T_{GAL1}); pgi1Δ::(P_{COX9}-PGI1)+(P_{TDH3}-GND1-$T_{CYC1)}$+(P_{tHXT7}-TKL1-T_{TDH2})+(P_{PGK1}-TAL1-T_{ADH1})+(P_{TEF1}-ZWF1); idh2Δ:: P_{GSY1}-IDH2; XI-1::P_{GAL7}-MCRN-T_{DIT1}; P_{FAS1}::P_{HXT1}; XII-3::P_{TDH3}-MCRN-T_{FBA1}+T_{DIT1}-MCRC-P_{TDH3}; his3::HIS3; XII-5::T_{FBA1}-MCRC-$P_{GAL1,10}$-MCRC-T_{DIT1}; IX-1::P_{BDH2}-MCRN-T_{TDH2}+T_{DIT1}-MCRC-P_{BDH2}= SH17h+ IX-1::P_{BDH2}-MCRN-T_{TDH2}+T_{DIT1}-MCRC-P_{BDH2}	This study
GNMCR8	MATa; MAL2-8c; SUC2; ura3Δ; hfd1Δ; gal80Δ; gal1Δ; gal7Δ; 'tesAΔ; his3Δ::(P_{TPI}-MmACL-T_{FBA1})+(P_{TDH3}-RtME-T_{CYC1})+(P_{tHXT7}-'MDH3-T_{TDH2})+(P_{PGK1}-CTP1-T_{HIS3}); X1-5::P_{TEF1}-Cas9-$T_{CYC1;}$ acc1::P_{TEF1}-ACC1; pyc1::P_{TEF1}-PYC1; X1-4::(P_{TPI1}-MPC1)+(P_{PGK1}-MPC3-T_{DIT1}); X1-2::(P_{GAL1}-AnACLa-T_{CYC1})+(P_{GAL10}-AnACLb-$T_{ADH1)}$; gal10Δ::(P_{TPI1}-RtCIT1-T_{FBA1})+(P_{TDH3}-IDP2-T_{CYC1})+(P_{TEF1}-YHM2-T_{GAL1}); pgi1Δ::(P_{COX9}-PGI1)+(P_{TDH3}-GND1-$T_{CYC1)}$+(P_{tHXT7}-TKL1-T_{TDH2})+(P_{PGK1}-TAL1-T_{ADH1})+(P_{TEF1}-ZWF1); idh2Δ:: P_{GSY1}-IDH2; XI-1::P_{GAL7}-MCRN-T_{DIT1}; P_{FAS1}::P_{HXT1}; XII-3::P_{TDH3}-MCRN-T_{FBA1}+T_{DIT1}-MCRC-P_{TDH3}; his3::HIS3; XII-5::T_{FBA1}-MCRC-$P_{GAL1,10}$-MCRC-T_{DIT1}; IX-1::P_{STE3}-MCRN-T_{TDH2}+T_{DIT1}-MCRC-P_{STE3}= SH17h+IX-1::P_{STE3}-MCRN-T_{TDH2}+T_{DIT1}-MCRC-P_{STE3}	This study

Table S4 Plasmids used in this study.

**gRNA plasmids**			
**Name**	**Marker**	**Targeted gene**	**20 bp spacer**
pgRNA-hfd1	*Amp*^{R}, *ScURA3*	*HFD1*	TTTGACCTAATATTCTACAC/AGGGTAAAATCATTCCAATA
PgRNA- *KanMX*	*Amp*^{R}, *ScURA3*	*KanMX*	TCTTTCCAGACTTGTTCAAC/TACCCATGGTTGTTTATGTT
pgRNAVII-2	*Amp*^{R}, *ScURA3*	neutral sites	TGGGATTTCACAACTATTGA/TGAGCGTAATATGAATGTGA
pgRNAVIII-2	*Amp*^{R}, *ScURA3*	neutral sites	GAGGACAGCGTGAATCACAA/TCAATAGACACTGAAATGTA
pgRNAXII-4	*Amp*^{R}, *ScURA3*	neutral sites	ATTTGTACAATTCCCCATTA/GTTACCCGCGCTATTTCACA
pgRNAIX-1	*Amp*^{R}, *ScURA3*	neutral sites	GCGGGTTCTCTTAGTAAATG/CTTACCGACAATGATGTGAG
pgRNAXI-2	*Amp*^{R}, *ScURA3*	neutral sites	ACAAGACTAGGCAAAAGCCA/ACCAGTTGATCAGTTGAGGG
pgRNAXI-3	*Amp*^{R}, *ScURA3*	neutral sites	GATATGTCTCTAATTTTGGA/CCATTTTATTTTTGAGGATT
pgRNAXI-6	*Amp*^{R}, *ScURA3*	neutral sites	AAACTTACCGAATTGTAGCG/TAGTAAATACAACTATTGGA
pgRNAXI-8	*Amp*^{R}, *ScURA3*	neutral sites	TACTATCAGTAACACGACAA/GATGAAATAGCCTCAGTTAC

**Expression plasmids**	
**Name**	**Description**
pESC	2 µm, AmpR, *HIS3*, *P*$_{GAL1,10}$, T$_{CYC1}$, T$_{ADH1}$
pYX212	2 µm, AmpR, *URA3*, P$_{TPI}$, T$_{pYX212}$
pAOH9	pYX212-(P$_{TPI}$-*npgA*-T$_{FBA1}$)+(P$_{TDH3}$-*MmCAR*-T$_{ADH1}$)+(P*t*$_{HXT7}$-*ADH5*-T$_{CYC1}$)+(P$_{TEF1}$-*FacoAR*-T$_{pYX212}$)
pAOH20	pYX212-(P$_{TPI}$-*npgAper21*-T$_{FBA1}$)+(P$_{TDH3}$-*MmCARper22*-T$_{ADH1}$)+(P*t*$_{HXT7}$-*ADH5per21*-T$_{CYC1}$)+(P$_{TEF1}$-*FacoARper22*-T$_{pYX212}$)
pAOH21	pYX212-(P$_{GAL3}$-*npgA*-T$_{FBA1}$)+(P$_{GAL7}$-*MmCAR*-T$_{ADH1}$)+(P*GAL10*-*ADH5*-T$_{CYC1}$)+(P$_{GAL1}$-*FacoAR*-T$_{pYX212}$)
pAOH22	pYX212-(P$_{GAL3}$-*npgAper21*-T$_{FBA1}$)+(P$_{GAL7}$-*MmCARper22*-T$_{ADH1}$)+(P*GAL10*-*ADH5per21*-T$_{CYC1}$)+(P$_{GAL1}$-*FacoARper22*-T$_{pYX212}$)
PMaFAR1	pESC-(*P*$_{GAL1}$-*MaFAR1*- T$_{CYC1}$)
pMaMaqu2220	pESC-(*P*$_{GAL1}$-*MaMaqu2220*-T$_{CYC1}$)
pMmFAR1	pESC-(*P*$_{GAL1}$-*MmFAR1*-T$_{CYC1}$)
pTaFAR1	pESC-(*P*$_{GAL1}$-*TaFAR1*-T$_{CYC1}$)
pYX212-MaFAR1	pYX212-(*P*$_{GAL1}$-*MaFAR1*-T$_{CYC1}$)
per2-pYX212-MaFAR1	pYX212-(*P*$_{GAL1}$-*per2MaFAR1*-T$_{CYC1}$)+(*P*$_{GAL10}$-*per21ADH5*-T$_{ADH1}$)
**Plasmids for screening responsive promoters**	
pYX312	ori, f1 ori, 2µ, Amp^{R}, URA3, P$_{UAS-TDH3}$

pSC01	pYX312-eGFP at *Hin*d III/*Sal* I
pSC02	pYX312-P_{SPG1}-*eGFP*
pSC03	pYX312-P_{ADH6}-*eGFP*
pSC04	pYX312-P_{ARI1}-*eGFP*
pSC05	pYX312-P_{OYE3}-*eGFP*
pSC06	pYX312-P_{GAP1}-*eGFP*
pSC07	pYX312-P_{INO1}-*eGFP*
pSC08	pYX312-P_{DET1}-*eGFP*
pSC09	pYX312-P_{SIT1}-*eGFP*
pSC10	pYX312-P_{ASC1}-*eGFP*
pSC11	pYX312-P_{CIS3}-*eGFP*
pSC12	pYX312-P_{DAL5}-*eGFP*
pSC13	pYX312-P_{MEP2}-*eGFP*
pSC14	pYX312-P_{GDH3}-*eGFP*
pSC15	pYX312-P_{CWP1}-*eGFP*
pSC16	pYX312-P_{BDH2}-*eGFP*
pSC17	pYX312-P_{STE3}-*eGFP*
pSC18	pYX312-P_{PDR5}-*eGFP*
pSC19	pYX312-P_{FIT2}-*eGFP*
pSC20	pYX312-P_{HSP31}-*eGFP*
pSC21	pYX312-P_{AAD6}-*eGFP*

Table S5 Primers used in this study.

Primer	Primer name	Sequence (5’-3’)
Primers for gRNA plasmid construction		
P1	Grna-R1	GCGGTTAGCTCCTTCGGTCCTCCGATCGTTGTCAG AAGTAAGTTGGCCGCAGTGTTATC
P2	Grna-R2	GATAACACTGCGGCCAACTTACTTCTGACAACGAT CGGAGGACCGAAGGAGCTAACCGC
P3	Backbone	GATCATTTATCTTTCACTGCGGAGAAG
P4	gRNA-F1/2	AAACTTCTCCGCAGTGAAAGATAAATGATC ***20 bp spacer*** GTTTTAGAGCTAGAAATAG
Primers for donor DNA construction		
pJQ45	HIS3-F	CTCTTGGCCTCCTCTAGTACACTC
pJQ46	HIS3-R	CAGAAAAGACTAATAATTCTTAGTTAAAAGCACTC TACATAAGAACACCTTTGGTGGAG
pJQ47	T_{ENO2}-F	AGTGCTTTTAACTAAGAATTATTAGTC
pJQ48	T_{ENO2}-R	AGGTATCATCTCCATCTCCCATATG
pJQ49	T_{PRM9}-F	AGTGATATGCATATGGGAGATGGAGATGATACCTAT TTTCAACATCGTATTTTCCGAAG
pJQ50	T_{PRM9}-R	ACAGAAGACGGGAGACACTAGCAC
pJQ51	PEX7-F	GTAAAGTTGTGTGCTAGTGTCTCCCGTCTTCTGTTC AACCTAAGCCGTTCCATAC
pJQ52	PEX7-R	CTATCAACTATTAACTATATCGTAATACACACAAAAT GCTCAGATATCATATGCAAGG
pJQ53	P_{ADH2}-F	TTTGTGTGTATTACGATATAGTTAATAG
pJQ54	P_{ADH2}-R	TTAATATCTTAACTGATAGTTTGATCAAAGGG
pJQ55	P_{ADH2}	CCCTTTGATCAAACTATCAGTTAAGATATTAAGCAA AACGTAGGGGCAAACAAACGG
pJQ56	PEX28-F	CTATCAACTATTAACTATATCGTAATACACACAAAAT GAGTGAGACCAGCTCAAGTCGC
pJQ57	PEX28-R	TATATATCGTATGCTGCAGCTTTAAATAATCGGTGTC TTATGCTACTGGTGTTGCATTC
pJQ58	T_{HIS3}-F	GACACCGATTATTTAAAGCTGCAGC
pJQ59	T_{HIS3}-R	CTGTTATTTCTGGCACTTCTTGGTTTTCC
pJQ60	PEX7	CTATCAACTATTAACTATATCGTAATACACACAAAA CAGAAGACGGGAGACACTAGCAC
pJQ61	PEX28	CAACTATTAACTATATCGTAATACACACAAAGACAC CGATTATTTAAAGCTGCAGC
P5	HFD1up-F	AGAGAAGCTAGATTATCATTACAGCAGC
P6	HFD1up-R	ACATGTTGGTGATAAATTACTATGGCTATGG
P7	HFD1dw-F	ACCATAGCCATAGTAATTTATCACCAACATGTCGCT CATTGTTCTTCCTGAACGTAATG
P8	HFD1dw-R	TGTCTCCGAATCGTTCAGACATT
P9	HFD1-JDF	CCTTCAAAGGACATCATTGCCGATTATC
P10	HFD1-JDR	GTAGACAGGCAAATATCCCTCCACC
P11	HIS3up-F	CTCTTGGCCTCCTCTAGTACACTC
P12	HIS3up-R	CAGAAAAGACTAATAATTCTTAGTTAAAAGCACTC TACATAAGAACACCTTTGGTGGAG
P13	T_{ENO2}-F	AGTGCTTTTAACTAAGAATTATTAGTC
P14	T_{ENO2}-R	AACGATTTTTCCGTTTGTTTGCCCCTACGTTTTGAG GTATCATCTCCATCTCCCATATG
P15	P_{ADH2}-F	GCAAAACGTAGGGGCAAACAAACGG

P16	P_{ADH2}-R	ATTGTTGCCGCATGCATCCTTG
P17	PEX28-F	CTATCAACTATTAACTATATCGTAATACACACAAAAT GAGTGAGACCAGCTCAAGTCGC
P18	PEX28-R	TATATATCGTATGCTGCAGCTTTAAATAATCGGTGTC TTATGCTACTGGTGTTGCATTC
P19	HIS3dw-F	GACACCGATTATTTAAAGCTGCAGC
P20	HIS3dw-R	CTGTTATTTCTGGCACTTCTTGGTTTTCC
P21	XII-4up-F	GATAAGGCCCGCATGGTTTC
P22	XII-4up-R	CTATCGCTGAACAGGAACTTAAGTAG
P23	T_{ADH1}-R-XII-4up	GTAGCTACTTAAGTTCCTGTTCAGCGATAGGAGCG ACCTCATGCTATACCTGAG
P24	T_{CYC1}-R	GGAGGGGAGGGTACACCAAATTCCACCTCTCTTCG AGCGTCCCAAAACC
P25	XII-4dw-F	AGAGGTGGAATTTGGTGTACCC
P26	XII-4dw-R	CTTCCGACTCTGTTGTTTCCTATTG
P27	per1ADH5-R	CTATATCATTTTCAATAGCGATTTTTAATATGCCTTC GCAAGTCATTCCTG
P28	P_{AAD6}-F	TCACTTGCAGCCAGTAACCAG
P29	P_{AAD6}-R	A TTAAAAATCGCTATTGAAAATGATATAG
P30	P_{AAD6}-R2	CTCACCACCTGATACCTGGCGCTTTGTCATATTAAA AATCGCTATTGAAAATGATATAG
P31	BS-F1	TAGGAGTCTGGTTACTGGCTGCAAGTGA
P32	BS-R1	AGGAGTCTGGTTACTGGCTGCAAGTGACCATTCGC CATTCAGGCTGC
P33	MaFAR1-F	ATGACAAAGCGCCAGGTATCAG
P34	per1ADH5-R-P_{AAD6}	TTCAACACAACAACAAGAAAAGCCAAAATCATGC CTTCGCAAGTCATTCCTG
P35	P_{ADH6}-F	CCATGGAGTGTTCTTGTATCCGTC
P36	P_{ADH6}-R	GATTTTGGCTTTTCTTGTTGTTGTGTTG
P37	BS-F2	TGATGACGGATACAAGAACACTCCATGGTATCTCG AGCTCAGCTAGCTAACTG
P38	BS-R2	GATGACGGATACAAGAACACTCCATGGCCATTCGC CATTCAGGCTGC
P39	P_{ADH6}-R-MaFAR1	CTCACCACCTGATACCTGGCGCTTTGTCATGATTTT GGCTTTTCTTGTTGTTGTGTTG
P40	per1ADH5-R-P_{BDH2}	TAACAATAAATTCATTGAACATATTTCAGAATGCCT TCGCAAGTCATTCCTG
P41	P_{BDH2}-F	GTACGTTGCAGGAGCACGC
P42	P_{BDH2}-R	TCTGAAATATGTTCAATGAATTTATTGTTATTC
P43	BS-F3	ATGGGCGTGCTCCTGCAACGTACTATCTCGAGCTC AGCTAGCTAACTG
P44	BS-R3	CCATGGGCGTGCTCCTGCAACGTACCCATTCGCCAT TCAGGCTGC
P45	P_{BDH2}-R-MaFAR1	CCACCTGATACCTGGCGCTTTGTCATTCTGAAATAT GTTCAATGAATTTATTGTTATTC
P46	Per1ADH5-R-P_{STE3}	TAGGAAAGGCAAAATACTATCAAAATTTTCATGCCT TCGCAAGTCATTCCTG
P47	P_{STE3}-F	ATTTTGTTGAAAATAGTATAAAGAGTGCAATG
P48	P_{STE3}-R	GAAAATTTTGATAGTATTTTGCCTTTCCTAC
P49	BS-F4	TGCACTCTTTATACTATTTTCAACAAAATTATCTCGA GCTCAGCTAGCTAACTG

P50	BS-R4	TTGCACTCTTTATACTATTTTCAACAAAATCCATTCGCCATTCAGGCTGC
P51	P_{STE3}-R-MaFAR1	CACCACCTGATACCTGGCGCTTTGTCATGAAAATTTTGATAGTATTTTGCCTTTCCTAC
P52	XII-4-JDF	CGTGTTCGTACCTGATGACGTATC
P53	XII-4-JDR	TGGAGGAATAGCCGCTCCC
P42	VII-2up-F	GCCGGTGTAAGGGTCGAC
P43	VII-2upR-T_{ADH1}	GGTCAGGTTGCTTTCTCAGGTATAGCATGAGGTCGCTCGTGACAGCTGCTGGAGCC
P44	T_{ADH1}-F-VII-2up	CAAACACCAATTGGTTTATTATTCTCTTTTCTTCAATTATAATCAGTACAATAATGGCTCCAGCAGCTGTCACGAGCGACCTCATGCTATACCTGAG
P45	T_{CYC1}-R-VII-2dw	CTAGTTTATTATTACCTGAGGTATAAGAAAATGACACAAATATTGGTAAACGGTGATCAGGCTTGGTGGAAGCCTTCGAGCGTCCCAAAACCTTC
P46	VII-2dw-F-T_{CYC1}	CTTGAGAAGGTTTTGGGACGCTCGAAGGCTTCCACCAAGCCTGATCAC
P47	VII-2dw-R	CTCCGGTATTACTCGAGCCCG
P48	VII-2-JDF	GGTCCATAGTAGCCAAGTGCATTC
P49	VII-2-JDR	GATTCCGCGCTTCCACCAC
P50	VIII-2up-F	GCGCCTTACCAACTTGGCC
P51	VIII-2upR-T_{ADH1}	CTTTCTCAGGTATAGCATGAGGTCGCTCGACCTAACAAAAGAACCTTTATTAACAATG
P52	T_{ADH1}-R	GAGCGACCTCATGCTATACCTGAG
P53	BS-F	TATCTCGAGCTCAGCTAGCTAACTG
P54	BS-R	CCATTCGCCATTCAGGCTGC
P55	T_{CYC1}-R	CTTCGAGCGTCCCAAAACCTTC
P56	VIII-2-JDF	CGGAGGGTGATCATCAGCTACG
P57	VIII-2-JDR	GGTGTCGTGATCGTTACCTATGC
P58	XI-6up-F	CCTCCTAAAGCTCGGTAAAGGGAAC
P59	XI-6upR	GATAAAAGCATTCACTGCCCGC
P60	XI-6upF-P_{GAL7}	TTCCTGCGGGCAGTGAATGCTTTTATCTTTGCCAGCTTACTATCCTTCTTGAAAATATG
P61	P_{GAL7}-R	TTTTGAGGGAATATTCAACTGTTTTTTTTTATCATGTTG
P62	P_{GAL7}-MDH3-F	TAAAAAAAAACAGTTGAATATTCCCTCAAAAATGGTCAAAGTCGCAATTCTTGG
P63	MDH3-R-T_{ENO2}	AAAGACTAATAATTCTTAGTTAAAAGCACTTCATAGCTTGGAAGAGTCTAGGATGAAAC
P64	T_{ENO2}-F	AGTGCTTTTAACTAAGAATTATTAGTCTTTTCTGC
P65	T_{ENO2}-R	AGGTATCATCTCCATCTCCCATATGC
P66	T_{CPS1}-R-T_{ENO2}	TGCATATGGGAGATGGAGATGATACCTATTTGACACTTGATTTGACACTTCTTTTTTTT
P67	T_{CPS1}-F-PYC1	AAACTATCGCAGGCAAAATCTAAACTATGAGCGCAATGATTGAATAGTCAAAGATTTTT
P68	PYC1-R-per2	TCATAGTTTAGATTTTGCCTGCGATAGTTTTACAGCGGCAGAACCACCACCTGCCTTAGTTTCAACAGGAA

		CTTGG
P69	PYC1-F-$P_{GAL1,10}$	GTAAGAATTTTTGAAAATTCAATATAACAAAATGTC GCAAAGAAAATTCGCCG
P70	$P_{GAL1,10}$-F	TTTGTTATATTGAATTTTCAAAAATTCTTACTTTTTT TTTGG
P71	$P_{GAL1,10}$-R	TTTGTATAGTTTTTTCTCCTTGACGTTAAAGTATAG
P72	RtME-F-$P_{GAL1,10}$	CTTTAACGTCAAGGAGAAAAAACTATACAAAATGC CTGCTCATTTTGCCC
P73	RtME-R-per2	TCATAGTTTAGATTTTGCCTGCGATAGTTTTACAGC GGCAGAACCACCACCTTGTGCTTGTTGTTCTGCTT CTAATAATGG
P74	PRM9t-F-RtME	AAACTATCGCAGGCAAAATCTAAACTATGAACAGA AGACGGGAGACACTAGC
P75	T_{PRM9}-F	ACAGAAGACGGGAGACACTAGC
P76	T_{PRM9}-R	ATTTTCAACATCGTATTTTCCGAAGCG
P77	XI6-dwF-T_{PRM9}	CAACGCTTCGGAAAATACGATGTTGAAAATGCTAA GGCGAGAAAACTGCTG
P78	XI-6dw-R	CAACCATAGTGGCCAATTTTGACAC
P79	T_{CPS1}-R-XI-6up	CTTCCTGCGGGCAGTGAATGCTTTTATCATTTGACA CTTGATTTGACACTTCTTTTTTT
P80	T_{CPS1}-F-$P_{GAL1,10}$	TAAGAATTTTTGAAAATTCAATATAACAAAGCGCA ATGATTGAATAGTCAAAGATTTTT
P81	XI-6-JDF	GTGGTCCAAGCTTCGCTGTG
P82	XI-6-JDR	GGCCACCGACACTGTACTG
P83	XI-8up-F	CAGCTTCCCCTGACTTCGATG
P84	XI-8upR-T_{CPS1}	TAAAAAAAAAAAGAAGTGTCAAATCAAGTGTCAA ATCTGCAACCCTTCGATTGCAATC
P85	T_{CPS1}-F	GCGCAATGATTGAATAGTCAAAGATTTT
P86	T_{CPS1}-R	ATTTGACACTTGATTTGACACTTCTTTTTT
P87	IDP3-R-T_{CPS1}	AAAAATCTTTGACTATTCAATCATTGCGCTTATAGTT TGCACATACCTTTCTTGTCTTC
P88	IDP3-F-$P_{GAL1,10}$	AGAATTTTTGAAAATTCAATATAACAAAATGAGTAA AATTAAAGTTGTTCATCCCATCG
P89	IDP2-F-$P_{GAL1,10}$	CTTTAACGTCAAGGAGAAAAAACTATACAAAATGA CAAAGATTAAGGTAGCTAACCCC
P90	IDP2-R-per2	TTATAGTTTAGATTTTGCCTGCGATAGTTTTACAGCG GCAGAACCACCACCCAATGCAGCTGCCTCGAAC
P91	T_{PRM9}-F-IDP2	AAACTATCGCAGGCAAAATCTAAACTATAAACAGA AGACGGGAGACACTAGC
P92	XI-8-dwF-T_{PRM9}	CAACGCTTCGGAAAATACGATGTTGAAAATGGCAT ATCGTTACCGCCTTTTGC
P93	XI-8-dwR	TCTCGCAGTAGAGGTAATTCACGATC
P94	XI-8up-R-$P_{GAL1,10}$	TAAGAATTTTTGAAAATTCAATATAACAAACTGCAA

		CCCTTCGATTGCAATC
P95	XI-8-dwF-$P_{GAL1,10}$	CTTTAACGTCAAGGAGAAAAAACTATACAAAGGCATATCGTTACCGCCTTTTG
P96	XI-8-JDF	AGAAGTGCCTCCTCGAGGATC
P97	XI-8-JDR	CCGCGCTATTTAGTAGGCTACTAC
P98	XI-3up-F	GAAGCATCGGTTCAGATCGAGC
P99	XI-3up-R-P_{GAL2}	CCCTGGTCCGTAAACCTCCTTGGATTAGGGCAACCAACTTTTTGCTGGC
P100	P_{GAL2}-F	CTAATCCAAGGAGGTTTACGGACC
P101	P_{GAL2}-R	TATGAAAGAATTATTTTTTTATTATGTTAATC
P102	FAA2-F-P_{GAL2}	TAACATAATAAAAAAAATAATTCTTTCATAATGGCCGCTCCAGATTATGC
P103	FAA2-R-T_{ADH1}	ATAAAAATCATAAATCATAAGAAATTCGCCTAAAGCTTTTCTGTCTTGACTAGTGAAC
P104	T_{ADH1}-F	GCGAATTTCTTATGATTTATGATTTTTATT
P105	T_{ADH1}-R	GCATATCTACAATTGGGTGAAATGG
P106	XI-3dw-F-T_{ADH1}	GCTCCCCATTTCACCCAATTGTAGATATGCACATGGAACTCCACAGAACTTGC
P107	XI-3dw-R	GATAGGACAGGAGCGATAGGAAAAG
P108	XI-2up-F	CACGGCCTCCTTACTAGCTG
P109	XI-2up-R-T_{ADH1}	CTCCCCATTTCACCCAATTGTAGATATGCCTGTTGAGTTACACCCCGCG
P110	PXA1-R-T_{ADH1}	AATAAAAATCATAAATCATAAGAAATTCGCTCAAATAATTTCAAGCTTTTCCCGTAGC
P111	PXA1-F-P_{HXT7}	ACAAAAAGTTTTTTTAATTTTAATCAAAAAATGTCAACAACATTAGCAGCACC
P112	P_{HXT7}-F	GATTGTTCCATACGGCTCCGTC
P113	P_{HXT7}-R	TTTTTGATTAAAATTAAAAAAACTTTTTG
P114	BS-F5	CAGCCTCTGACGGAGCCGTATGGAACAATCTATCTCGAGCTCAGCTAGCTAACTG
P115	BS-R5	CAGCCTCTGACGGAGCCGTATGGAACAATCCCATTCGCCATTCAGGCTGC
P116	PXA2-F-P_{HXT7}	ACAAAAAGTTTTTTTAATTTTAATCAAAAAATGATCTCAACAGCTTCTGCATTTTATC
P117	PXA2-R-T_{FBA1}	AAAAAACTATATCAATTAATTTGAATTAACTTACCTCTCCTTTCCTTCTTCCTTTTTTG
P118	T_{FBA1}-F	GTTAATTCAAATTAATTGATATAGTTTTTT
P119	T_{FBA1}-R	AGTAAGCTACTATGAAAGACTTTACAAAG
P120	XI-2dw-F-T_{FBA1}	CTTTGTAAAGTCTTTCATAGTAGCTTACTAAGTTTAGATGTAGGTTTTAGCGGTAAC
P121	XI-2dw-R	ATGAGCATTTGCTCACCTTCCTG
P122	XI-2-JDF	GGGTTCTAGAAGTGCCCTTTGAG
P123	XI-2-JDR	AGGAACCGAGGCACGGAAAC

P124	IX-1up-F	GTTTCGCCATAGCCATGTGCC
P125	IX-1up-R	GAGGCACAATTTTATGACCGCG
P126	P_{TDH3}-F- IX-1up	AAAACACGCGGTCATAAAATTGTGCCTCTCGAGTT TATCATTATCAATACTGCCATTTC
P127	P_{TDH3}-R-MaFAR1	CACCACCTGATACCTGGCGCTTTGTCATTTTGTTTG TTTATGTGTGTTTATTCGAAACT
P128	MaFAR1-F	ATGACAAAGCGCCAGGTATCAG
P129	IX-1dw-F- T_{CYC1}	TTGCTTGAGAAGGTTTTGGGACGCTCGAAGGGTCA AGCAGTGAGGAAGAAAGATCG
P130	IX-1dw-R	GTCCACAGTAAAATCGTTCAGGCC
P131	P_{TEF1}-F-IX-1up	AAACACGCGGTCATAAAATTGTGCCTCATAGCTTC AAAATGTTTCTACTCCTTTTTTAC
P132	P_{TEF1}-R-MaFAR1	ACCACCTGATACCTGGCGCTTTGTCATTTTGTAATT AAAACTTAGATTAGATTGCTAT
P133	P_{AAD6}-F-IX-1up	GAAAACACGCGGTCATAAAATTGTGCCTCTCACTT GCAGCCAGTAACCAG
P134	P_{AAD6}-R-ScMCRN	CCGGCCAACCTACCAGTTCCAGACATTTTTATTAAA AATCGCTATTGAAAATGATATAG
P135	ScMCRN-F	ATGTCTGGAACTGGTAGGTTGGC
P136	ScMCRN-R-T_{TDH2}	CATTAAAGTAACTTAAGGAGTTAAATTTAAATATTA GCTGGGATATTCAAGGTAATCTC
P137	T_{TDH2}-F	ATTTAACTCCTTAAGTTACTTTAATGATTTAG
P138	T_{TDH2}-R	GCGAAAAGCCAATTAGTGTGATAC
P139	T_{DIT1}-R-T_{TDH2}	CACTTAGTATCACACTAATTGGCTTTTCGCGTTACT CCGCAACGCTTTTCTG
P140	T_{DIT1}-F	TAAAGTAAGAGCGCTACATTGGTCTACC
P141	ScMCRC-R-T_{DIT1}	AAGGTAGACCAATGTAGCGCTCTTACTTTATTAGAC AGTGATAGCTCTTCCTCTATGG
P142	ScMCRC-F	AAAAATGTCTGCTACCACTGGTGCTAG
P143	P_{AAD6}-R-ScMCRC	ACCTAGCACCAGTGGTAGCAGACATTTTTATTAAA AATCGCTATTGAAAATGATATAGAAGAG
P144	P_{AAD6}-F-IX-1dw	CTGCCGATCTTTCTTCCTCACTGCTTGACCTCACTT GCAGCCAGTAACCAG
P145	IX-1dw-F	GGTCAAGCAGTGAGGAAGAAAGATC
P146	IX-1dw-R	GTCCACAGTAAAATCGTTCAGGCC
P147	P_{ADH6}-F-IX-1up	CGAAAACACGCGGTCATAAAATTGTGCCTCCCATG GAGTGTTCTTGTATCCGTC
P148	P_{ADH6}-R-ScMCRN	TCCGGCCAACCTACCAGTTCCAGACATTTTTGATTT TGGCTTTTCTTGTTGTTGTGTTG
P149	P_{ADH6}-R-ScMCRC	AGACCTAGCACCAGTGGTAGCAGACATTTTTGATT TTGGCTTTTCTTGTTGTTGTGTTG
P150	P_{ADH6}-F-IX-1dw	CTGCCGATCTTTCTTCCTCACTGCTTGACCCCATGG AGTGTTCTTGTATCCGTC

P151	P_{BDH2}-F-IX-1up	CGAAAACACGCGGTCATAAAATTGTGCCTCGTACG<br>TTGCAGGAGCACGC
P152	P_{BDH2}-R-ScMCRN	TTCCGGCCAACCTACCAGTTCCAGACATTTTGTCTG<br>AAATATGTTCAATGAATTTATTGTTATTC
P153	ScMCRC-R-Kozak	CAAAATGTCTGCTACCACTGGTGCTAG
P154	P_{BDH2}-R-ScMCRC	GCAGACCTAGCACCAGTGGTAGCAGACATTTTGTC<br>TGAAATATGTTCAATGAATTTATTGTTATTC
P155	P_{BDH2}-F-IX-1dw	CTGCCGATCTTTCTTCCTCACTGCTTGACCGTACGT<br>TGCAGGAGCACGC
P156	P_{STE3}-F-IX-1up	AAACACGCGGTCATAAAATTGTGCCTCATTTTGTTG<br>AAAATAGTATAAAGAGTGCAATG
P157	P_{STE3}-R-ScMCRN	TTCCGGCCAACCTACCAGTTCCAGACATTTTTGAA<br>AATTTTGATAGTATTTTGCCTTTCCTAC
P158	P_{STE3}-R-ScMCRC	CAGACCTAGCACCAGTGGTAGCAGACATTTTTGAA<br>AATTTTGATAGTATTTTGCCTTTCCTAC
P159	P_{STE3}-F-IX-1dw	CCGATCTTTCTTCCTCACTGCTTGACCATTTTGTTG<br>AAAATAGTATAAAGAGTGCAATG
P160	IX-1-JDF	GCCAATGACTGCAAATACGTTCG
P161	IX-1-JDR	CCCTGAAGCCACAACCACTAC
**Primers for screening responsive promoters**		
P162	eGFP-F	CCC***aagctt***ATGGTGAGCAAGGGCGAGG
P163	eGFP-R	ACGC***gtcgac***TTACTTGTACAGCTCGTCCATGCC
P164	P_{SPG1}-F	AAGGGAACAAAAGCTGGTACACCAAAACCGTCGA<br>GACAAG
P165	P_{SPG1}-R	CCTCGCCCTTGCTCACCATATTCAGTAAACTTTGTT<br>TGAATTTTATGC
P166	P_{ADH6}-F	AAGGGAACAAAAGCTGGTACCCATGGAGTGTTCTT<br>GTATC
P167	P_{ADH6}-R	CCTCGCCCTTGCTCACCATAGATTTTGGCTTTTCTT<br>GTTG
P168	P_{ARI1}-F	AAGGGAACAAAAGCTGGTACCAGAAGTATTCACC<br>TCCAAAC
P169	P_{ARI1}-R	CCTCGCCCTTGCTCACCATAAATTATTACTTTTTTAA<br>GTCTGTTTAGC
P170	P_{OYE3}-F	AAGGGAACAAAAGCTGGTACCGGTTGGATCCTCA<br>CTTG
P171	P_{OYE3}-R	CCTCGCCCTTGCTCACCATACTTCTAAATTTAAACT<br>TCGCTATACTG
P172	P_{GAP1}-F	AAGGGAACAAAAGCTGGTACAAAGTTAAAAGAAA<br>CTTTCTCATAC
P173	P_{GAP1}-R	CCTCGCCCTTGCTCACCATATTTTTATTTCTTTTTTT<br>TGTTTCTTATAAATG
P174	P_{INO1}-F	AAGGGAACAAAAGCTGGTACGGGAACGGGGGAA<br>AAGGTC
P175	P_{INO1}-R	CCTCGCCCTTGCTCACCATATGTTACTTCTTTTTCAC<br>TGGAAAAAAAAGG
P176	P_{DET1}-F	AAGGGAACAAAAGCTGGTACGAACATGAAGTCAT<br>TAAGTAGAC
P177	P_{DET1}-R	CCTCGCCCTTGCTCACCATATTCTTTTACTAATACTC<br>TGAGTTATTC

P178	P_{SIT1}-F	AAGGGAACAAAAGCTGGTACGTGCAGACTAATGAAAAAG
P179	P_{SIT1}-R	CCTCGCCCTTGCTCACCATATATGGAAAAATTTTCGTACTGG
P180	P_{ASC1}-F	AAGGGAACAAAAGCTGGTACAAATGTTGCTAAATTGGAGG
P181	P_{ASC1}-R	CCTCGCCCTTGCTCACCATATTTTCACTTTATTTACTTTAGTGTG
P182	P_{CIS3}-F	AAGGGAACAAAAGCTGGTACAAACGCGTCTAATTATGG
P183	P_{CIS3}-R	CCTCGCCCTTGCTCACCATATTTAGATGTAAGTTTAGCGAG
P184	P_{DAL5}-F	AAGGGAACAAAAGCTGGTACCCATATCTTCAGTGAGGATAAC
P185	P_{DAL5}-R	CCTCGCCCTTGCTCACCATACTTGAATTTTTTTTTTACACTATTTG
P186	P_{MEP2}-F	AAGGGAACAAAAGCTGGTACCTGTTAATATTTAAATTTCTGCTAGTC
P187	P_{MEP2}-R	CCTCGCCCTTGCTCACCATATGTTGATATTGTATTGTAATATATTAAGTATG
P188	P_{GDH3}-F	AAGGGAACAAAAGCTGGTACTAAAAACCGTCAAGGCATTTATC
P189	P_{GDH3}-R	CCTCGCCCTTGCTCACCATATTTTTACTTTTTACCTTTTCTTTTCG
P190	P_{CWP1}-F	AAGGGAACAAAAGCTGGTACTCCTCACTACAATTGCCC
P191	P_{CWP1}-R	CCTCGCCCTTGCTCACCATATATTGTTTTTTGAGACTTTCGTAG
P192	P_{BDH2}-F	AAGGGAACAAAAGCTGGTACGTACGTTGCAGGAGCACG
P193	P_{BDH2}-R	CCTCGCCCTTGCTCACCATATCTGAAATATGTTCAATGAATTTATTGTTATTCTTATTG
P194	P_{STE3}-F	AAGGGAACAAAAGCTGGTACATTTTGTTGAAAATAGTATAAAGAGTG
P195	P_{STE3}-R	CCTCGCCCTTGCTCACCATAGAAAATTTTGATAGTATTTTGCC
P196	P_{PDR5}-F	AAGGGAACAAAAGCTGGTACCGTTAACGTAAATATGTCTTC
P197	P_{PDR5}-R	CCTCGCCCTTGCTCACCATATTTTGTCTAAAGTCTTTCGAAC
P198	P_{FIT2}-F	AAGGGAACAAAAGCTGGTACACGCAAGACAACAGGCAAAATAATTTC
P199	P_{FIT2}-R	CCTCGCCCTTGCTCACCATATATTATTGTTTTGTGATGGCTTTATGATAGAC
P200	P_{HSP31}-F	AAGGGAACAAAAGCTGGTACATGTACCAGTATGTAATATTATATCAAG
P201	P_{HSP31}-R	CCTCGCCCTTGCTCACCATATATAAATTTGTTTGAGTTTTATCTGTG
P202	P_{AAD6}-F	AAGGGAACAAAAGCTGGTACTCACTTGCAGCCAGTAAC
P203	P_{AAD6}-R	CCTCGCCCTTGCTCACCATAATTAAAAATCGCTATTGAAAATGATATAG

Note: Bold and underlined sequences are 20 bp gRNA spacer and enzyme restriction sites.

Table S6 Codon optimized genes used in this study.

Gene	Sequence (5’-3’)
MaFAR1	ATGACAAAGCGCCAGGTATCAGGTGGTGAGGCCAGCAGCCGTGTCCTTGAGCAGCTAC<br>GTGGCAAGAAGGTCCTAATCACTGGCACGACGGGATTCCTAGGAAAGGTCGTGCTTGA<br>GAAGTTGATACGCGCAGTGCCGGACATAGGAGGCATCTACCTGTTGATCCGTGGAAACA<br>AACGTCATCCAAACGCGCGCTCGAGGTTTCTAAATGAGATAGCAACATCATCAGTATTTG<br>ATAGATTAAGAGAAGCTGATACAGAAGCTTTCGACGCATTCTTGGAGGATAGAATTCATT<br>GTGTAACAGGTGAAGTAACAGAACCTGGTTTTGGTTTAGGTGAACCTGCTTGTAGAAAG<br>CTGGCCATGGAATTAGATGCTGTAATTAATTCAGCGGCAAGCGTTAACTTTCGCGAAGA<br>GCTGGATAAAGCTCTGACGATAAACACCCTATGCTTGGAGAACATAGCTCAATTAGCTAG<br>AATGAATCCTGCTTTAGCTGTATTACAAGTATCAACATGTTATGTAAATGGTATGAATTCA<br>GGTCAAGTAGCTGAAGCTGTAATTAAACCTGCTGGTGAAGCTATTCCGCAGAGCCCGGA<br>GGGATACTACGAGATCGAGGACTTGGTCCGTCTCCTACACGACAAGATTGAGGACGTTC<br>GGTCGCGTTACACTGGCAAGGCGCTGGAGAAGAAGCTTGTTGAATTAGGTATTAGAGA<br>AGCTAATAGATATGGTTGGTCAGATACATATACATTTACAAAGTGGCTGGGTGAACAATT<br>ATTAATGAAAGCTTTACAAGGTAGATCATTAACAATTGTAAGACCTTCAATTATTGAATCA<br>GCTTTAGAAGAACCTGCTCCTGGTTGGATTGAAGGTGTAAAGGTCGCAGATGCTATTATA<br>CTCGCCTATGCTAGAGAGAAGGTTACCTTATTTCCTGGTAAGCGGTCTGGTATTATTGATG<br>TAATTCCTGTAGATTTAGTAGCTAATGCTATTATTATGGGTTTAGCTGAAGCTTTAGCTGA<br>ACCTGGTGAACAACATATTTATCAATGTTGTTCAGGTGGGTCCAACCCAGTAACACTGG<br>GCCAATTTATCGACCACCTAATGGAGGAGTCTAAGACGAACTACGCGGCATACGACCAC<br>TTGTTCTACAGGCAACCTTCAAAGCCATTTGTTGCCGTAAATAGAGGTTTATTTGATTTA<br>GTAGTCTCCGGCGTGCGACTTCCATTGTCAATCACTGACCGAGTCCTCAAATTACTAGG<br>GAACTCTCGAGACCTAAAGTGGCTACGCAACCTGGACACCACGCAATCCCTTGCAACT<br>ATATTTGGATTCTATACAGCGCCAGACTACGTGTTCCGGAACGATAGATTACAAGATTTA<br>GCTGAAAGAATGGGTCAAACAGACAAGGCACTCTTCCCAGTAGATCCTAAAGCTATCG<br>ACTGGCAGCACTACCTTCGGAAGATCCACCTCGCAGGCTTGAACCGGTACGCGTTGAA<br>AGAAAGAAAGGTGTACTCTTTAAAGAGTTCTAGACAAAGAAAGAAGGCGGCATGA

MaMaqu2220	ATGGCTATTCAACAAGTACATCATGCTGACACTAGCTCGTCTAAAGTACTGGGTCAGCTT<br>CGAGGCAAACGTGTGCTGATCACGGGAACCACTGGTTTCTTGGGCAAAGTAGTTCTAG<br>AACGCTTAATTCGAGCGGTGCCAGACATCGGCGCGATATACCTCCTCATCAGAGGTAATA<br>AGCGGCACCCTGATGCTAGATCAAGATTCCTTGAGGAGATCGCCACATCATCAGTATTTG<br>ATAGATTAAGAGAAGCTGATTCAGAAGGTTTCGACGCCTTCCTAGAGGAGCGTATCCAT<br>TGTGTAACAGGTGAAGTAACAGAAGCTGGTTTCGGGATAGGTCAAGAAGATTATAGAA<br>AGCTGGCGACAGAATTAGATGCTGTAATTAATTCAGCAGCGAGCGTTAACTTTAGGGAA<br>GAGCTTGACAAGGCGCTGGCAATCAACACTCTTTGCCTACGAAACATAGCGGGCATGGT<br>AGATTTAAATCCTAAATTAGCTGTATTACAAGTATCAACATGTTATGTAAATGGTATGAAT<br>TCAGGTCAAGTAACAGAATCAGTAATTAAACCTGCTGGTGAAGCTGTACCGCGAAGCCC<br>AGATGGTTTCTACGAGATCGAGGAGCTAGTTAGGCTACTCCAGGACAAGATCGAGGACG<br>TGCAGGCACGGTACTCCGGCAAGGTATTGGAGCGTAAACTCGTGGATTTAGGTATTAGA<br>GAAGCTAATAGATATGGTTGGTCCGACACCTACACGTTCACCAAATGGCTGGGCGAGCA<br>GCTACTCATGAAGGCCCTTAACGGGCGGACGCTTACCATATTAAGACCTTCAATTATTGA<br>ATCAGCTTTAGAAGAACCTGCTCCTGGTTGGATTGAAGGTGTAAAGGTTGCAGATGCTA<br>TTATACTTGCGTATGCTAGAGAGAAGGTCACGTTATTTCCTGGTAAGCGCAGTGGAATAA<br>TCGACGTCATACCCGTGGACCTCGTGGCAAACAGTATCATCCTTTCTTTAGCTGAAGCTT<br>TAGGTGAACCTGGTAGAAGAAGAATTTATCAATGTTGTTCAGGTGGTGGTAATCCTATTT<br>CATTAGGTGAATTTATTGATCATTTAATGGCCGAGAGCAAAGCCAATTACGCCGCCTACG<br>ACCACTTGTTCTACCGGCAGCCCAGCAAACCATTCCTAGCTGTTAACCGGGCCCTGTTC<br>GACCTAGTTATCTCTGGCGTTCGGCTCCCGCTTTCGTTGACTGACAGGGTCTTGAAACT<br>GTTGGGCAACAGTAGGGACCTAAAGATGCTGCGGAACCTAGACACTACTCAATCTCTTG<br>CCACCATCTTTGGCTTCTATACGGCTCCCGATTACATATTCCGTAACGATGAATTAATGGC<br>TTTAGCTAATAGAATGGGTGAAGTAGATAAAGGGTTGTTTCCTGTTGACGCACGGCTTAT<br>AGACTGGGAGCTTTACCTGCGGAAGATCCACTTGGCAGGGTTGAACCGTTACGCCCTCA<br>AGGAGCGCAAGGTTTATTCCTTAAAGACTGCCAGACAAAGAAAGAAGGCCGCCTGA

MmFAR1	ATGGTTAGCATACCGGAGTACTACGAGGGAAAGAACATTCTACTTACCGGCGCAACCGG<br>ATTCCTCGGCAAGGTGCTTCTTGAGAAGCTGTTACGTTCCTGCCCGCGCGTTAACAGTG<br>TCTATGTATTAGTAAGACAGAAGGCGGGCCAGACTCCGCAGGAGCGGGTTGAGGAGAT<br>CTTATCCAGCAAATTATTTGACCGCCTCAGGGACGAGAACCCGGACTTTCGGGAGAAGA<br>TCATAGCAATAAACTCTGAGCTTACTCAGCCAAAGCTTGCCTTGTCTGAGGAGGACAAG<br>GAGATAATCATCGACTCTACCAACGTTATCTTCCACTGCGCGGCGACGGTGCGATTCAAC<br>GAGAACCTTCGTGACGCAGTGCAGCTGAACGTCATCGCGACCCGGCAGTTGATATTACT<br>CGCGCAGCAGATGAAGAACTTAGAGGTCTTTATGCACGTCTCTACTGCATACGCCTACT<br>GCAACAGAAAGCACATCGATGAAGTAGTATATCCTCCTCCTGTAGATCCTAAGAAGCTTA<br>TCGATTCATTAGAGTGGATGGACGACGGCCTCGTCAACGACATAACACCAAAGCTGATT<br>GGCGACCGACCGAACACCTACATCTACACGAAGGCACTGGCTGAATATGTAGTACAACA<br>AGAAGGTGCTAAATTAAATGTAGCTATTGTAAGACCTTCAATTGTAGGTGCTTCATGGAA<br>AGAACCTTTCCCAGGCTGGATTGATAATTTCAACGGCCCTTCAGGTTTATTTATTGCTGC<br>TGGTAAAGGTATCTTGCGAACAATGAGAGCTTCAAATAATGCTTTAGCTGATTTAGTACC<br>TGTAGATGTAGTAGTAAATACATCATTAGCTGCTGCTTGGTACAGTGGCGTGAATCGTCC<br>ACGTAATATAATGGTATATAATTGCACAACAGGTTCAACAAATCCTTTCCACTGGGGTGA<br>AGTTGGAGACTACCTGAACCACAGCTTCAAGATGAACCCACTAAACCAGGTCTTTCGA<br>CACCCTTATGTCAAATTCTGCTCAAACAACCTGATGTTGCATTATTGGAAAGGTGTAAAG<br>CACACGGTACCTGCTTTATTATTAGATTTAGCTTTAAGATTAACAGGGCAGAAGCCTTGG<br>ATGATGAAGACTATCACTCGGCTACACAAGGCAATGGTCTTCCTTGAGTACTTTACCTCT<br>AACTCTTGGGTTTGGAACACCGACAACGTTAACATGCTTATGAACCAGCTGAACCCGGA<br>GGATAAGAAGACATTCAATATCGACGTTAGGCAGCTGCACTGGGCAGAGTACATAGAGA<br>ACTACTGCATGGGAACTAAGAAGTACGTATTAAATGAAGAGATGTCGGGCCTTCCGGCA<br>GCCCGAAAGCACTTAAACAAGCTCCGTAACATCCGTTACGGCTTTAATACTATCCTGGTA<br>ATCCTAATCTGGCGAATCTTCATCGCCCGCTCCCAGATGGCCCGGAATATATGGTATTTCG<br>TCGTTTCTCTGTGCTACAAGTTTCTAAGCTATTTCCGTGCATCATCAACAATGAGATATTG<br>A

TaFAR1	ATGGTTAGCATACCCGAGTACTACGAGGGTAAGAACATCCTGCTCACGGGCGCAACCGG<br>ATTTATGGGTAAGGTTCTCCTCGAGAAGCTTCTGCGTTCTTGCCCAAAGGTGAAGGCGG<br>TTTATGTATTAGTAAGACATAAAGCTGGTCAGACTCCGGAGGCAAGGATCGAAGAAATC<br>ACTAATTGCAAGTTGTTCGACCGGCTCCGCGACGAGCAGCCCGACTTTAAAGCAAAGAT<br>AATCGTGATAACGAGTGAGTTGACTCAGCCAGAGCTGGACTTGAGCGAGCCAATAAAG<br>GAGAAGCTCATAGAACGAATAAACATCATCTTCCACTGCGCTGCTACAGTAAGATTTAAT<br>GAAACATTAAGAGACGCCGTTCAGCTCAACGTCACTGCAACCCAGCAGTTGCTGTTTCT<br>CGCGCAGAGGATGAAGAACTTAGAGGTCTTCATGCACGTCTCGACGGCATACGCGTACT<br>GCAACCGGAAACAGATTGAGGAGATCGTTTACCCTCCTCCTGTAGATCCTAAGAAGCTT<br>ATAGACAGCTTAGAGTGGATGGACGACGGGTTGGTTAACGACATAACTCCTAAACTCAT<br>CGGCGACCGTCCGAACACTTACACTTACACCAAGGCGTTGGCTGAATATGTAGTACAAC<br>AAGAAGGTGCTAAATTAAATACAGCTATTATTAGACCTTCAATTGTAGGTGCTTCATGGA<br>AAGAACCTTTCCCGGGCTGGATCGATAATTTCAACGGGCCTTCAGGTTTATTTATTGCTG<br>CTGGTAAAGGTATCCTAAGGACAATGAGAGCTTCAAATTCAGCTGTAGCTGATTTAGTAC<br>CTGTAGATGTAGTAGTAAATACAACATTAGCTGCTGCTTGGTATTCAGGTGTAAATAGAC<br>CTAGAAATGTAATGATTTATAATTGTACAACAGGTGGTACAAACCCGTTTCATTGGGGGG<br>AAGTCGGCTACCACATAAACCTAAACTTTAAGATCAATCCCTTGGAGAACGCGGTAAGA<br>CATCCTAATTGTTCATTACAATCAAATCCTTTATTACATCAATATTGGACAGCGGTTAGCC<br>ACACCATGCCCGCGTTTCTGCTAGACCTGCTGCTACGACTTACAGGACACAAGCCATGG<br>ATGATGAAGACCATCACTCGGTTGCACAAGGCAATGATGTTGCTAGAGTACTTTACCAG<br>CAACAGCTGGATCTGGAACACGGAGAACATGACTATGCTGATGAACCAGCTAAACCCA<br>GAGGATAAGAAGACTTTCAATTTCGACGTTCGGCAGCTCCATTGGGCAGAGTACATGGA<br>GAACTACTGTATGGGTACTAAGAAGTACGTGTTAAATGAAGAGATGTCTGGCCTCCCAG<br>CCGCCCGTAAACATCTAAACAAGTTGCGTAACATCCGTTACGGATTTAATACTGTCCTAG<br>TGATCTTGATCTGGAGGATATTCATCGCCAGGTCTCAGATGGCACGTAACATATGGTACT<br>TTGTGGTATCTCTTTGCTACAAGTTTCTCTCTTATTTCCGGGCCTCATCAACAATGAGATA<br>TTGA

MCRN	ATGTCTGGAACTGGTAGGTTGGCCGGAAAGATCGCTTTGATCACCGGAGGAGCCGGAA<br>ATATCGGAAGTGAATTGACTAGAAGATTCTTGGCCGAGGGAGCTACTGTTATCATCTCTG<br>GTAGGAACAGAGCTAAGTTGACCGCCTTGGCTGAAAGAATGCAAGCTGAGGCCGGTGT<br>CCCAGCTAAAAGGATCGACCTTGAGGTCATGGACGGTTCTGACCCAGTTGCTGTTAGAG<br>CTGGTATCGAAGCTATTGTTGCTAGACACGGTCAGATTGACATTCTTGTCAATAACGCCG<br>GTTCTGCCGGAGCCCAAAGGAGATTGGCCGAGATCCCACTTACCGAAGCTGAATTGGG<br>TCCCGGTGCTGAAGAAACCTTGCACGCCAGTATCGCCAATCTTTTGGGAATGGGTTGGC<br>ACCTTATGAGGATTGCTGCCCCTCATATGCCAGTCGGATCTGCTGTCATCAACGTCAGTA<br>CCATCTTTTCTAGGGCCGAGTATTACGGTAGGATCCCATACGTTACCCCAAAGGCCGCCT<br>TGAATGCTTTGTCTCAACTTGCCGCCAGAGAGTTGGGTGCCAGAGGTATTAGGGTTAAC<br>ACCATCTTCCCCGGTCCAATCGAGAGTGATAGAATCAGAACCGTCTTCCAAAGGATGGA<br>CCAGTTGAAAGGTAGGCCAGAAGGAGACACCGCCCATCATTTCTTGAACACCATGAGG<br>CTTTGTAGAGCCAACGACCAAGGTGCCCTTGAAAGGAGATTCCCTTCTGTCGGAGATGT<br>TGCTGATGCTGCCGTCTTCCTTGCCTCTGCCGAATCTGCCGCCCTTTCTGGTGAGACCAT<br>CGAGGTCACCCATGGTATGGAGCTTCCAGCTTGTAGTGAGACTAGTCTTCTTGCTAGAA<br>CCGACTTGAGAACTATCGACGCCAGTGGAAGAACCACCTTGATCTGTGCCGGAGACCA<br>AATCGAGGAGGTCATGGCCCTTACTGGTATGCTTAGGACTTGTGGAAGTGAGGTTATCAT<br>CGGTTTTAGAAGTGCTGCCGCTCTTGCCCAGTTCGAGCAAGCCGTTAATGAGAGTAGGA<br>GGTTGGCTGGAGCCGACTTCACCCCTCCTATTGCCCTTCCTTTGGACCCAAGAGACCCA<br>GCCACCATCGATGCCGTCTTTGACTGGGCTGGTGAGAATACTGGTGGTATTCACGCCGC<br>TGTTATCTTGCCAGCTACCAGTCACGAACCAGCTCCATGCGTTATTGAGGTCGATGACGA<br>GAGGGTCTTGAACTTTTTGGCCGACGAAATCACCGGTACCATCGTCATTGCTTCTAGGTT<br>GGCTAGATACTGGCAAAGTCAGAGACTTACCCCCGGTGCTAGGGCTAGGGGTCCTAGGG<br>TCATCTTCTTGTCTAACGGTGCCGACCAGAACGGAAACGTCTACGGAAGGATCCAGAGT<br>GCCGCCATCGGTCAGCTTATTAGAGTCTGGAGACATGAGGCCGAATTGGACTATCAGAG<br>AGCCTCTGCTGCTGGTGACCATGTTTTGCCACCAGTTTGGGCCAACCAGATCGTTAGAT<br>TCGCCAATAGAAGTCTTGAAGGATTGGAGTTCGCTTGTGCTTGGACTGCCCAGCTTTTG<br>CACAGTCAGAGGCACATCAATGAGATTACCTTGAATATCCCAGCTAATATTTAA

MCRC	ATGTCTGCTACCACTGGTGCTAGGTCTGCTTCTGTCGGTTGGGCTGAGTCTTTGATCGGT<br>TTGCACCTTGGAAAAGTCGCCCTTATCACCGGAGGATCTGCCGGTATTGGTGGTCAGAT<br>CGGTAGGCTTTTGGCTTTGAGTGGAGCTAGAGTCATGTTGGCTGCTAGAGATAGACATA<br>AGCTTGAGCAGATGCAAGCCATGATCCAGTCTGAGCTTGCCGAGGTCGGATACACTGAC<br>GTCGAGGACAGAGTCCATATTGCCCCCGGTTGCGACGTCAGTAGTGAAGCCCAACTTGC<br>CGACTTGGTCGAAAGGACCCTTTCTGCCTTTGGAACCGTCGACTATTTGATCAACAATG<br>CCGGTATTGCCGGTGTCGAAGAAATGGTCATCGACATGCCAGTCGAGGGATGGAGGCA<br>CACCTTGTTTGCCAACTTGATTAGTAATTATTCTTTGATGAGGAAGCTTGCCCCACTTATG<br>AAGAAGCAAGGTTCTGGTTATATTCTTAATGTCAGTTCTTATTTCGGTGGAGAGAAGGAC<br>GCCGCCATTCCATACCCAAATAGGGCTGACTACGCCGTTAGTAAGGCTGGACAGAGAGC<br>CATGGCTGAAGTCTTTGCTAGATTCTTGGGTCCAGAGATCCAGATCAATGCCATCGCCCC<br>CGGTCCAGTTGAGGGAGATAGACTTAGGGGAACTGGTGAAAGGCCCGGTCTTTTCGCT<br>AGGAGGGCTAGACTTATCCTTGAGAATAAGAGGCTTAACGAACTTCACGCCGCTCTTAT<br>CGCTGCTGCCAGAACCGATGAGAGGAGTATGCACGAGTTGGTCGAATTGCTTTTGCCAA<br>ACGACGTTGCCGCCTTGGAACAGAATCCAGCTGCCCCAACTGCTCTTAGGGAGTTGGCT<br>AGAAGGTTTAGAAGTGAAGGAGATCCAGCCGCCTCTTCTAGTTCTGCTTTGCTTAATAG<br>ATCTATCGCCGCCAAGCTTTTGGCCAGACTTCACAACGGTGGATACGTTTTGCCAGCCG<br>ACATCTTCGCTAACTTGCCAAACCCACCAGACCCTTTCTTCACCAGAGCCCAAATCGAT<br>AGAGAGGCTAGAAAGGTTAGAGACGGTATCATGGGAATGTTGTACTTGCAGAGGATGCC<br>AACCGAATTTGATGTTGCCATGGCTACCGTTTACTACTTGGCTGATAGGGTCGTCTCTGG<br>TGAAACCTTCCATCCAAGTGGAGGTTTGAGGTACGAGAGGACCCCAACCGGAGGAGAG<br>CTTTTCGGTCTTCCTTCTCCAGAGAGGTTGGCCGAATTGGTCGGTTCTACCGTCTACCTT<br>ATTGGAGAGCACTTGACCGAACATTTGAATTTGCTTGCTAGAGCCTACCTTGAAAGGTA<br>CGGAGCCAGACAAGTTGTTATGATCGTCGAAACCGAGACTGGAGCTGAGACCATGAGG<br>AGACTTCTTCACGACCACGTTGAGGCTGGAAGATTGATGACCATCGTCGCTGGAGACCA<br>GATTGAGGCTGCCATCGACCAAGCTATCACTAGATATGGAAGACCCGGTCCAGTTGTCT<br>GTACTCCTTTCAGACCTCTTCCAACCGTCCCTTTGGTCGGTAGGAAGGATAGTGACTGG<br>TCTACCGTCTTGAGTGAGGCCGAATTCGCCGAACTTTGCGAACACCAACTTACCCACCA<br>TTTCAGAGTCGCTAGATGGATTGCTCTTAGTGATGGTGCCAGACTTGCTTTGGTCACTCC<br>AGAAACCACCGCCACCAGTACCACCGAACAGTTCGCCTTGGCCAACTTCATCAAGACT<br>ACCTTGCACGCTTTCACCGCCACTATTGGAGTTGAGTCTGAAAGGACCGCCCAGAGGAT<br>CTTGATCAACCAAGTTGACCTTACCAGAAGGGCTAGGGCCGAGGAACCTAGAGATCCT<br>CACGAAAGGCAGCAAGAACTTGAGAGGTTCATCGAGGCCGTCCTTTTGGTTACTGCTC<br>CTTTGCCTCCAGAAGCCGATACCAGATATGCTGGAAGGATCCATAGAGGAAGAGCTATC<br>ACTGTCTAA

Reference

1. Valle-Rodríguez JO, Shi S, Siewers V, Nielsen J. Metabolic engineering of Saccharomyces cerevisiae for production of fatty acid ethyl esters, an advanced biofuel, by eliminating non-essential fatty acid utilization pathways. Appl. Energy. 2014; 115:226-232.

2. Zhou YJ, Buijs NA, Zhu Z, Gómez DO, Boonsombuti A, Siewers V *et al*. Harnessing yeast peroxisomes for biosynthesis of fatty-acid-derived biofuels and chemicals with relieved side-pathway competition. J Am Chem Soc. 2016; 138(47):15368-15377.

3. Yu T, Zhou YJ, Huang M, Liu Q, Pereira R, David F *et al*. Reprogramming yeast metabolism from alcoholic fermentation to lipogenesis. Cell. 2018; 174(6):1549-1558.

4. Cao X, Yu W, Chen Y, Yang S, Zhao ZK, Nielsen J, Luan H, and Zhou YJ. Engineering yeast for high-level production of diterpenoid sclareol. Metabolic Engineering. 2023; 75:19-28.

5. Yu W, Cao X, Gao J, and Zhou YJ. Overproduction of 3-hydroxypropionate in a super yeast chassis. Bioresource Technology. 2022; 361: 127690.
